# Supplementary material for: Messinian vegetation and climate of the intermontane Florina–Ptolemais–Servia Basin, NW Greece inferred from palaeobotanical data: how well do plant fossils reflect past environments?
Source: R Soc Open Sci. 2020 May 27;7(5):192067. doi: 10.1098/rsos.192067 (PMC7277258; doi:10.1098/rsos.192067)
Supplement: Supplementary Material [file rsos192067supp1.zip › Supplementary Material S1-S5/S5_CLAMP_Vegora/CLAMP analysis/Info.docx]

1. *Daphnogene pannonica* : no any sample on plates.
2. *Platanus* sp. : no any sample on plates
3. *Fagus decurrens* : no any sample on plates
