# Supplementary material for: Messinian vegetation and climate of the intermontane Florina–Ptolemais–Servia Basin, NW Greece inferred from palaeobotanical data: how well do plant fossils reflect past environments?
Source: R Soc Open Sci. 2020 May 27;7(5):192067. doi: 10.1098/rsos.192067 (PMC7277258; doi:10.1098/rsos.192067)
Supplement: Supplementary Material [file rsos192067supp1.zip › Supplementary Material S1-S5/S5_CLAMP_Vegora/CLAMP analysis/Run/PhysgAsia1_HiResGridMetAsia1/Results.html]

CLAMP\_Analysis\_Online

|  |  |  |  |  |  |  |
| --- | --- | --- | --- | --- | --- | --- |
| |  |  |  |  | | --- | --- | --- | --- | | CLAMP\_Analysis\_Online |  |  |  | | | |
| --- |

|  |
| --- |
| Results for submission: 336F83E04F31BF22 |
| CCA1vsCCA2.jpg  CCA1vsCCA3.jpg  CCA2vsCCA3.jpg  CCA3d.jpg  CCAGraph.pdf  GSP.jpg  MAT.jpg  models.pdf  params\_336F83E04F31BF22.txt  predict.csv  scoreres.csv  Vegora.csv | Parameter values: withlabel = true  calibration\_option = 5  image\_type = colour  file\_uploaded = Vegora.csv |
|  | |
|  | |
|  | |
|  | |
|  | |
|  | |
