# Supplementary material for: Messinian vegetation and climate of the intermontane Florina–Ptolemais–Servia Basin, NW Greece inferred from palaeobotanical data: how well do plant fossils reflect past environments?
Source: R Soc Open Sci. 2020 May 27;7(5):192067. doi: 10.1098/rsos.192067 (PMC7277258; doi:10.1098/rsos.192067)
Supplement: Supplementary Material [file rsos192067supp1.zip › Supplementary Material S1-S5/S3_Taxonomic_description_pollen.pdf]

Supplementary Material S3: Systematic palaeobotany and descriptions of palynomorphs from the plant fossil bearing strata of Vegora (sample S115992)

The systematic palaeobotany section starts with algae and is followed by fern and fern allies, gymnosperms, and angiosperms. Angiosperm classification and author names of orders and families follow APG IV [1]. LM and SEM in the descriptions refers to light microscopy and scanning electron microscopy observations, respectively.

(a) Algae

Family Botryococcaceae
Genus *Botryococcus* Kürtzing
Botryococcus sp. cf. *B. braunii* (Fig. 3a)
Remarks: Similar structures are produced by extant *Botryococcus braunii* Kürtzing. *Botryococcus* is commonly found in freshwater but can thrive also in brackish conditions.

Family Zygnemataceae Kützning
Genus *Spirogyra* Link in C.G. Nees/Fossil genus *Ovoidites* R.Potonié emend. Krutzsch
Spirogyra sp. 1/*Ovoidites elongatus* (Hunger) Krutzsch (Fig. 3b)
Description: Aplanospore or zygospore, outline elliptic to broadly ovoidal, palynomorph size large, length of axis perpendicular to fissure 70–100 µm (LM); mesospore 1–1.5 µm thick (LM); sculpturing psilate (LM, SEM), if ruptured gap spanning from pole to pole.

Remarks: *Ovoidites elongatus* is commonly associated with *Spirogyra*, indicating shallow, stagnant, oxygen-rich open freshwater and lake margins [2].

Fossil genus *Cycloovoidites* Krutzsch & Pacltová
Spirogyra sp. 2/*Cycloovoidites cyclus* (Krutzsch) Krutzsch & Pacltová (Fig. 3c)
Description: Aplanospore or zygospore, outline circular, palynomorph size large to very large, diameter 90–110 µm (LM); mesospore 2–2.4 µm thick (LM); sculpturing rugulate, verrucate (LM, SEM).

Remarks: *Cycloovoidites cyclus* is commonly associated with *Spirogyra* [2, 3].

(b) Fern and Fern allies

Order Osmundales Bromhead
Family Osmundaceae Marinov/Fossil genus *Baculatisporites* Pflug & P.W.Thomson in P.W.Thomson & Pflug
Genus *Osmunda* L.
Osmunda sp. (Figs 3d–e)
Description: Spore, monad, shape spheroidal to oblate, amb circular; spore size medium to large, equatorial diameter 30–65 µm (LM, SEM); exospore 1–1.5 µm thick (LM), 2–3 µm thick including sculpture elements (LM); trilete, laesurae 2/3 to 3/4 of spore radius; sculpturing baculate to rugulate (LM), sculpturing present on distal and proximal face.

Remarks: Extant *Osmunda* produces morphologically identical spores [4, 5]. Specimens identified from the Vegora sample fall within the range of the fossil form species *Baculatisporites major* (Raatz) Krutzsch (Supplementary Figs 1a–c), *B. primarius* (Wolff) P.W.Thomson & Pflug and *B. nanus* (Wolff) Krutzsch. [6, 7].

Order Polypodiales Link
Family Pteridaceae E.D.M.Kirchn.
Genus *Cryptogramma* R.Brown/*Cheilanthes* Sw./Fossil genus *Cryptogrammosporis* Skawińska
Cryptogramma vel *Cheilanthes* sp./*Cryptogrammosporis magnoides* (Krutzsch) Skawińska (Figs 3f; Supplementary Figs 1d–f)
Description: Spore, monad, shape oblate, amb triangular; spore size medium, equatorial diameter 35–50 µm (LM); exospore 1–1.5 µm thick (LM), < 2.5 µm thick including sculpture elements (LM); trilete, laesurae 2/3 to 3/4 of spore radius; sculpturing verrucate (LM), sculpturing less prominent on proximal face.

Remarks: Extant *Cryptogramma* [5] and *Cheilanthes* [8] produce morphologically similar spores.

Genus *Pteris* L./*Polypodiaceoisporites* R.Potonié
Pteris sp./*Polypodiaceoisporites corrutoratus* Nagy (Figs 3g–h; Supplementary Figs 1g–i)

Description: Spore, monad, shape oblate, amb convex triangular; spore size medium, equatorial diameter 25–30 μm (LM); exospore 0.5–1 μm thick (LM), < 3.5 μm thick including sculpture elements (LM); trilete, laesurae 2/3 to 3/4 of spore radius, cingulum present; sculpturing bacculate (LM), sculpturing less prominent on proximal face.

Remarks: Several extant *Pteris* species produce morphologically similar spores, e.g. *P. dentata* Forssk., *P. ensiformis* Burm. [5, 9].

Family Davalliaceae M.R.Schomb./Polypodiaceae J.Presl & C.Presl
Fossil genus *Verrucatosporites* Pflug & P.W.Thomson in P.W.Thomson & Pflug
Davalliaceae vel Polypodiaceae/*Verrucatosporites alienus* (R.Potonié) P.W.Thomson & Pflug (Fig. 3i)
Description: Spore, monad, shape oblate, amb elliptic, outline elliptic to renal-shaped in equatorial view; spore size medium to large, equatorial diameter 50–60 μm (LM); exospore 1.5–3.5 μm thick including sculpture elements (LM); monolete, laesurae 1/2 to 2/3 of spore radius; sculpturing verrucate (LM), sculpturing less prominent on proximal face.

Remarks: Morphologically similar spores are found in *Davallia* Sm., *Microgramma* C.Presl, *Pleopeltis* Humb. & Bonpl. ex Willd., and *Polypodium* L. [5].

Incerta sedis
Fossil genus *Laevigatosporites* Ibrahim
Monolete spore fam. indet./*Laevigatosporites haardti* (R.Potonié & Venitz) P.W.Thomson & Pflug (Fig. 3j; Supplementary Figs 1j–l)
Description: Spore, monad, shape oblate, amb elliptic, outline elliptic to renal-shaped in equatorial view; spore size medium, equatorial diameter 25–50 μm (LM); exospore 1–1.5 μm thick (LM); monolete, laesurae 1/2 to 2/3 of spore radius; sculpturing psilate (LM).

Remarks: Morphologically similar monolete and psilate spores are found in several extant fern families (e.g. Aspleniaceae, Davalliaceae, Dryopteridaceae, Gleicheniaceae, Lomariopsidaceae, Oleandraceae, Thelypteridaceae, Vittariaceae, Polypodiaceae, see [5, 7]).

(c) Gymnosperms

Family Cupressaceae Rich. ex Bartling

Papillate Cupressaceae pollen/*Inaperturopollenites hiatus* (R.Potonié) Thomson & Pflug

(Figs 3k–l; Supplementary Figs 1m–o)

Description: Pollen, monad, spheroidal, outline circular; pollen size medium, diameter 25–50 µm (LM); exine 1–1.5 µm thick (LM); leptoma with papilla, radially split with papilla at the end of rupture; sculpturing scabrate (LM), microverrucate, nanoechinate (SEM), orbiculae present.

Remarks: Split papillate Cupressaceae pollen is generally assigned to *Inaperturopollenites hiatus* [10, 11]. Pollen similar to the figured specimen is produced by extant members of subfamilies Sequoioideae and Taxodioideae [12, 13, 14]. Ruptured pollen of these subfamilies is stenopalynous and lacks distinct characteristics for further determination.

Supplementary Figure 1. Light microscopy (LM) and scanning electron microscopy (SEM) micrographs of fern and gymnosperm palynomorphs.

(a–c) *Osmunda* sp./*Baculatisporites major*, (a) PV, (b, c) DV, (c) exospore sculpture detail. (d–f) *Cryptogramma* vel *Cheilanthes* sp./*Cryptogrammosporis magnoides*, (d) PV, (e, f) PRV, (f) exospore sculpture detail. (g–i) *Pteris* sp./*Polypodiaceoisporites corrutoratus*, (g) PV, (h, f) DV, (f) exospore sculpture detail. (j–l) Monolete spore fam. indet. sp./*Laevigatosporites haardti*, EV, (f) exospore sculpture detail. (m–o) Papillate Cupressaceae pollen/*Inaperturopollenites hiatus*, (m) PV, (n, o) PRV, (o) exine sculpture detail.

Abbreviations: equatorial view (EV), polar view (PV), distal view (DV), proximal view (PRV). Scale bars 10 µm (a, b, d, e, g, h, j, k, m, n), 1 µm (c, f, i, l, o).

Supplementary Figure 1. Light microscopy (LM) and scanning electron microscopy (SEM) micrographs of fern and gymnosperm palynomorphs.

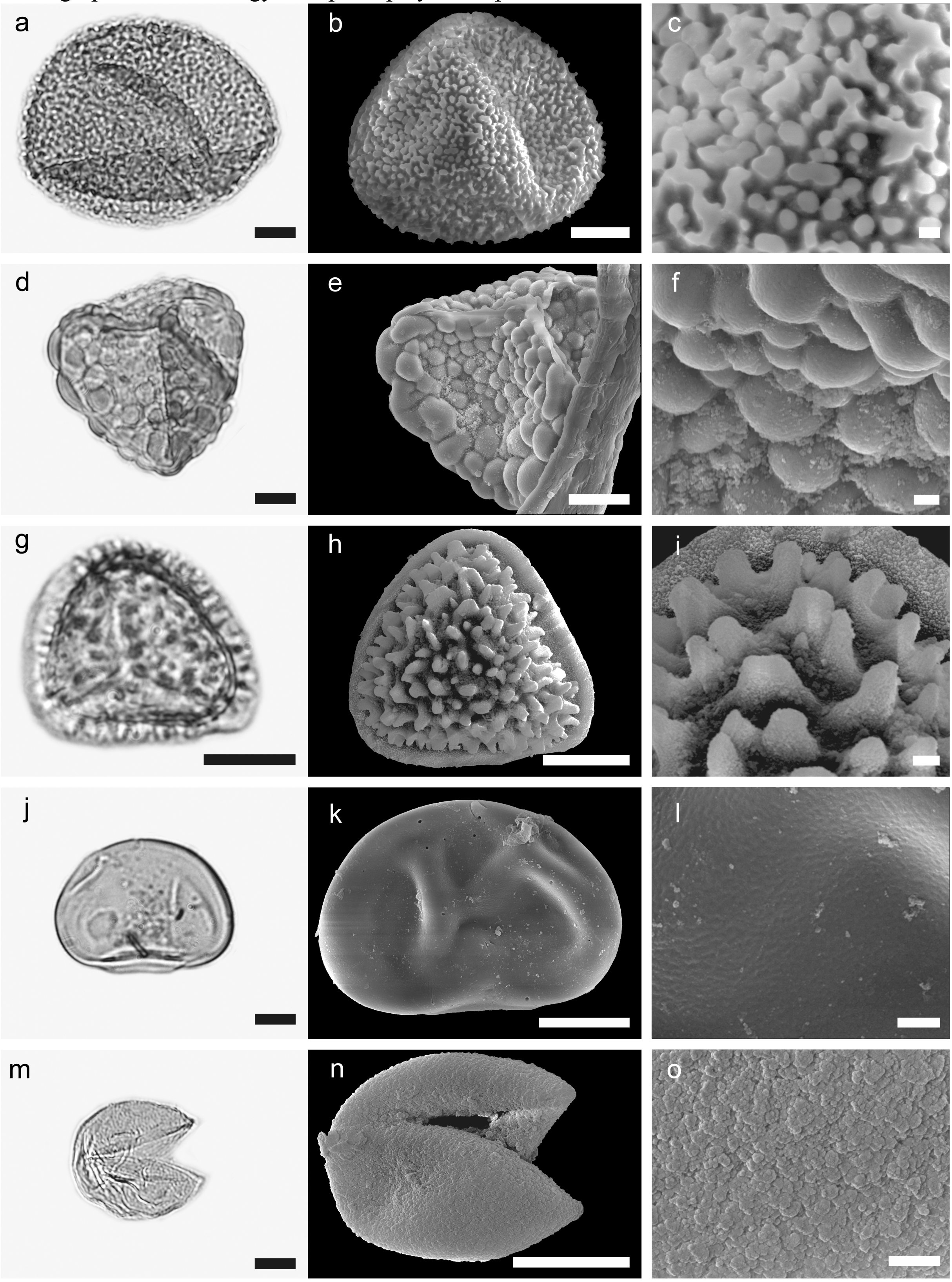

Family Pinaceae Spreng. ex F.Rudolphi

Genus *Abies* Mill.

Abies sp. (Fig. 3m; Supplementary Figs 2a–d)

Description: Pollen, monad, bisaccate, shape oblate, outline elliptical in polar view, pollen size large to very large, diameter 70–120 µm (LM); exine 1.5–2 in cappa region 3–5 µm thick (*Abies*-crest in LM); leptoma; sacci nearly spherical.

Remarks: The figured specimen corresponds (e.g. crest, sacci nearly spherical, size) to extant pollen of *Abies* [15].

Genus *Cathaya* Chun & Kuang

Cathaya sp. (Figs 3n–o; Supplementary Figs 2e–h)

Description: Pollen, monad, bisaccate, shape oblate, outline elliptical in polar view, pollen size large, diameter 60–80 µm (LM); exine 1–1.5 µm thick (LM); leptoma; sacci half-spherical; exine sculpturing nanoechinate (SEM).

Remarks: Nanoechinate exine sculpturing (Fig. 3o) is a characteristic feature of extant *Cathaya* pollen [16,17].

Genus *Cedrus* Trew

Cedrus sp. (Fig. 3p)

Description: Pollen, monad, bisaccate, shape oblate, outline elliptical in polar view, pollen size large, diameter 60–80 µm (LM); exine 1–1.5 µm thick, in cappa region < 3 µm thick (LM); leptoma; sacci half-spherical, sacci attachment on proximal face thickened.

Remarks: The figured pollen resembles extant *Cedrus libani* A.Rich. and *C. deodora* (Roxb. ex D.Don) G.Don [18].

Genus *Pinus* L.

Pinus subgenus *Pinus* L.

Pinus subgenus *Pinus* sp. (Fig. 3q)

Description: Pollen, monad, bisaccate, shape oblate, outline elliptical in polar view, pollen size large, diameter 60–80 µm (LM); exine 1–1.5 µm thick (LM); leptoma; sacci nearly spherical, sacci attachment narrow.

Remarks: Pollen of *Pinus* subgenus *Pinus* sp. (diploxylon type) is characterized by narrowly attached and spherical sacci [18].

Pinus subgenus *Strobus* Lemmon

Pinus subgenus *Strobus* sp. (Fig. 3r)

Description: Pollen, monad, bisaccate, shape oblate, outline elliptical in polar view, pollen size large, diameter 60–80 µm (LM); exine 1–1.5 µm thick (LM); leptoma; sacci half-spherical, sacci attachment broad.

Remarks: Pollen of *Pinus* subgenus *Pinus* sp. (haploxylon type) is characterized by broadly attached, half-spherical sacci and dotted thickenings in the leptoma area [19].

Genus *Tsuga* (Endl.) Carrière

Tsuga sp. 1 (Figs 3s–t; Supplementary Figs 2m–p)

Description: Pollen, monad, monosaccate, shape oblate, outline circular in polar view, elliptic in equatorial view, pollen size medium to large, equatorial diameter 45–60 µm (LM); saccus 3–5 µm wide (LM); leptoma; sculpturing verrucate, rugulate, echinate (LM), echini length 1.5–3 µm, echini equally distributed.

Remarks: Morphologically similar echinate pollen with a relatively narrow saccus is produced by extant *Tsuga dumosa* Eichl. [20].

Tsuga sp. 2 (Figs 3u–v; Supplementary Figs 2i–l)

Description: Pollen, monad, monosaccate, shape oblate, outline circular in polar view, elliptic in equatorial view, pollen size large, equatorial diameter 60–80 µm (LM); saccus 5–10 µm wide (LM); leptoma; sculpturing verrucate, rugulate, echinate (LM), echini length 1–2.5 µm, echini density lower in saccus area.

Remarks: *Tsuga* sp. 2 differs from *Tsuga* sp. 1 by larger size and a broader saccus. Morphologically similar echinate pollen with a relatively broad saccus is produced by extant *Tsuga forrestii* Downie and *T. chinensis* (Franch.) E.Pritz. in Diels [20].

(d) Angiosperms

Order Poales Small

Family Typhaceae Juss.

Genus *Typha* L./Fossil genus *Tetradomonoporites* Chitaley

Typha sp./*Tetradomonoporites typhoides* Krutzsch (Fig. 4a)

Description: Pollen, permanent tetrad, planar tetrad, monad shape spheroidal to subspheroidal, outline circular, tetrad size medium, monad size small, monad diameter 18–25 µm (LM); porate, porus sunken; sculpturing reticulate (LM, SEM).

Remarks: Within Typhaceae, only *Typha* disperses pollen in permanent tetrads [15, 21].

Family Poaceae Barnhart

Poaceae gen. indet. (Figs 4b–c; Supplementary Figs 2q–s)

Description: Pollen, monad, shape spheroidal, outline circular, pollen size small, monad diameter 20–25 µm (LM); porate, porus annulate; sculpturing scabrate (LM), nanoechinate, nanoechini weakly grouped in areolae (SEM).

Remarks: Areolate exine sculpturing is widely present in several Poaceae subfamilies (e.g. *Lolium* spp., *Phleum* spp., *Poa* spp. [22]).

Supplementary Figure 2. Light microscopy (LM) and scanning electron microscopy (SEM) micrographs of gymnosperm and Poaceae palynomorphs.

(a–d) *Abies* sp., EV, (c) corpus exine sculpture detail, (d) sacci exine sculpture detail. (e–h) *Cathaya* sp., (e) PV, (f–h) PRV, (g) corpus exine sculpture detail, (h) sacci exine sculpture detail. (i–l) *Tsuga* sp. 2, (i) PV, (j–l) PRV, (k) corpus exine sculpture detail, (l) sacci exine sculpture detail. (m–p) *Tsuga* sp. 1, (m) PV, (n–p) PRV, (k) corpus exine sculpture detail, (l) sacci exine sculpture detail. (q–s) Poaceae gen. indet., (q) PV, (r, s) PRV, (s) exine sculpture detail.

Abbreviations: equatorial view (EV), polar view (PV), distal view (DV), proximal view (PRV). Scale bars 10 µm (a, b, e, f, i, j, m, n, q, r), 1 µm (c, d, g, h, k, l, o, p, s).

Supplementary Figure 2. Light microscopy (LM) and scanning electron microscopy (SEM) micrographs of gymnosperm and poaceae palynomorphs.

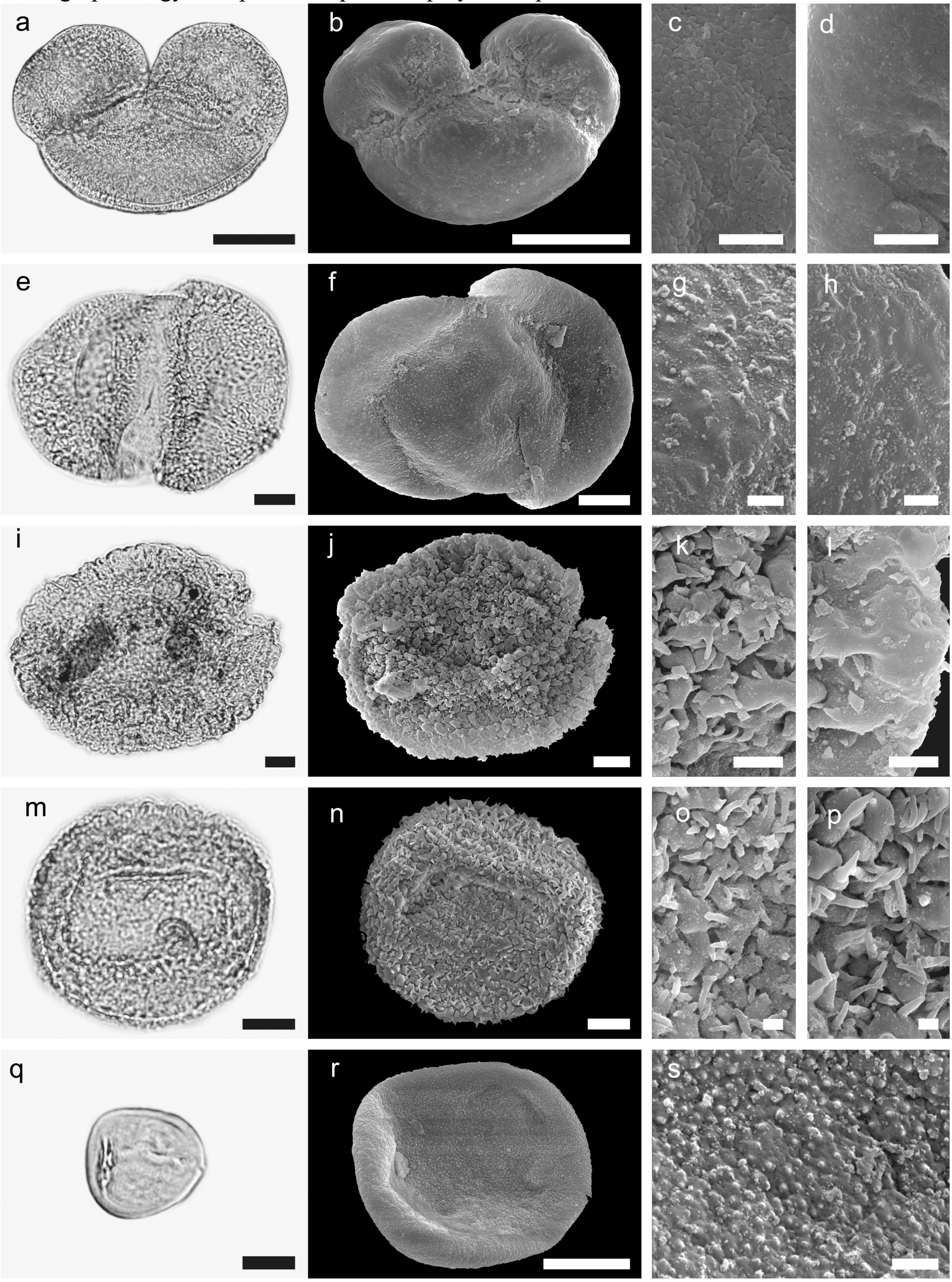

Order Vitales Juss. ex Bercht. & J.Presl

Family Vitaceae Juss.

Genus *Parthenocissus* Planch.

Parthenocissus sp. (Figs 4f–g; Supplementary Figs 3d–f)

Description: Pollen, monad, shape prolate, outline elliptic in equatorial view, pollen size medium, diameter 18–25 µm (LM), polar axis 30–35 µm (LM); tricolporate, endoporus lalongate elliptic; sculpturing reticulate (LM, SEM), lumen size decreasing towards colpi (SEM).

Remarks: Pollen similar to the figured specimen is found in extant e.g. *Parthenocissus sinensis* Diels & Gilg ex Diels or *P. heptaphylla* (Planch.) Britton [23].

Order Rosales Bercht. & J.Presl

Family Ulmaceae Mirbel

Genus *Ulmus* L./*Zelkova* Spach.

Ulmus vel *Zelkova* sp. (Fig. 4h; Supplementary Figs 3g–i)

Description: Pollen, monad, shape oblate, outline weakly circular to polygonal in polar view, pollen size medium, diameter 35–55 µm (LM); porate, in some specimens annulus present; sculpturing rugulate (LM).

Remarks: *Ulmus* and *Zelkova* share overlapping morphological characters [24], the encountered specimens display poor preservation. Both *Ulmus* and *Zelkova* are present in the Vegora fossil fleaf record.

Order Fagales Engler

Family Fagaceae Dumort.

Genus *Fagus* sp. (Fig. 4i; Supplementary Figs 3j–l)

Description: Pollen, monad, shape spheroidal to subprolate, outline circular in polar view, circular to elliptic in equatorial view, pollen size medium, polar axis 35–50 µm long (LM); tricolporate; sculpturing scabrate (LM), rugulate, fossulate, rugulae often protruding and diverging (SEM).

Remarks: Modern pollen of *Fagus* subgenus *Fagus* displays similar size ranges and colpi length ranging from half to two thirds of the polar axis [25, 26].

Genus *Quercus* L.

Quercus subgenus *Cerris* sect. *Cerris* Oerst.

Quercus sect. *Cerris* sp. (Figs 4j–k; Supplementary Figs 3m–o)

Description: Pollen, monad, shape prolate, outline lobate in polar view, elliptic in equatorial view, pollen size medium, polar axis 30–40 µm long (LM), diameter; tricolpate; sculpturing scabrate (LM), mircro- to nanorugulate, fossulate, perforate, pollen surface irregularly covered by tufts and agglomerations of rodlet tufts.

Remarks: Generally, the ectexine of *Quercus* is composed of rod-like (micro) rugulae [25]. Sculpturing consisting of rugulae masked by secondary sporopollenin and tufts or agglomerations of tufts is characteristic for pollen of *Quercus* sect. *Cerris* [25].

Supplementary Figure 3. Light microscopy (LM) and scanning electron microscopy (SEM) micrographs of not identified Monocotyledonae, Vitaceae, Ulmaceae, and Fagaceae pollen.

(a–c) Monocotyledonae indet., (a) PV, (b, c) PRV, (c) exine sculpture detail. (d–f) *Parthenocissus* sp., EV, (f) exine sculpture detail. (g–i) *Ulmus* vel *Zelkova* sp., EV, (i) exine sculpture detail. (j–l) *Fagus* sp., EV, (l) exine sculpture detail. (m–o) *Quercus* sect. *Cerris* sp., EV, (o) exine sculpture detail.

Abbreviations: equatorial view (EV), polar view (PV), distal view (DV), proximal view (PRV). Scale bars 10 µm (a, b, d, e, g, h, j, k, m, n), 1 µm (c, f, i, l, o).

Supplementary Figure 3. Light microscopy (LM) and scanning electron microscopy (SEM) micrographs of not identified Monocotyledonae, Vitaceae, Ulmaceae, and Fagaceae pollen.

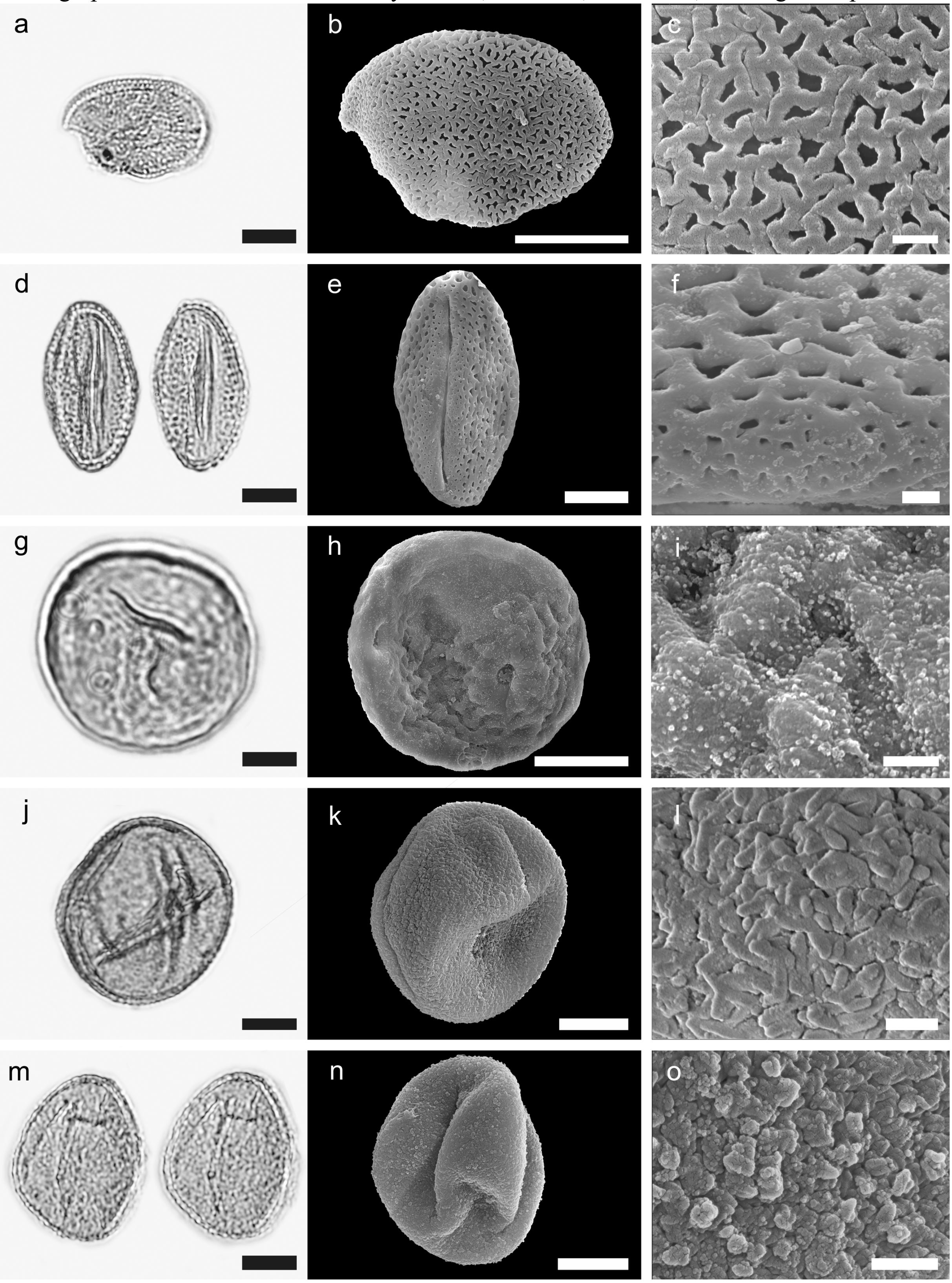

Quercus subgenus *Cerris* sect. *Ilex* Loudon

Quercus sect. *Ilex* sp. (Figs 4l–m; Supplementary Figs 4a–c)

Description: Pollen, monad, shape prolate, outline lobate in polar view, elliptic in equatorial view, pollen size small to medium, diameter 20–30 µm (LM); tricolpate; sculpturing scabrate (LM), micro- to nanorugulate, fossulate (SEM).

Remarks: Sculpturing consisting of rod-like micro to nanorugulae is characteristic of *Quercus* sect. *Ilex* [25].

Quercus subgenus *Quercus* sect. *Quercus* Loudon

Quercus sect. *Quercus* sp. (Figs 4n–o; Supplementary Figs 4d–f)

Description: Pollen, monad, shape prolate to subprolate, outline lobate in polar view, elliptic sub cyclic in equatorial view, pollen size medium, diameter 30–45 µm (LM); tricolpate; sculpturing scabrate (LM), micro- to nanoverrucate, fossulate, perforate, sculpture elements cauliflower-like (SEM).

Remarks: Cauliflower-like exine sculpturing is characteristic for pollen belonging to this section [25].

Subfamily Castaneoideae

Castaneoideae gen. indet. (Figs 4p–q; Supplementary Figs 4g–i)

Description: Pollen, monad, shape prolate, outline circular in polar view, elliptic in equatorial view, pollen size small, diameter 10–15 µm (LM), polar axis 18–25 µm (LM); tricolporate; sculpturing scabrate (LM), rugulate to microrugulate, perforate (SEM), rugulae covered with secondary striation (microrugulae), perforations encircled by triangular units formed by rugulae (SEM).

Remarks: Within the paraphyletic Castaneoideae pollen morphology is highly stenopalynous with several overlapping morphological characteristics, therefore generic determination is not possible in LM and SEM investigation. Similar exine sculpture (secondary striation on rugulae, triangular units) is present in pollen of extant *Castanopsis sieboldii* (Makino) Hatus. (=*C. cuspidata* var. *sieboldii*) [27].

Family Juglandaceae DC. ex Perleb

Subfamily Juglandoideae

Genus *Carya* Nutt.

Carya sp. (Fig. 4r; Supplementary Figs 4j–l)

Description: Pollen, monad, shape oblate, outline circular to convex triangular in polar view, pollen size medium, diameter 35–50 µm (LM); triporate, pori sunken; sculpturing scabrate (LM), nanoechinate (SEM).

Remarks: Oblate pollen with three pores off-set towards the distal pole is characteristic for this genus [28].

Genus *Platycarya* Siebold & Zucc.

Platycarya sp. (Fig. 4s; Supplementary Figs 4m–o)

Description: Pollen, monad, shape oblate, outline circular to convex triangular in polar view, pollen size small, diameter 18–25 µm (LM); triporate, stephanoporate, pori sunken; sculpturing scabrate, (LM), nanoechinate (SEM), pseudocolpi present on distal and proximal face (LM, SEM).

Remarks: *Platycarya* pollen features pseudocolpi on both faces [29].

Supplementary Figure 4. Light microscopy (LM) and scanning electron microscopy (SEM) micrographs of Fagaceae and Juglandaceae pollen.

(a–c) *Quercus* sect. *Ilex* sp., EV, (c) exine sculpture detail. (d–f) *Quercus* sect. *Quercus* sp., PV, (f) exine sculpture detail. (g–i) Castaneoideae gen. indet., EV, (i) exine sculpture detail. (j–l) *Carya* sp. PV, (l) exine sculpture detail. (m–o) *Platycarya* sp., PV, (o) exine sculpture detail.

Abbreviations: equatorial view (EV), polar view (PV), distal view (DV), proximal view (PRV). Scale bars 10 µm (a, b, d, e, g, h, j, k, m, n), 1 µm (c, f, i, l, o).

Supplementary Figure 4. Light microscopy (LM) and scanning electron microscopy (SEM) micrographs of Fagaceae and Juglandaceae pollen.

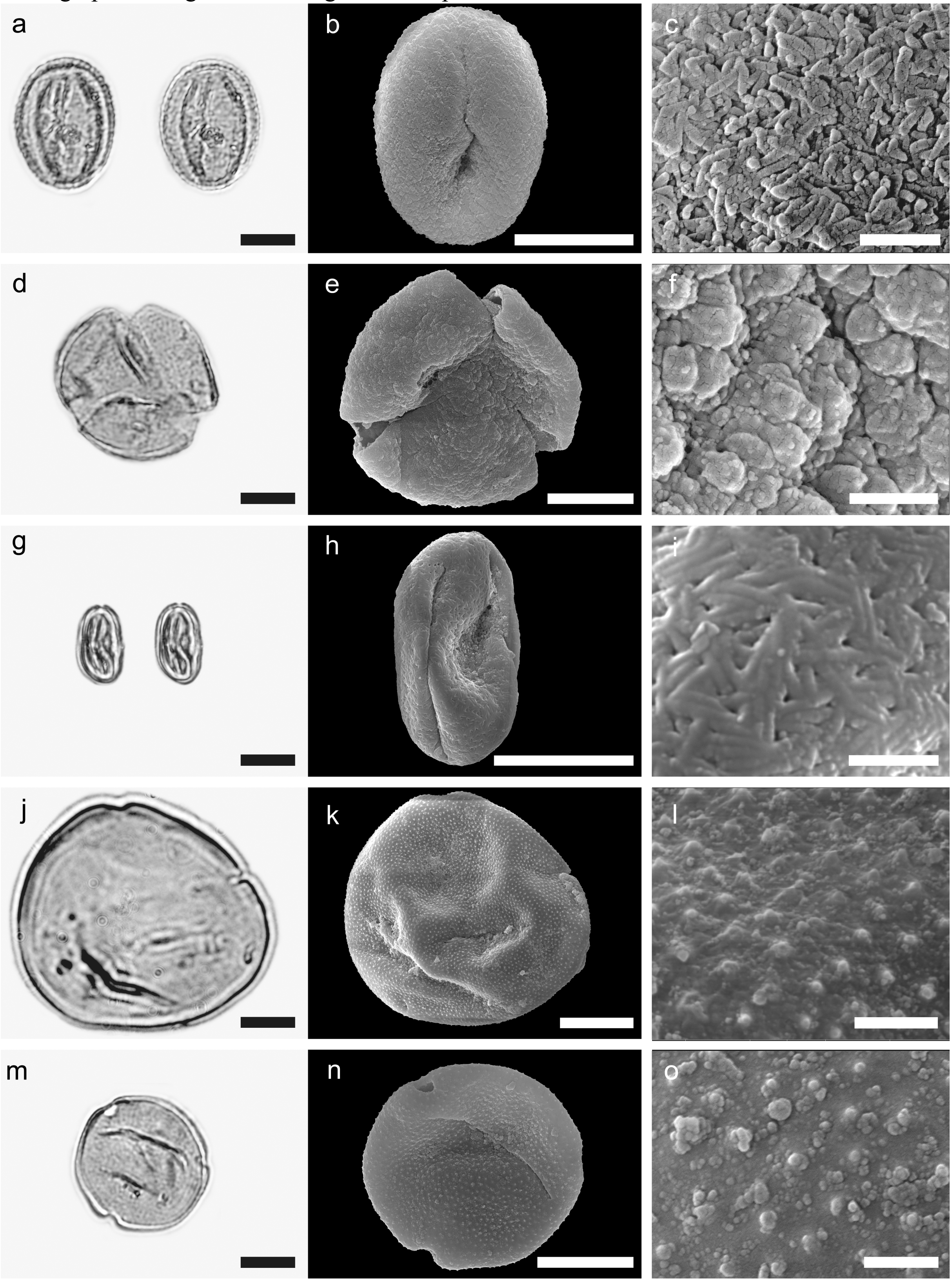

Subfamily Engelhardioideae Iljinsk.
Engelhardioideae gen. indet. (Fig. 4t; Supplementary Figs 5a–c)
Description: Pollen, monad, shape oblate, outline triangular in polar view, pollen size small, diameter 16–22 µm (LM); triporate, stephanoporate, pori sunken; sculpturing scabrate (LM), nanoechinate (SEM).

Remarks: Within Engelhardioideae pollen morphology is highly stenopalynous (triporate, nanoechinate sculpturing) [27]. The Engelhardioideae fossil record documents a diversity much higher than its extant three to four genera (*Alfaroa* Standl., *Alfaropsis* (Wall.) I.A.Iljinskaja, *Engelhardia* Lesch ex Blume, *Oreomunnea* Oerst.) and the presence of extinct linages during the Cenozoic [29].

Family Betulaceae Gray
Genus *Alnus* Mill.
Alnus sp. (Fig. 4u; Supplementary Figs 5d–f)
Description: Pollen, monad, shape oblate, outline polygonal in polar view, pollen size medium, diameter 25–40 µm (LM); tetra- to pentaporate, stephanoporate, pori annulate, adjacent pori connected by arci; sculpturing scabrate (LM), nanoechinate, two to three nanoechini grouped on microrugulae/ridges (SEM).

Remarks: Arci spanning between adjacent pores are characteristic of *Alnus* pollen [30].

Genus *Betula* L.
Betula sp. (Fig 4v; Supplementary Figs 5g–i)
Description: Pollen, monad, shape oblate, outline convex triagonal convex to circular in polar view, pollen size medium, diameter 25–40 µm (LM); triporate, pori annulate, annulus formed by sexine, vestibulum present; sculpturing scabrate (LM), nanoechinate, two to three nanoechini grouped on microrugulae/ridges (SEM).

Remarks: A distinct atrium is a characteristic feature of *Betula* [30].

Genus *Carpinus* L.
Carpinus sp. (Fig 4w; Supplementary Figs 5j–l)
Description: Pollen, monad, shape oblate, outline circular in polar view, pollen size medium,

diameter 35–50 µm (LM); tetra- to pentaporate, pori, weak aspis present; sculpturing scabrate (LM), nanoechinate, two to three nanoechini grouped on microrugulae (ridges) (SEM).

Remarks: Oblate pollen with weakly protruding apertures is characteristic of *Carpinus* [30].

Genus *Corylus* L.

Corylus sp. (Fig 4x; Supplementary Figs 5m–o)

Description: Pollen, monad, shape oblate, outline convex triangular in polar view, pollen size medium, diameter 25–40 µm (LM); triporate, pori annulate; sculpturing scabrate (LM), nanoechinate, two to three nanoechini grouped on microrugulae (ridges) (SEM).

Remarks: The absence of a distinct atrium and the convex triangular outline in polar view are characteristic of *Corylus* [30].

Supplementary Figure 5. Light microscopy (LM) and scanning electron microscopy (SEM) micrographs of Juglandaceae and Betulaceae pollen.

(a–c) Engelhardioideae gen. indet., PV, (c) exine sculpture detail. (d–f) *Alnus* sp., PV, (f) exine sculpture detail. (g–i) *Betula* sp., PV, (i) exine sculpture detail. (j–l) *Carpinus* sp., PV, (l) exine sculpture detail. (m–o) *Corylus* sp., PV, (o) exine sculpture detail.

Abbreviations: equatorial view (EV), polar view (PV), distal view (DV), proximal view (PRV). Scale bars 10 µm (a, b, d, e, g, h, j, k, m, n), 1 µm (c, f, i, l, o).

Supplementary Figure 5. Light microscopy (LM) and scanning electron microscopy (SEM) micrographs of Juglandaceae and Betulaceae pollen.

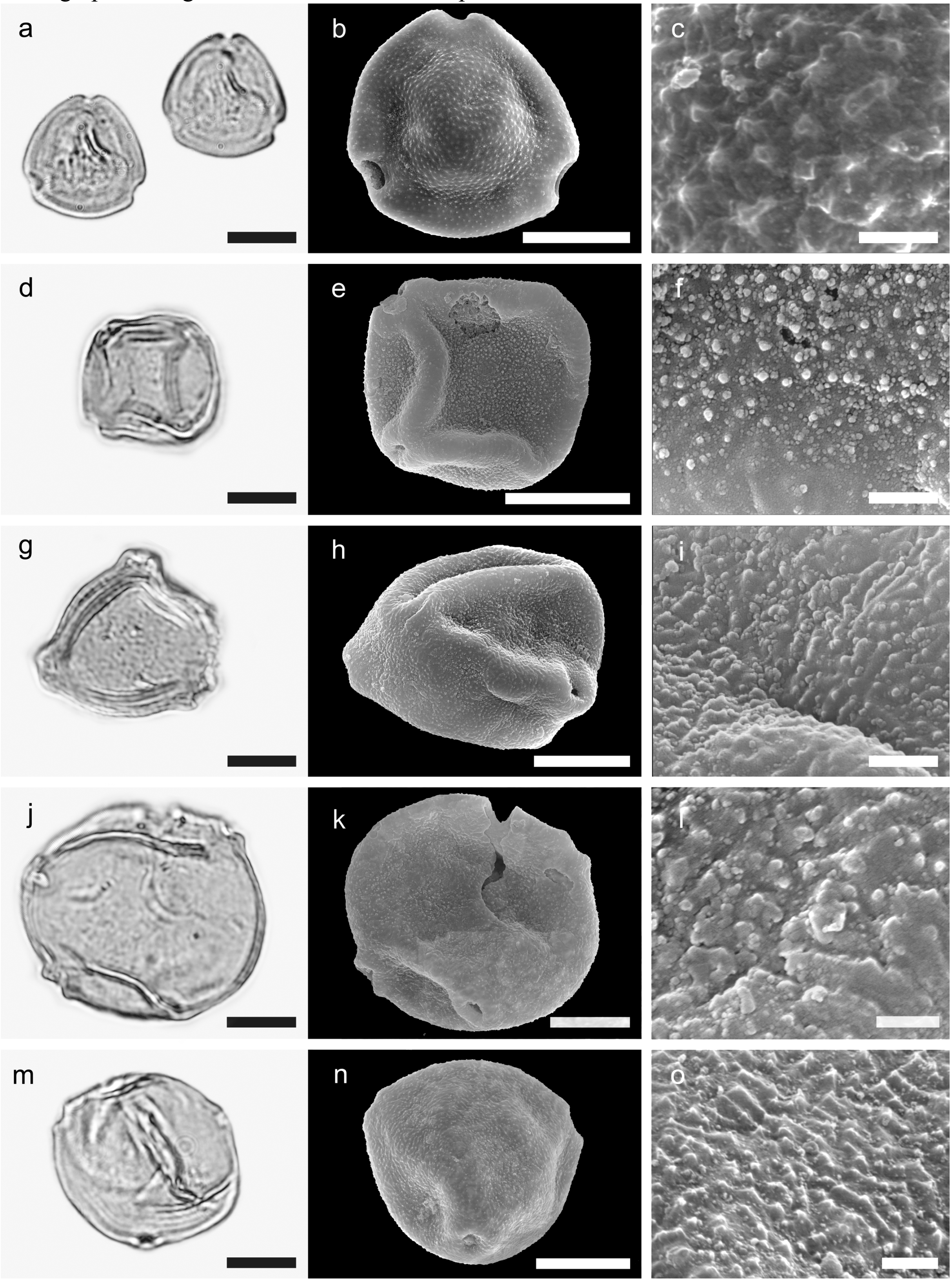

Order Malpighiales Juss. ex Bercht. & J.Presl

Family Salicaceae Mirb.

Genus *Salix* L.

Salix sp. (Fig. 4y; Supplementary Figs 6a–c)

Description: Pollen, monad, shape prolate to subprolate, outline elliptic in equatorial view, pollen size small, diameter 20–25 µm (LM); colpate; sculpturing reticulate (LM, SEM), muri wedge shaped (SEM).

Remarks: The figured specimen features wedge-shaped muri in SEM investigation; this is the most common muri type in *Salix* pollen [31].

Order Geraniales Juss. ex Bercht. & J.Presl

Family Geraniaceae Juss.

Genus *Geranium* L.

Geranium sp. (Figs 4z–aa; Supplementary Figs 6d–f)

Description: Pollen, monad, shape spheroidal, outline circular to weakly lobate in polar view, circular in equatorial view, pollen size large, diameter 65–80 µm (LM); tricolpate; sculpturing reticulate, clavate (LM, SEM), "heads" of clavae rugulate (SEM).

Remarks: The figured specimen corresponds (reticulum cristatum, clavae with rugulae and circular heads) to the *Geranium robertianum* group of the *Geranium molle* type of Stafford and Blackmore [32].

Order Sapindales Juss. ex Bercht. & J.Presl

Family Anacardiaceae R.Br.

Genus *Cotinus* Mill.

Cotinus sp. (Figs 5a–b; Supplementary Figs 6g–i)

Description: Pollen, monad, shape prolate, elliptic in equatorial view, pollen size medium, diameter 20–30 µm (LM), polar axis 25–35 µm long (LM); tricolporate, lalongate rhombic endoporus; sculpturing striate (LM), striatoreticulate (SEM), striae in colpus region more densely packed (SEM).

Remarks: The figured specimen displays typical characters (striatoreticulum, rhombic endoporus) of *Cotinus* [33].

Genus *Pistacia* L.

Pistacia sp. (Figs 5c–d; Supplementary Figs 6j–l)

Description: Pollen, monad, shape spheroidal, outline circular in polar view, pollen size medium, diameter 25–35 µm (LM); penta- to hepta pantoporate, pori sunken; sculpturing scabrate (LM), reticulate, nanoechinate, reticulum cristatum (SEM).

Remarks: The figured specimen displays a reticulum crested by relatively long nanoechini; similar pollen morphology was reported in *Pistacia atlantica* Desf. [34].

Family Sapindaceae Juss.

Genus *Acer* L.

Acer sp. 2 (Figs 5g–h; Supplementary Figs 6m–o)

Description: Pollen, monad, shape prolate, elliptic in equatorial view, lobate in polar view, pollen size medium, diameter 30–40 µm (LM), polar axis 35–45; tricolpate; sculpturing scabrate (LM), rugulate to striatoreticulate, fossulate, perforate (SEM).

Remarks: This type of exine sculpture is only found in the infrageneric sections *Rubra* and *Negundo* [35].

Supplementary Figure 6. Light microscopy (LM) and scanning electron microscopy (SEM) micrographs of Salicaceae, Geraniaceae, Anacardiaceae and Sapindaceae pollen.

(a–c) *Salix* sp., EV, (c) exine sculpture detail. (d–f) *Geranium* sp., PV, (f) exine sculpture detail. (g–i) *Cotinus* sp., EV, (i) exine sculpture detail. (j–l) *Pistacia* sp., PV, (l) exine sculpture detail. (m–o) *Acer* sp. 2, EV, (o) exine sculpture detail.

Abbreviations: equatorial view (EV), polar view (PV), distal view (DV), proximal view (PRV). Scale bars 10 µm (a, b, d, e, g, h, j, k, m, n), 1 µm (c, f, i, l, o).

Supplementary Figure 6. Light microscopy (LM) and scanning electron microscopy (SEM) micrographs of Salicaceae, Geraniaceae, Anacardiaceae, and Sapindaceae pollen.

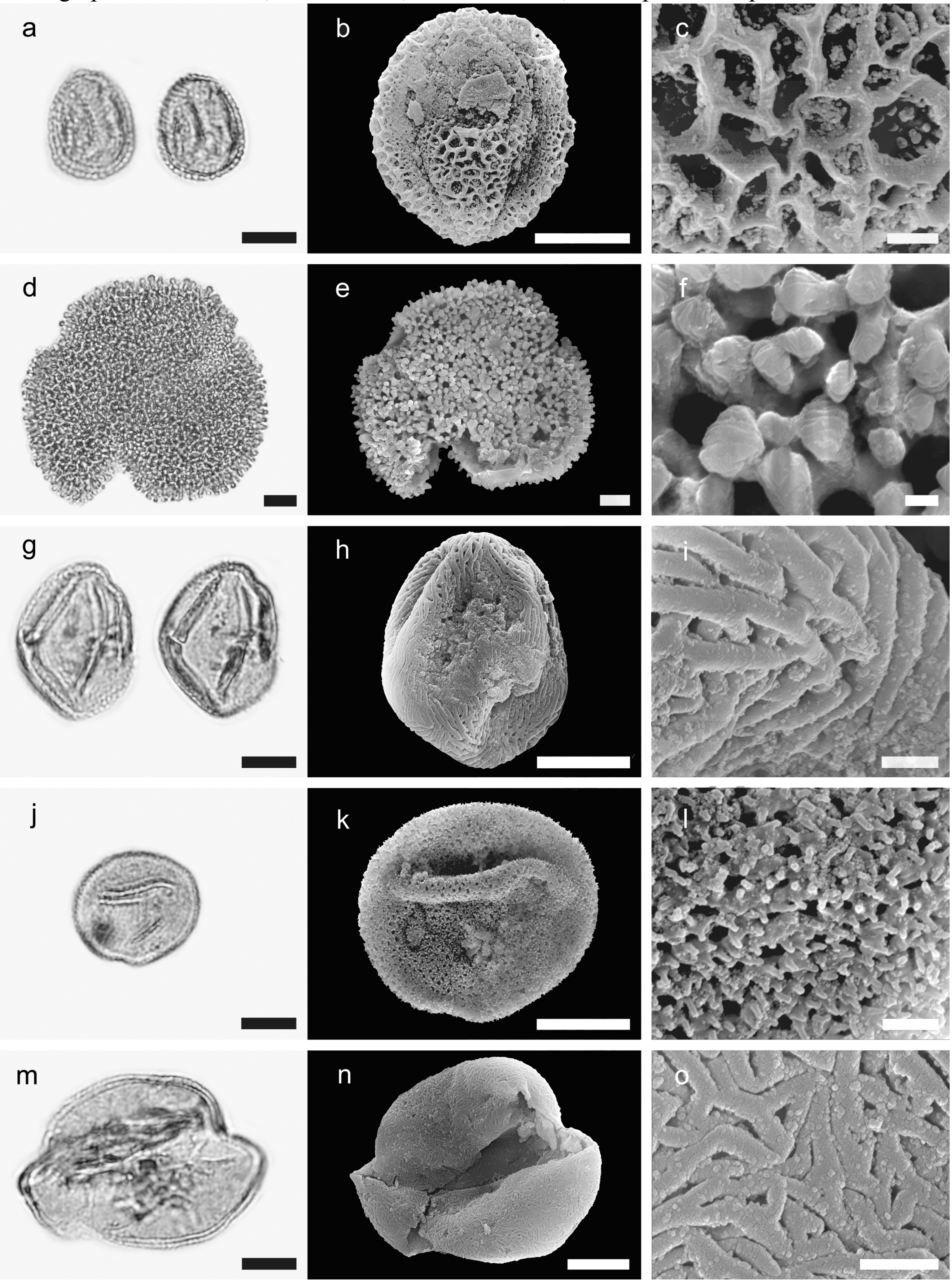

Acer sp. 1 (Figs 5e–f; Supplementary Figs 7a–c)
Description: Pollen, monad, shape prolate, lobate in polar view, pollen size medium, diameter 30–40 µm (LM); tricolpate; sculpturing striate (LM, SEM).

Remarks: Striate exine sculpturing is the most common type in *Acer* [35].

Order Malvales Juss. ex Bercht. & J.Presl
Family Malvaceae Juss.
Genus *Craigia* W.W.Sm. & W.E.Evans
Craigia sp. (Figs 5i–j; Supplementary Figs 7d–f)
Description: Pollen, monad, shape oblate, circular to convex triangular in polar view, pollen size medium, diameter 25–35 µm (LM); tricolporate, colpi short, endo aperture circular to elliptic; sculpturing microreticulate, heterobrochate (LM, SEM).

Remarks: The figured specimen corresponds by exine sculpturing and aperture configuration (horseshoe-shaped thickening in aperture area in polar view) to *Craigia* [36].

Order Caryophyllales Juss. ex Bercht. & J.Presl
Family Amaranthaceae Juss./Chenopodioideae Burnett
Amaranthaceae/Chenopodioideae gen. indet. sp.1 (Fig. 5k)
Description: Pollen, monad, shape spheroidal, outline circular, pollen size small, diameter 18–22 µm (LM); pantoporate, pori sunken, pori diameter 1–2 µm, 34–42 pori; sculpturing psilate (LM), perforate, nanoechinate (SEM).

Amaranthaceae/Chenopodioideae gen. indet. sp. 2 (Fig. 5l)
Description: Pollen, monad, shape spheroidal, outline circular, pollen size small, diameter 20–25 µm (LM); pantoporate, pori sunken, pori diameter 2–4 µm, 26–32 pori; sculpturing psilate (LM), perforate, nanoechinate (SEM).

Remarks: Amaranthaceae/Chenopodioideae gen. indet. sp.1 differs by smaller size and pore size from Amaranthaceae/Chenopodioideae gen. indet. sp.2. [15].

Family Caryophyllaceae Juss.
Caryophyllaceae gen. indet. (Figs 5m–n; Supplementary Figs 7g–i)
Description: Pollen, monad, shape spheroidal, outline circular, pollen size medium, diameter 35–50 µm (LM); pantoporate, pori sunken, pori diameter 5–7 µm, 12 pori, porus membrane with 8–11 microechini; sculpturing psilate (LM), perforate, nanoechinate (SEM).

Remarks: The figured specimen is poorly preserved, but shares morphological similarities (absence of distinct annulus, evenly distributed nanoechini and perforations, pore membrane covered by microechini) with the *Cerastium fontanum* type [37].

Order Cornales Link
Family Nyssaceae Dumortier
Genus *Nyssa* L.
Nyssa sp. (Figs 5o–p; Supplementary Figs 7j–l)
Description: Pollen, monad, shape spheroidal to subprolate, outline circular to convex triangular in polar view, circular in equatorial view, pollen size medium, polar axis 35–50 µm long (LM); tricolporate, endoporus circular; sculpturing scabrate (LM), rugulate, fossulate, perforate (SEM), perforations absent in colpus area.

Remarks: Pollen with similar smooth colpus area have been reported in extant *Nyssa sylvatica* Marshall and *N. sinensis* Oliv. [38].

Order Lamiales Bromhead
Family Oleaceae Hoffmanns. & Link
Genus *Fraxinus* L.
Fraxinus sp. (Figs 5q–r; Supplementary Figs 7m–o)
Description: Pollen, monad, shape prolate, circular in equatorial view, pollen size medium, equatorial diameter 20–30 µm (LM), polar axis 25–40 µm long (LM); tricolpate; sculpturing reticulate, heterobrochate (LM, SEM), reticulum crested by blunt nano echini and perpendicular ridges (SEM, Fig. 5r).

Remarks: The figured specimen corresponds (size, reticulum morphology, aperture) to the pollen of this genus [39, 40]. Morphologically similar pollen is produced by extant *Fraxinus excelsior* [41]. Fossil seeds of *Fraxinus* have been reported from the Vegora mine [42].

Supplementary Figure 7. Light microscopy (LM) and scanning electron microscopy (SEM) micrographs of Sapindaceae, Malvaceae, Caryophyllaceae, Nyssaceae, and Oleaceae pollen.

(a–c) *Acer* sp. 1, PV, oblique view, (c) exine sculpture detail. (d–f) *Craigia* sp., PV, (f) exine sculpture detail. (g–i) Caryophyllaceae gen. indet. sp., (i) exine sculpture detail. (j–l) *Nyssa* sp., PV, (l) exine sculpture detail. (m–o) *Fraxinus* sp., PV, oblique view, (o) exine sculpture detail.

Abbreviations: equatorial view (EV), polar view (PV), distal view (DV), proximal view (PRV). Scale bars 10 µm (a, b, d, e, g, h, j, k, m, n), 1 µm (c, f, i, l, o).

Supplementary Figure 7. Light microscopy (LM) and scanning electron microscopy (SEM) micrographs of Sapindaceae, Malvaceae, Caryophyllaceae, Nyssaceae, and Oleaceae pollen.

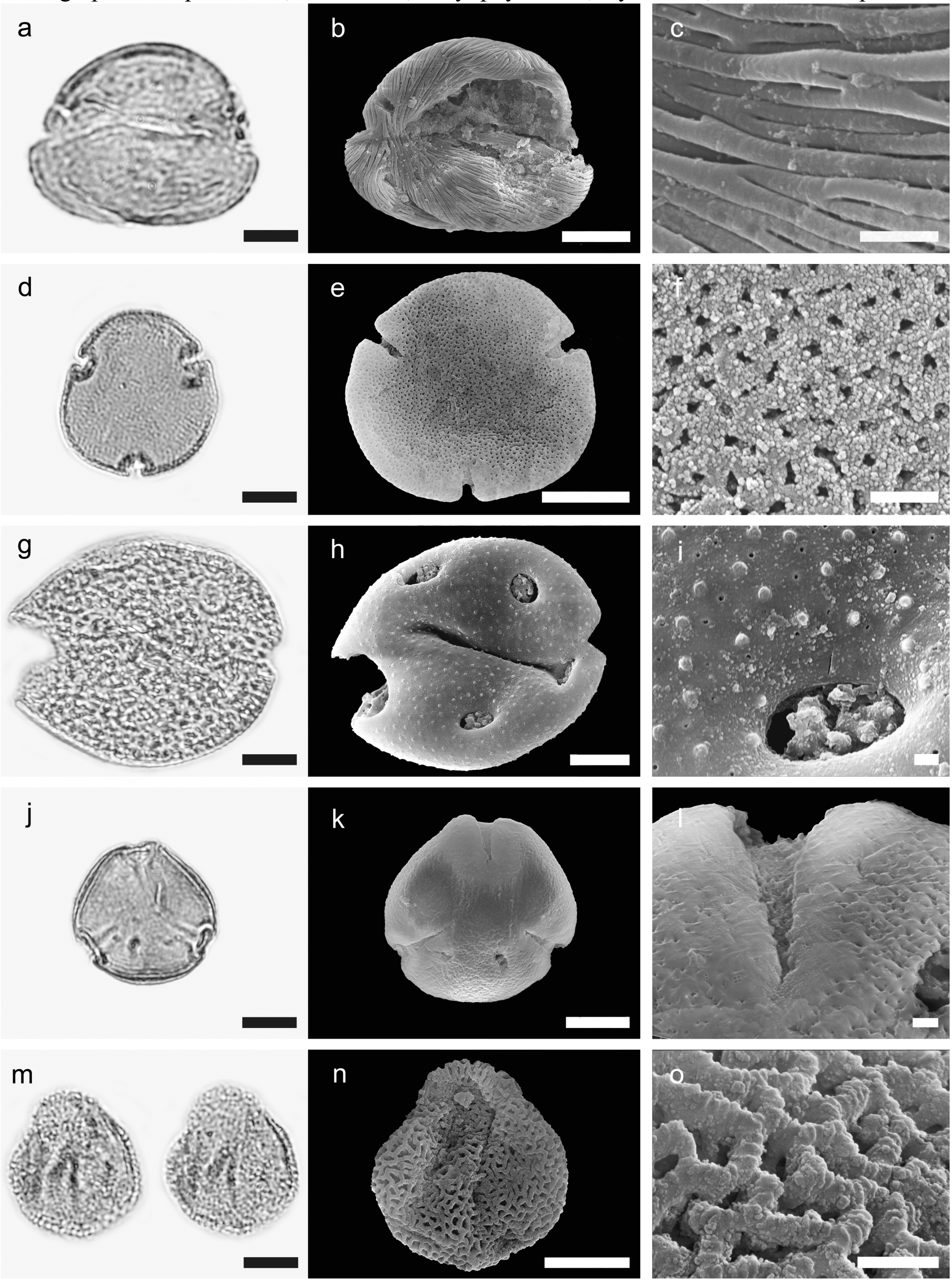

Genus *Olea* L.

Olea sp. (Figs 5s–t; Supplementary Figs 8a–c)

Description: Pollen, monad, shape spheroidal, circular in equatorial and polar view, pollen size small, equatorial diameter 18–25 µm (LM), polar axis 20–25 µm long (LM); tricolporate; sculpturing reticulate, heterobrochate (LM, SEM), reticulum crested by blunt nano echini (SEM, Fig. 5t).

Remarks: The figured specimen corresponds (reticulum morphology, aperture) to pollen of this genus [39, 40].

Order Asterales Link

Family Asteraceae Bercht. & J.Presl

Asteraceae gen. indet. sp. 1 (Fig. 5v; Supplementary Figs 8d–f)

Description: Pollen, monad, shape spheroidal, circular in equatorial, lobate in polar view, pollen size medium, diameter 25–30 µm (LM), polar axis 25–30 µm long (LM); tricolporate; sculpturing echinate, (LM), perforations extend to the upper third of echini, perforations circular (SEM).

Remarks: The poor preservation of Asteraceae gen. indet. sp. 1 and 2 prevents the determination of the aperture configuration, which is vital for the assignment to genus.

Asteraceae gen. indet. sp. 2 (Fig. 5w; Supplementary Figs 8g–i)

Description: Pollen, monad, shape spheroidal, circular in equatorial, lobate in polar view, pollen size medium, diameter 30–40 µm (LM), polar axis 30–40 µm long (LM); tricolporate; sculpturing echinate, (LM), perforations extend to the upper third of echini, perforations of irregular shape (SEM).

Subfamily Cichorioideae Chevall.

Cichorioideae gen. indet. (Fig. 5u; Supplementary Figs 8j–l)

Description: Pollen, monad, shape spheroidal, outline polygonal, pollen size medium, diameter 25–35 µm (LM), tricolporate; sculpturing echinate, lophate, (LM, SEM).

Remarks: Lophate pollen is characteristic of Cichorioideae [43]. The poor preservation prevents assignment to genus.

Order Dipsacales Juss. ex Bercht. & J.Presl

Family Caprifoliaceae Juss.

Genus *Succisa* Moench

Succisa sp. (Figs 5x–z; Supplementary Figs 8m–o)

Description: Pollen, monad, shape oblate to subspheroidal, outline circular to weakly lobate in polar view, pollen size large, diameter 60–75 µm (LM), tricolpate, colpus membrane echinate, echini 1.5–2.5 µm long; sculpturing echinate, microechinate, echini irregularly distributed over exine surface (LM, SEM), indistinct perforate (SEM).

Remarks: The morphology of the figured specimen corresponds (aperture without halo, echinate colpus membrane, irregularly distributed echini and microechini) to the *Succisa pratense* type of Clarke and Jones [44].

Supplementary Figure 8. Light microscopy (LM) and scanning electron microscopy (SEM) micrographs of Oleaceae, Asteraceae, and Caprifoliaceae pollen.

(a–c) *Olea* sp., EV, (c) exine sculpture detail. (d–f) Asteraceae gen. indet. sp. 1, PV, (f) exine sculpture and echini detail. (g–i) Asteraceae gen. indet. sp. 2, PV, (i) exine sculpture an echini detail. (j–l) Cichorioideae gen. indet., (l) exine sculpture detail. (m–o) *Succisa* sp., PV, (o) exine sculpture and aperture detail.

Abbreviations: equatorial view (EV), polar view (PV), distal view (DV), proximal view (PRV). Scale bars 10 µm (a, b, d, e, g, h, j, k, m, n), 1 µm (c, f, i, l, o).

Supplementary Figure 8. Light microscopy (LM) and scanning electron microscopy (SEM) micrographs of Oleaceae, Asteraceae, and Caprifoliaceae pollen.

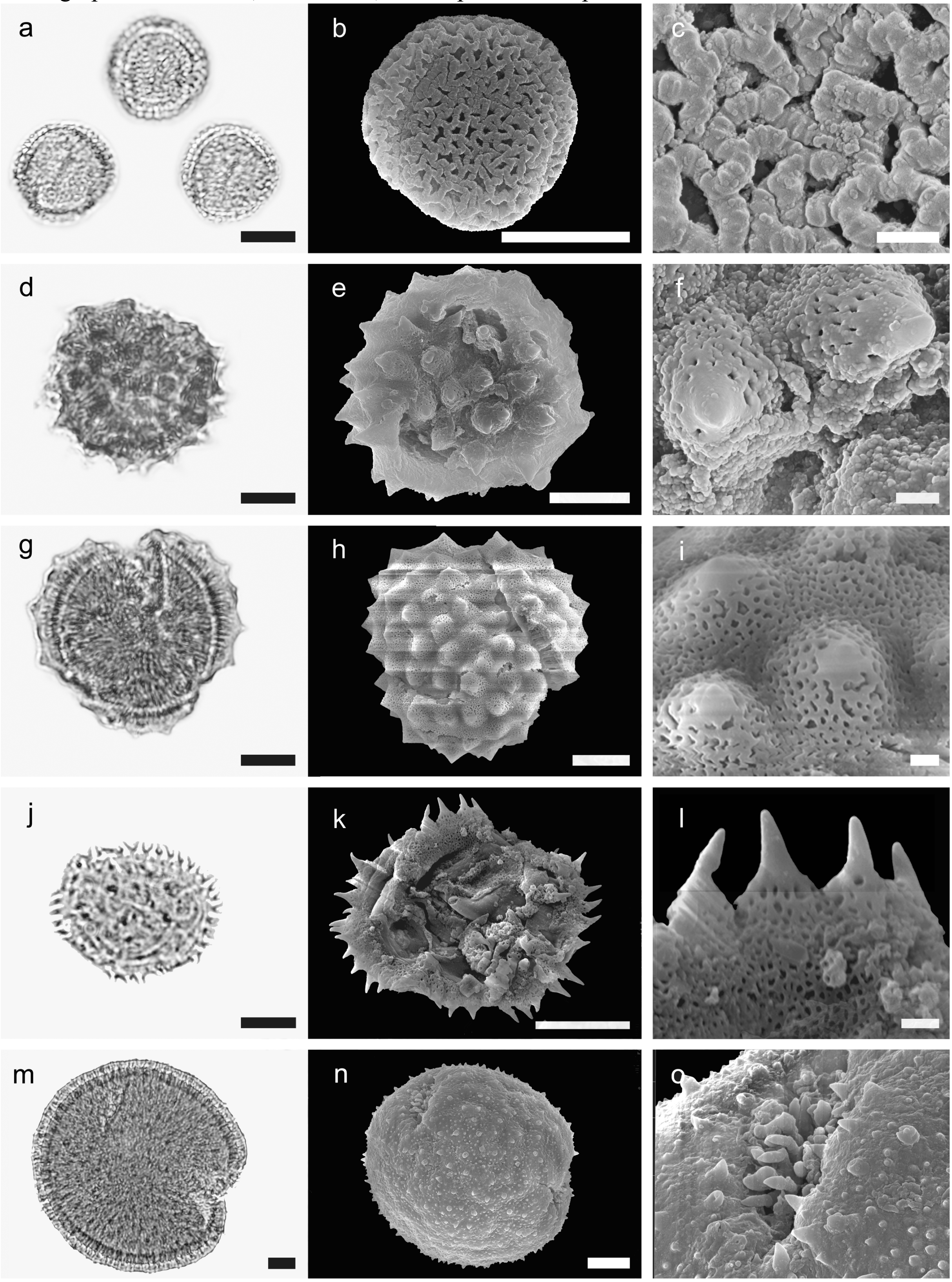

Supplementary Figure 9. Light microscopy (LM) and scanning electron microscopy (SEM) micrographs of Apiaceae pollen and pollen of uncertain affinity.

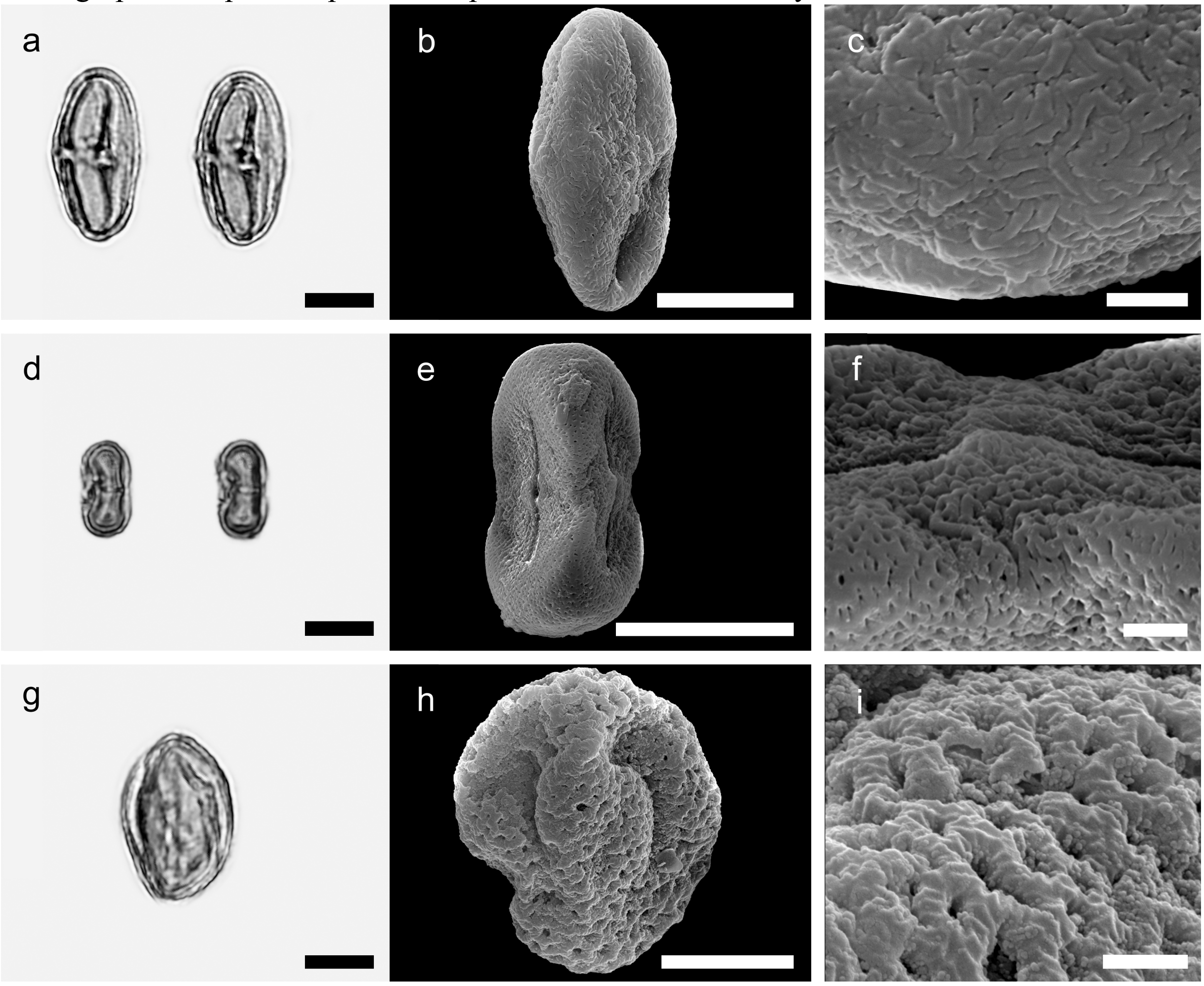

Order Apiales Nakai

Family Apiaceae Lindl.

Apiaceae sp.1 (Figs 5aa–bb; Supplementary Figs 9a–c)

Description: Pollen, monad, shape prolate, outline elliptic in equatorial view, pollen size small to medium, equatorial diameter 9–12 µm (LM), polar axis 22–30 µm (LM), tricolporate, colpus length 3/4 to 5/6 of polar axis, endoporus lalongate elliptic; sculpturing scabrate (LM), microrugulate, fossulate, perforate (SEM).

Remarks: Morphological similarities (colpus length, endoporus shape, exine sculpture) are with the *Sium latifolium* type of Punt [45].

Apiaceae sp. 2 (Figs 5cc–dd; Supplementary Figs 9d–f)

Description: Pollen, monad, shape prolate, outline bone-shaped in equatorial view, pollen size small, equatorial diameter 6–8 µm (LM), polar axis 16–20 µm (LM), tricolporate, colpus length 1/3 to 1/2 of polar axis, endoporus lalongate elliptic; sculpturing scabrate (LM), perforate (SEM).

Remarks: Morphological similarities (colpus length, endoporus shape, exine sculpture) are with the *Trinia glauca* type and the *Sison amomum* type of Punt [45].

Incertae sedis

Monocotyledonae indet. (Figs 4d–e; Supplementary Figs 3a–c)

Description: Pollen, monad, shape oblate, outline elliptic in polar view, monad size small, diameter 18–25 µm (LM); sulcate; sculpturing reticulate (LM, SEM).

Remarks: The figured specimen is not complete; therefore unambiguous determination is not possible. Sulcate, reticulate pollen is commonly present in e.g. Arecaceae [46] or Liliaceae [47].

Dicotyledonae fam. et gen. indet. (Figs 4ee–ff; Supplementary Figs 8g–i)

Description: Pollen, monad, shape prolate, outline elliptic in equatorial view, pollen size small to medium, diameter 20–30 µm (LM), tricolporate; sculpturing scabrate (LM), microreticulate, reticulum with perpendicular ridges (SEM).

Supplementary Figure 9. Light microscopy (LM) and scanning electron microscopy (SEM) micrographs of Apiaceae pollen and pollen of uncertain affinity.

(a–c) Apiaceae sp. 1, EV, (c) exine sculpture detail. (d–f) Apiaceae sp. 2, EV, (f) exine sculpture and echini detail. (g–i) Dicotyledonae fam. et gen. indet. sp., EV, (i) exine sculpture an echini detail.

Abbreviations: equatorial view (EV).Scale bars 10 µm (a, b, d, e, g, h), 1 µm (c, f, i).

References:

1. The Angiosperm Phylogeny Group (APG). 2016 An update of the angiosperm phylogeny group classification for the orders and families of flowering plants APG IV. *Bot. J. Linn. Soc.* **181**, 1–20.
2. Worobiec E. 2014 Fossil zygospores of Zygnemataceae and other microremains of freshwater algae from two Miocene palaeosinkholes in the Opole region, SW Poland. *Acta Palaeobot.* **54**, 113–157.
3. Krutzsch W, Pacltová B. 1990 Die Phytoplankton-Mikroflora aus den pliozänen Süsswasserablagerungen des Cheb-Beckens (Westböhmen, CSFR). *Acta Univ. Carolinae - Geologica* **34**, 345–420.
4. Stafford PJ. 2003 The Northwest European pollen flora, 57: Osmundaceae. *Rev. Palaeobot. Palynol.* **123**, 1–7.
5. Tyron AF, Lugardon B. 1991 *Spores of the Pteridophyta*. NewYork, Berlin, Heidelberg: Springer.
6. Krutzsch W. 1967 *Atlas der mittel- und jungtertiären dispersen Sporen- und Pollen- sowie der Mikroplanktonformen des nördlichen Mitteleuropas IV–V*. Jena: VEB Gustav Fischer Verlag.
7. Stuchlik L, Ziembińska-Tworzydło M, Kohlman-Adamska A, Grabowska I, Ważyńska H, Słodkowska B, Sadowska A. 2001 *Atlas of pollen and spores of the Polish Neogene - Spores*. Krakow: W. Szafer Institute of Botany, Polish Academy of Sciences.
8. Morbelli MA, Ponce MM. 1997 Palynological study of *Cheilanthes* and *Astrolepis* (Pteridaceae) species from Northwestern Argentina. *Am. Fern J.* **87**, 53–65.
9. Salimpor F, Nazi M, Mazooji A. 2011 Spore morphology of Pteridaceae in Iran. *Austr. J. Basic Appl. Sci.* **5**, 1154–1156.
10. Krutzsch W. 1971 *Atlas der mittel- und jungtertiären dispersen Sporen- und Pollen- sowie der Mikroplanktonformen des nördlichen Mitteleuropas VII*. Jena: VEB Gustav Fischer Verlag.
11. Stuchlik L, Ziembińska-Tworzydło M, Kohlman-Adamska A, Grabowska I, Ważyńska H, Słodkowska B, Sadowska A. 2001 *Atlas of pollen and spores of the Polish Neogene - Gymnosperms*. Krakow: W. Szafer Institute of Botany, Polish Academy of Sciences.
12. Bouchal JM, Denk T. 2020 Low taxonomic resolution of papillate Cupressaceae pollen (former Taxodiaceae) impairs their applicability for palaeo-habitat reconstruction. *Grana* **59**, 71–93.

13. Kedves M. 1985 LM, TEM and SEM investigations on recent inaperturate Gymnospermatophyta pollen grains. *Acta Biol. Szeged*. **31**, 129–146.
14. Miyoshi N, Fujiki T, Kimura H. 2011 *Pollen flora of Japan*. Sapporo: Hokkaido University Press.
15. Beug HJ. 2004 *Leitfaden der Pollenbestimmung für Mitteleuropa und angrenzende Gebiete*. München: Dr. Friedrich Pfeil Verlag.
16. Liu YS, Basinger JF. 2000 Fossil *Cathaya* (Pinaceae) pollen from the Canadian high arctic. *Int. J. Plant Sci.* **16**, 829–847.
17. Sivak J. 1976 Nouvelles espèces du genre *Cathaya* d'après leurs grains de pollen dans le Tertiaire du Sud de la France. *Pollen et Spores* **18**, 243–288.
18. Fujiki T, Inoue Y, Yasuda Y. 2003 Pollen morphology of *Cedrus* (Pinaceae). *Jap. J. Palyn*. **49**, 21–24.
19. Halbritter H, Ulrich S, Grímsson F, Weber M, Zetter R, Hesse M, Buchner R, Svojtka M, Frosch-Radivo A. 2018 *Illustrated Pollen Terminology, 2nd ed*. Cham (Switzerland): Springer Nature.
20. Sivak J. 1978 Histoire de genre *Tsuga* en Europe d'après l'étude des grains de pollen actuels et fossiles. *Paléobiol. Continent.* **9**, 1–226.
21. Punt W. 1975 The Northwest European pollen flora, 5: Sparganiaceae and Typhaceae. *Rev. Palaeobot. Palynol.* **19**, 75–88.
22. Page JS. 1978 A scanning electron microscope survey of grass pollen. *Kew Bull*. **32**, 313–319.
23. Lu L, Wen J, Chen Z. 2012 A combined morphological and molecular phylogenetic analysis of *Parthenocissus* (Vitaceae) and taxonomic implications. *Bot. J. Linn Soc.* **168**, 43–63.
24. Stafford PJ. 1995 The Northwest European pollen flora, 53: Ulmaceae. *Rev. Palaeobot. Palynol.* **88**, 25–46.
25. Denk T, Grimm GW. 2009 Significance of pollen characteristics for infrageneric classification and phylogeny in *Quercus* (Fagaceae). *Int. J. Plant Sci*. **170**, 929–940.
26. Praglowski J. 1982 Fagaceae L. - Fagoideae. In: Praglowski J, ed. *World pollen and spore flora 11.* Stockholm: Almquist & Wiksell Periodical Company.
27. Praglowski J. 1984 Fagaceae Dumort. - Castaneae Oerst. In: Praglowski J, ed. *World pollen and spore flora 13*. Stockholm: Almquist & Wiksell Periodical Company.
28. Stone DE, Broome CR. 1975 Juglandaceae A.Rich. ex Kunth. In: Nilsson S, ed. *World pollen and spore flora 4.* Stockholm: Almquist & Wiksell Periodical Company.

29. Kvaček Z. 2007 Do extant nearest relatives of thermophile European Cenozoic plant elements reliably reflect climatic signal? *Palaeogeogr. Palaeoclimat. Palaeoecol.* **253**, 32–40.
30. Blackmore S, Steinmann JAJ, Hoen PP, Punt W. 2003 The northwest European pollen flora, 65: Betulaceae and Corylaceae. *Rev. Palaeobot. Palynol.* **123**, 71–98.
31. Sohma K. 1993 Pollen diversity in *Salix* (Salicaceae). *Sci. Rep. Tohoku Univ. Ser.* **4**, 77–178.
32. Stafford PJ, Blackmore S. 1991 The Northwest European pollen flora, 46: Geraniaceae. *Rev. Palaeobot. Palynol.* **69**, 49–78.
33. Halbritter H. 2015 *Cotinus coggygria*. In: *PalDat - A palynological database.* https://www.paldat.org/pub/Cotinus_coggygria/300038; accessed 2019-10-03
34. Belhadj S, Derridj A, Civeyrel L, Gers C, Aigouy T, Otto T, Gauquelin T. 2007 Pollen morphology and fertility of wild Atlas pistachio (*Pistacia atlantica* Desf., Anacardiaceae). *Grana* **46**, 148–156.
35. Biesboer DD. 1975 Pollen morphology of the Aceraceae. *Grana* **15**, 19–27.
36. Zetter R, Weber M, Hesse M, Pingen M. 2002 Pollen, pollenkitt, and orbicules in *Craigia bronnii* flower buds (Tilioideae, Malvaceae) from the Miocene of Hambach, Germany. *Int. J. Plant Sci.* **163**: 1067–1071.
37. Punt W., Hoen PP. 1995 The northwest European pollen flora, 56: Caryophyllaceae. *Rev. Palaeobot. Palynol*. **88**, 83–272.
38. Göschl W. 2008 Beiträge zur Pollenmorphologie ausgewählter rezenter und fossiler Vertreter der Davidiaceae und Nyssaceae. Master's Thesis, University of Vienna, Vienna, Austria.
39. Punt W, Bos JAA, Hoen PP. 1991 The northwest European pollen flora, 45: Oleaceae. *Rev. Palaeobot. Palynol*. **69**, 23–47.
40. Guo S, Fujiki T, Miyoshi N. 1994 Pollenmorphology by means of scanning electron microscope:13. Oleaceae (Angiospermae). *Japan. J. Palynol.* **40**, 99–112.
41. Diethart B. 2016 *Fraxinus excelsior.* In: *PalDat - A palynological database.* https://www.paldat.org/pub/Fraxinus_excelsior/301215; accessed 2019-10-04
42. Kvaček Z, Velitzelos D, Velitzelos E. 2002 *Late Miocene Flora of Vegora Macedonia N. Greece.* Athens: Koralis.
43. Blackmore S. 1984 The northwest European pollen flora, 32: Compositae — Lactuceae. *Rev. Palaeobot. Palynol.* **42**, 45–85.

44. Clarke GCS, Jones MR. 1981 The northwest European pollen flora, 21: Dipsacaceae. *Rev. Palaeobot. Palynol.* **33**, 1–25.

45. Punt W. 1984 The northwest European pollen flora, 37: Umbelliferae. *Rev. Palaeobot. Palynol.* **42**, 155–364.

46. Dransfield J, Uhl NW, Asmussen CB, Baker WJ, Harley MM, Lewis CE. 2008 *Genera Palmarum. The evolution and classification of palms*. Kew: Royal Botanic Gardens.

47. Erdtman G. 1965 *Pollen morphology and plant taxonomy – Angiosperms (an introduction to Palynology I)*. New York, London: Hafner Publishing Company.
