# Supplementary figures and images for: Messinian vegetation and climate of the intermontane Florina–Ptolemais–Servia Basin, NW Greece inferred from palaeobotanical data: how well do plant fossils reflect past environments?
Source: R Soc Open Sci. 2020 May 27;7(5):192067. doi: 10.1098/rsos.192067 (PMC7277258; doi:10.1098/rsos.192067)

MAT (°C)

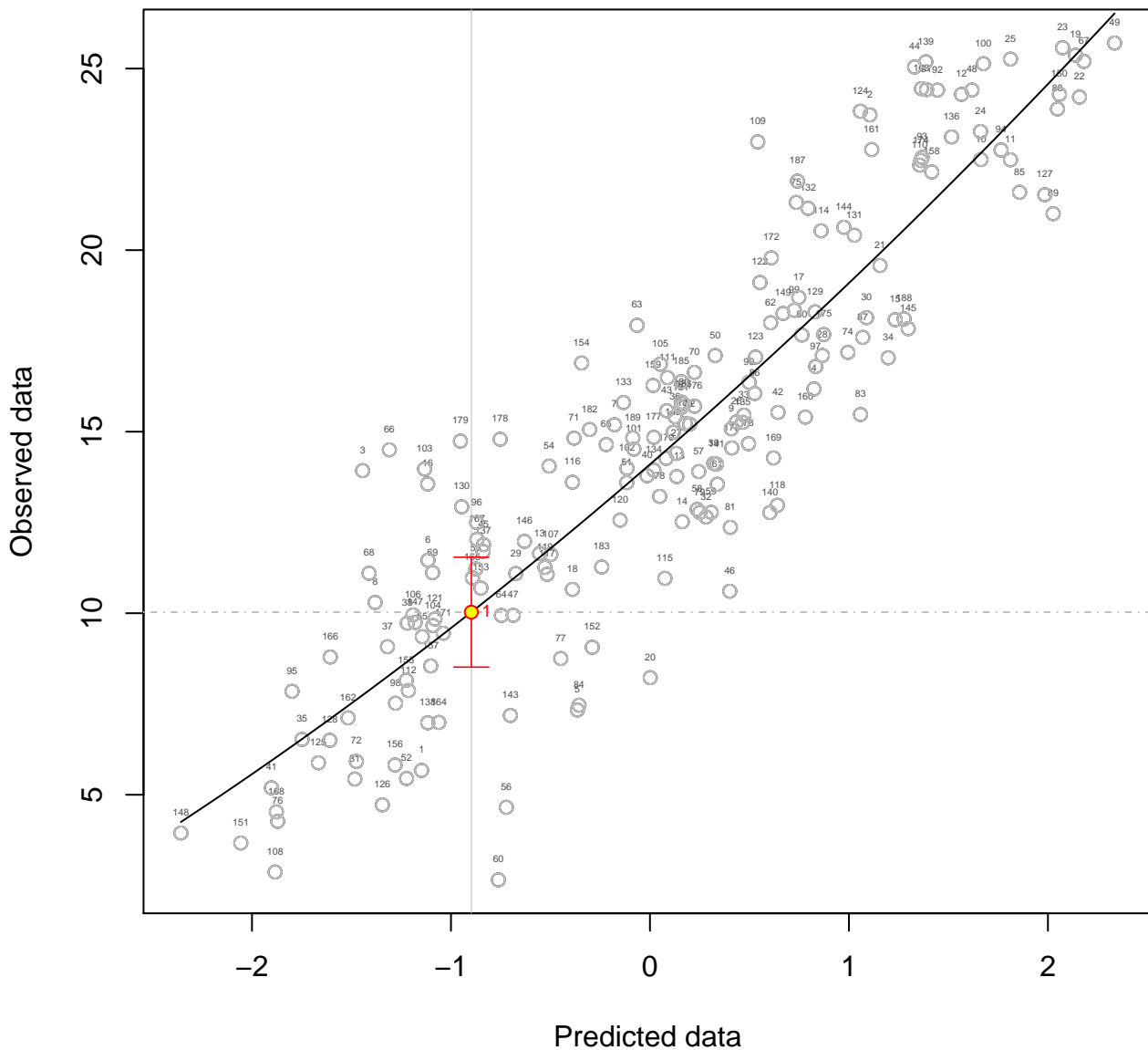

WMMT (°C)

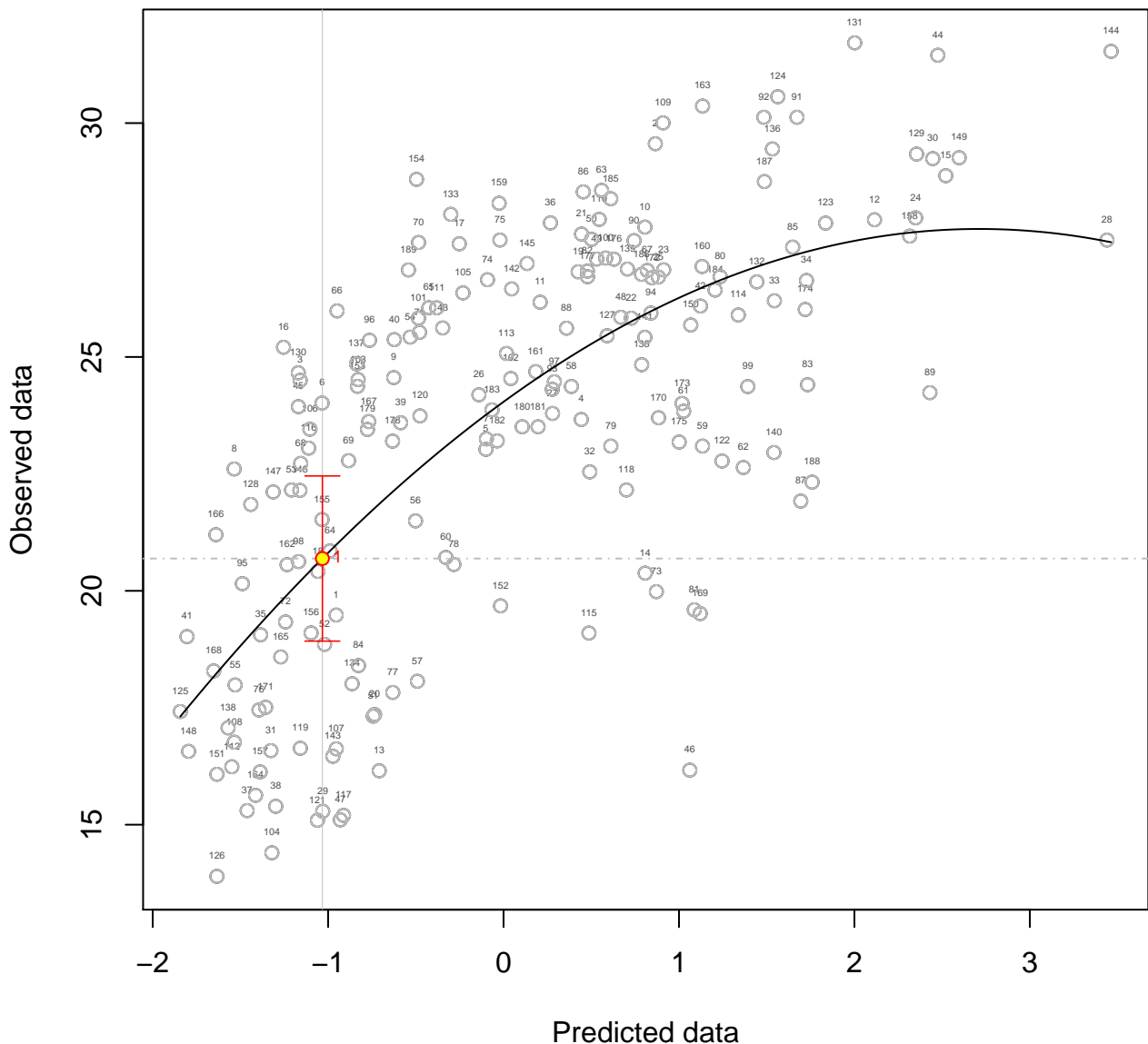

CMMT (°C)

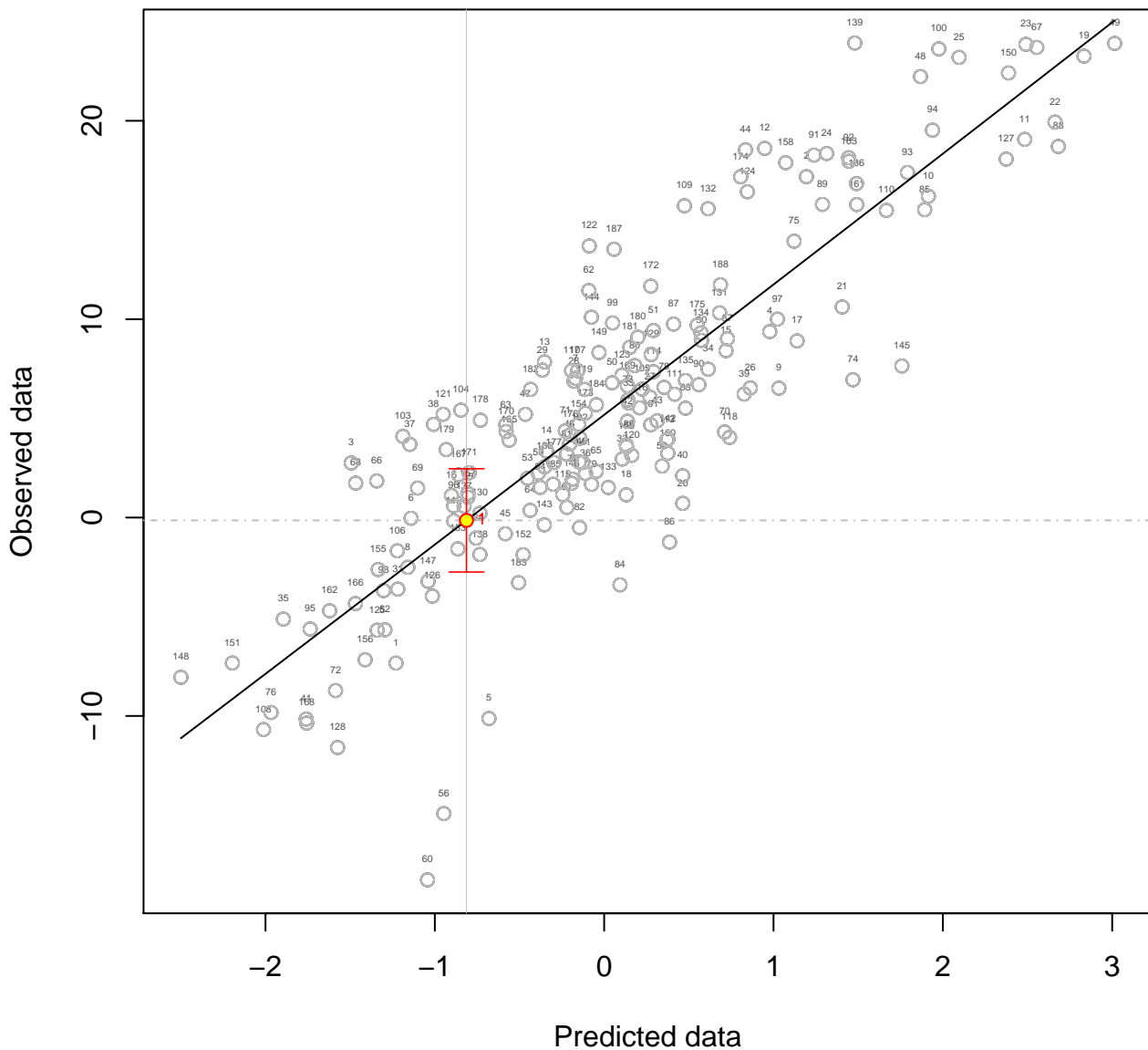

GROWSEAS (months)

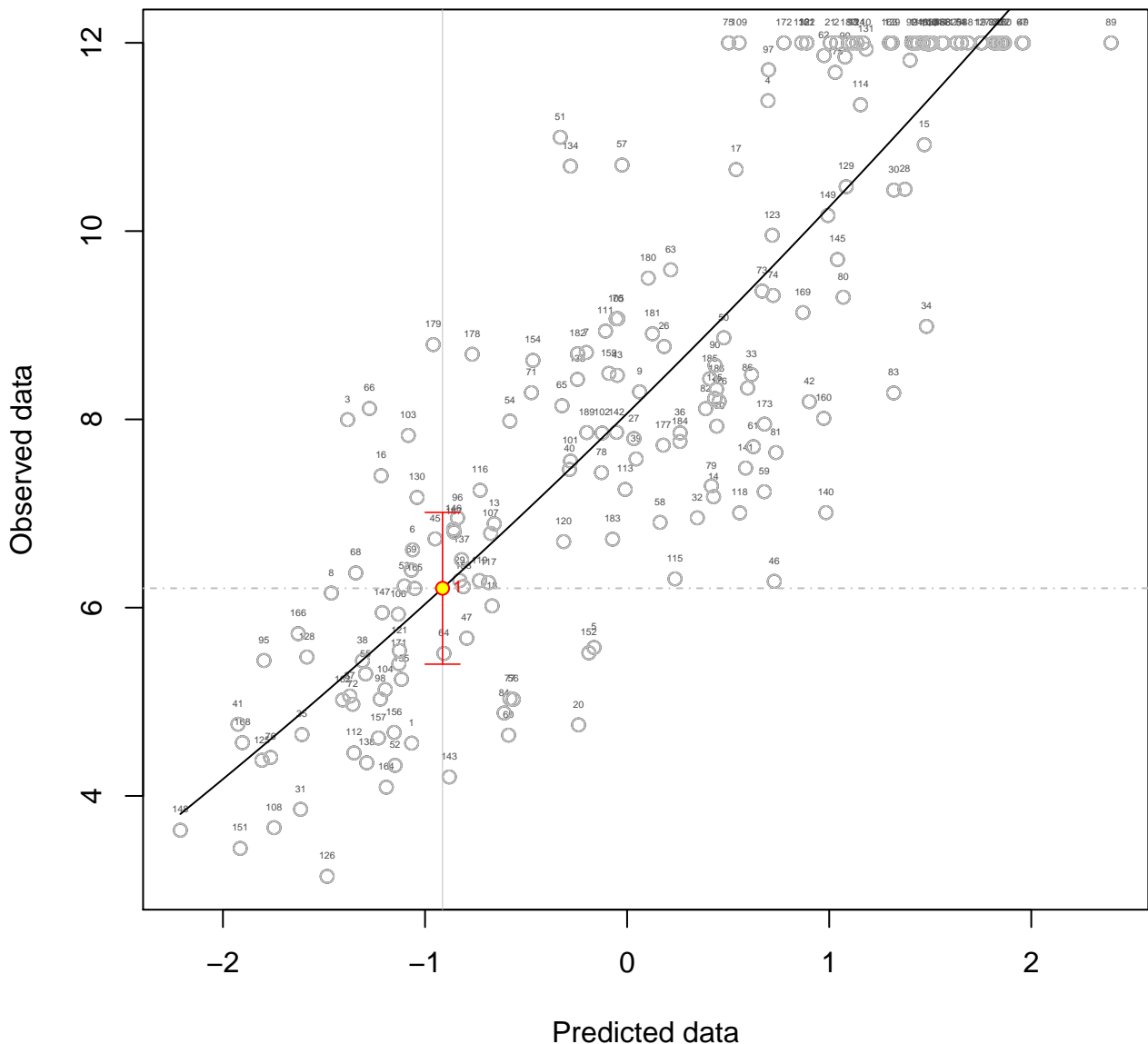

GSP (cm)

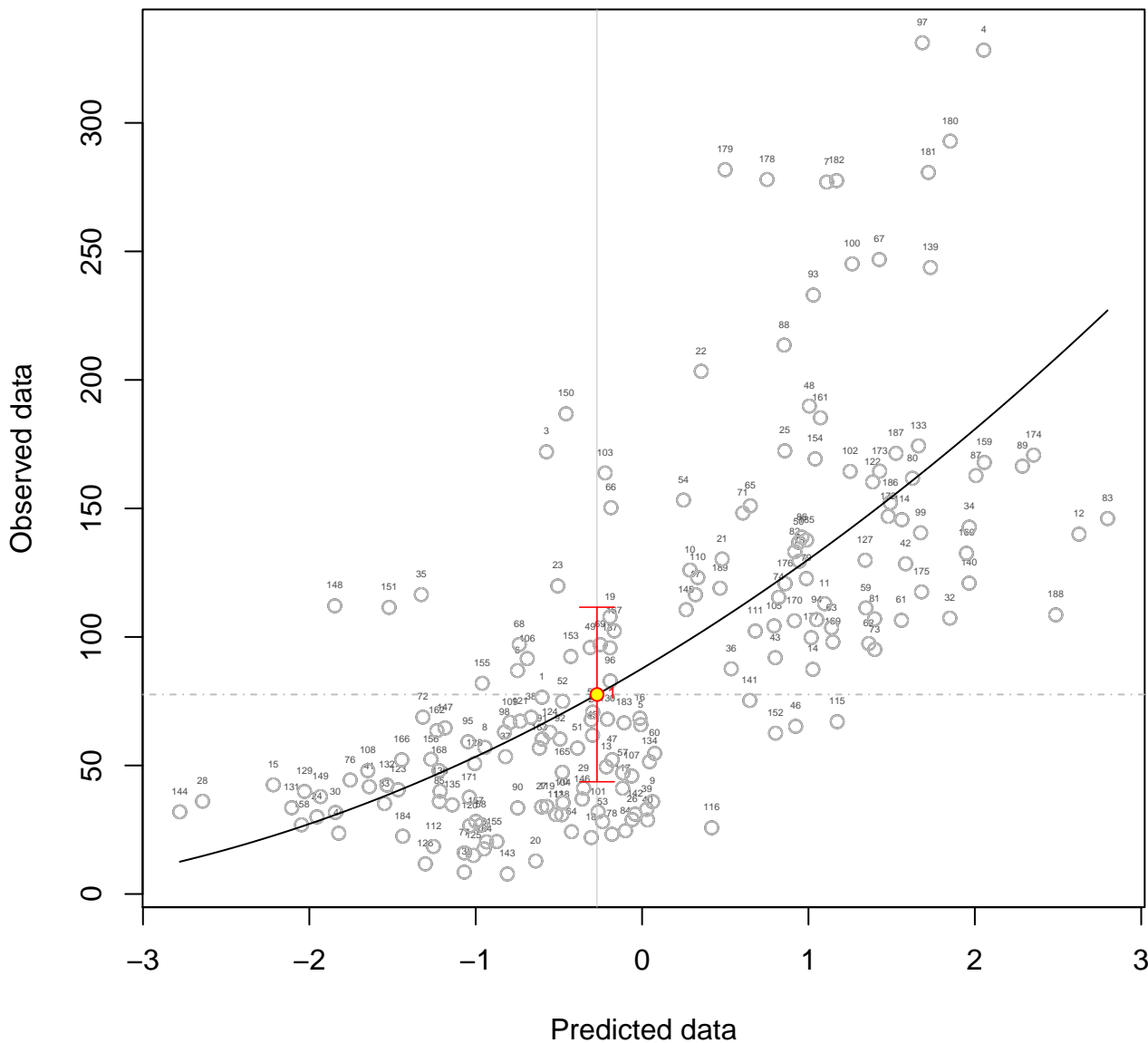

MMGSP (cm)

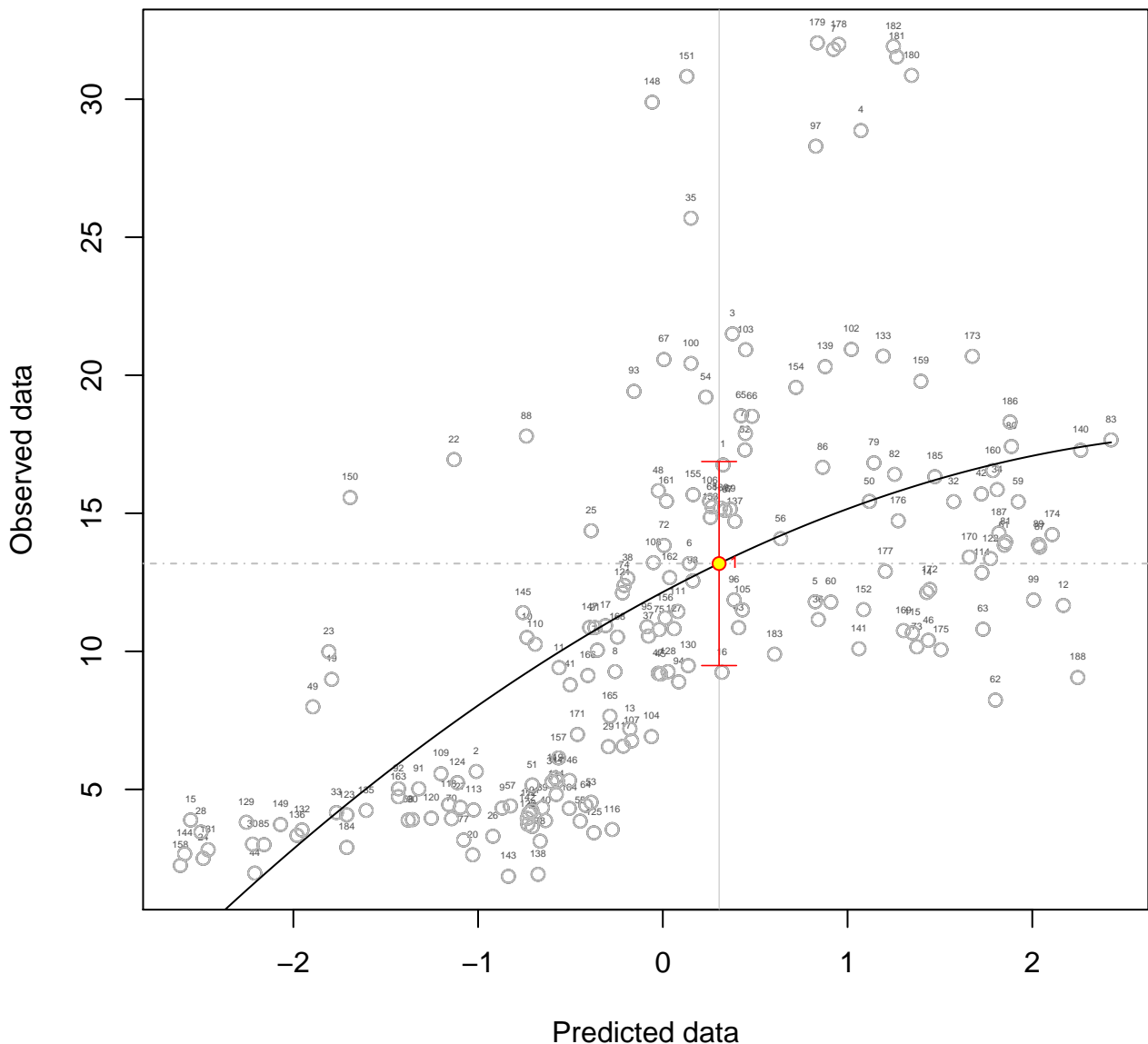

X3.WET (cm)

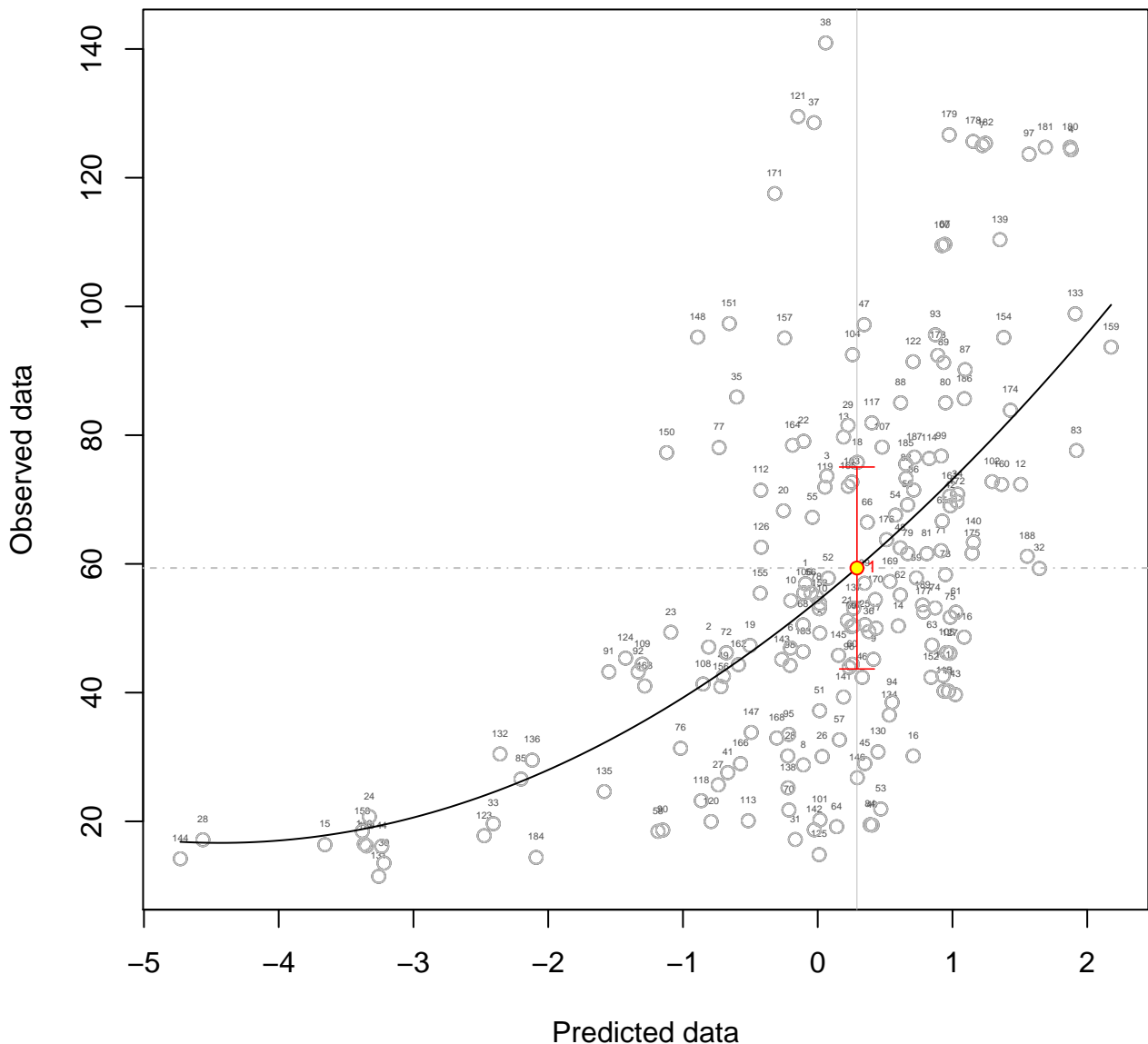

X3.DRY (cm)

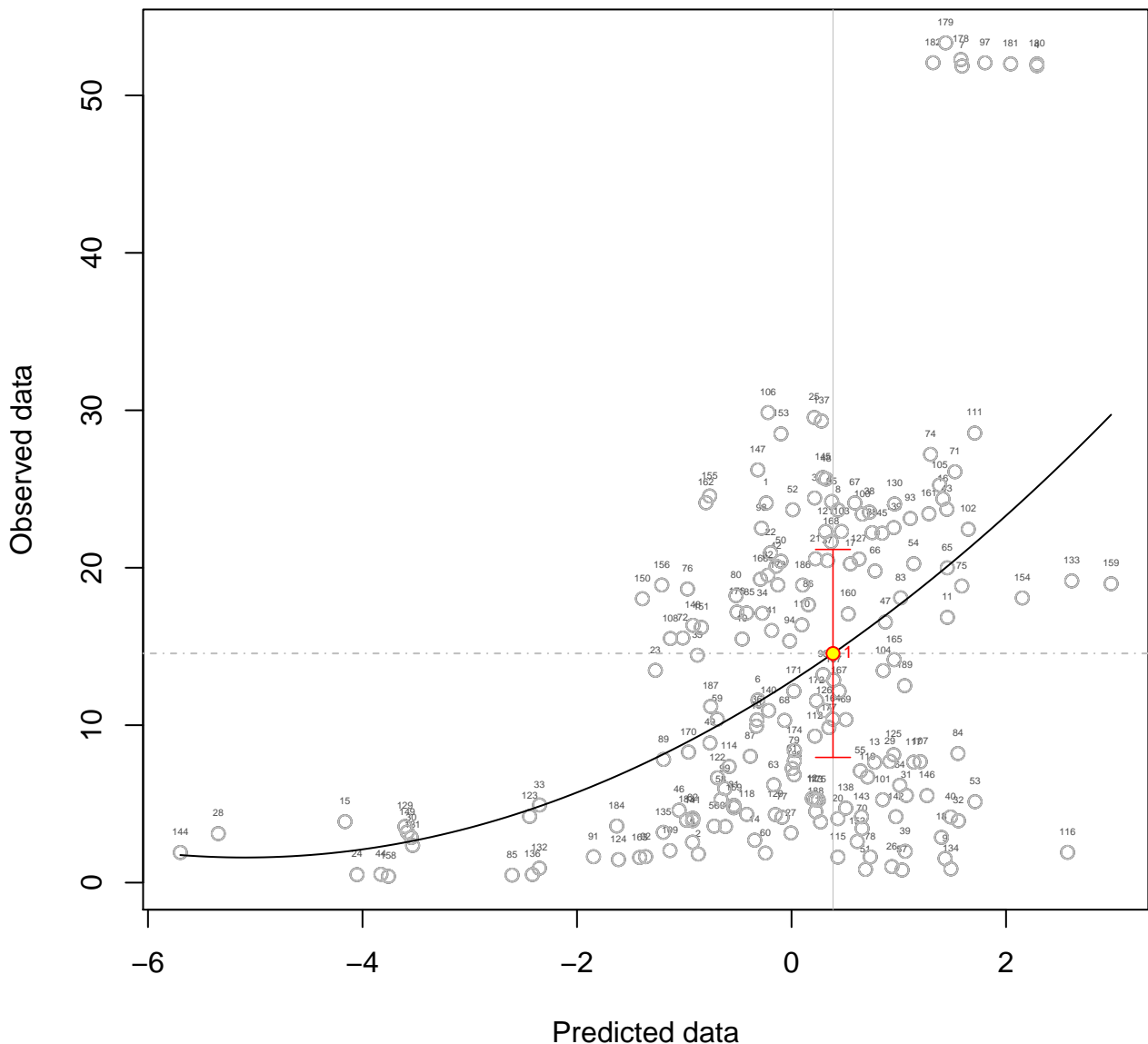

RH (%)

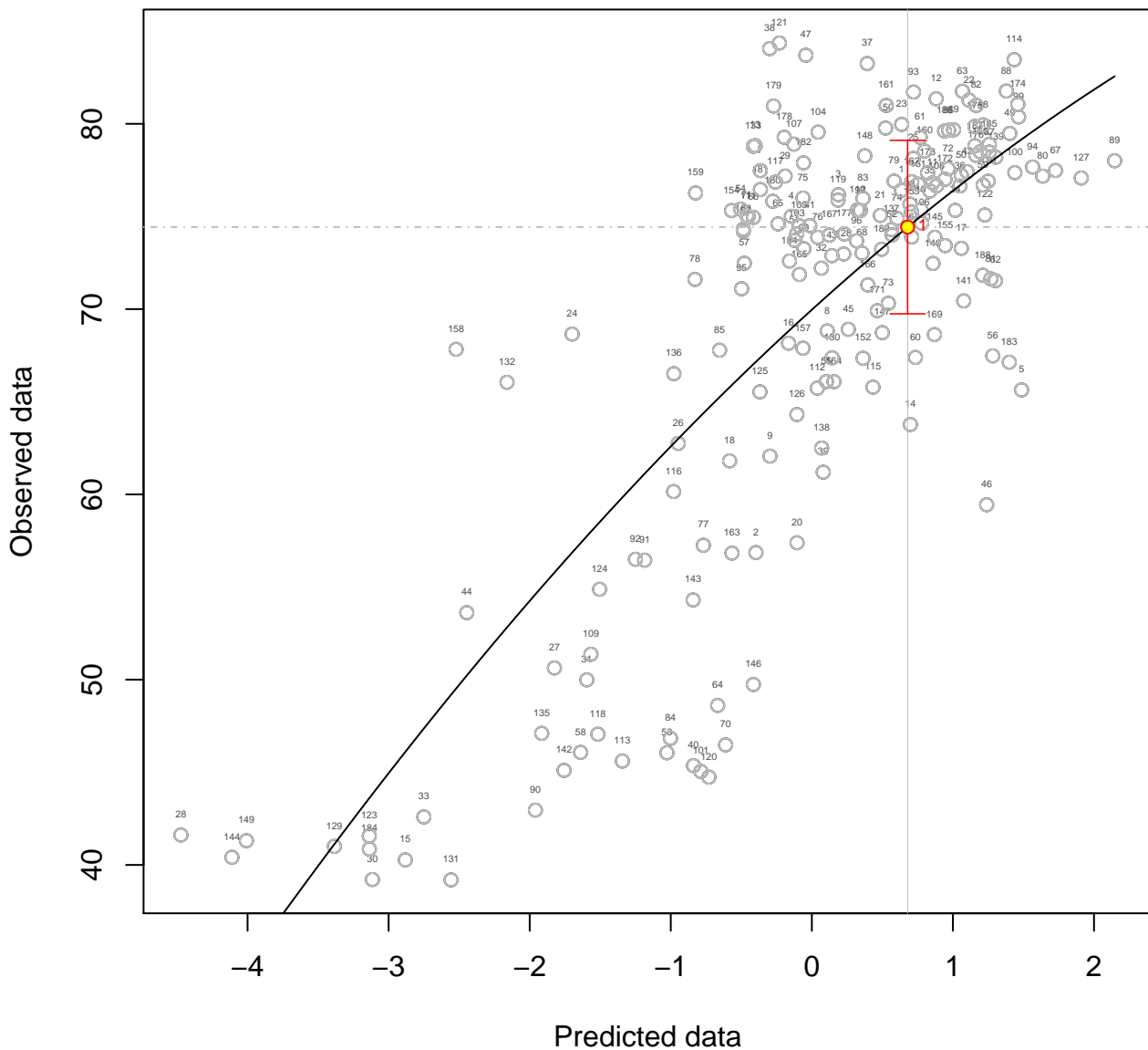

SH (g/Kg)

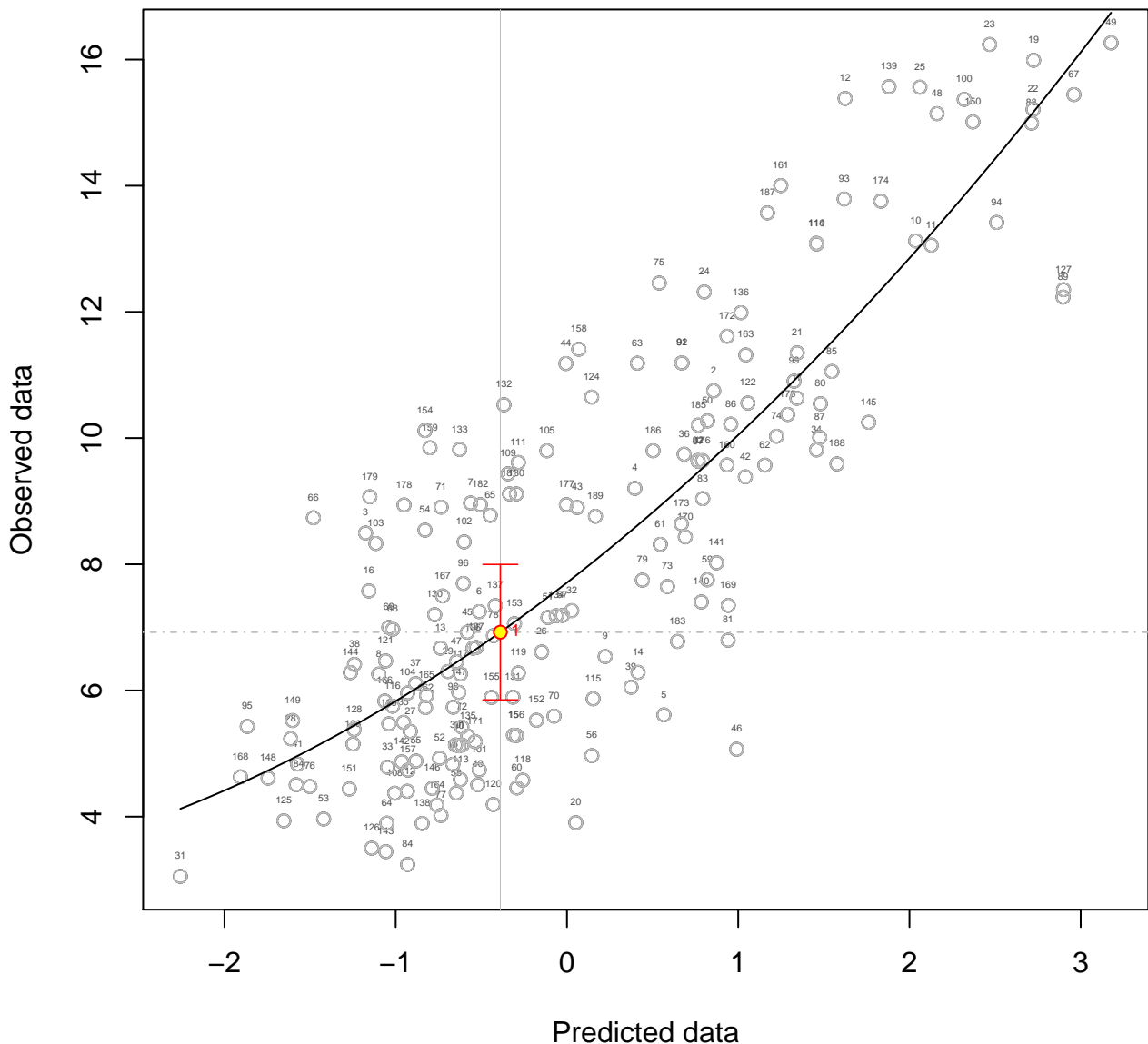

# ENTHAL (kJ/Kg)

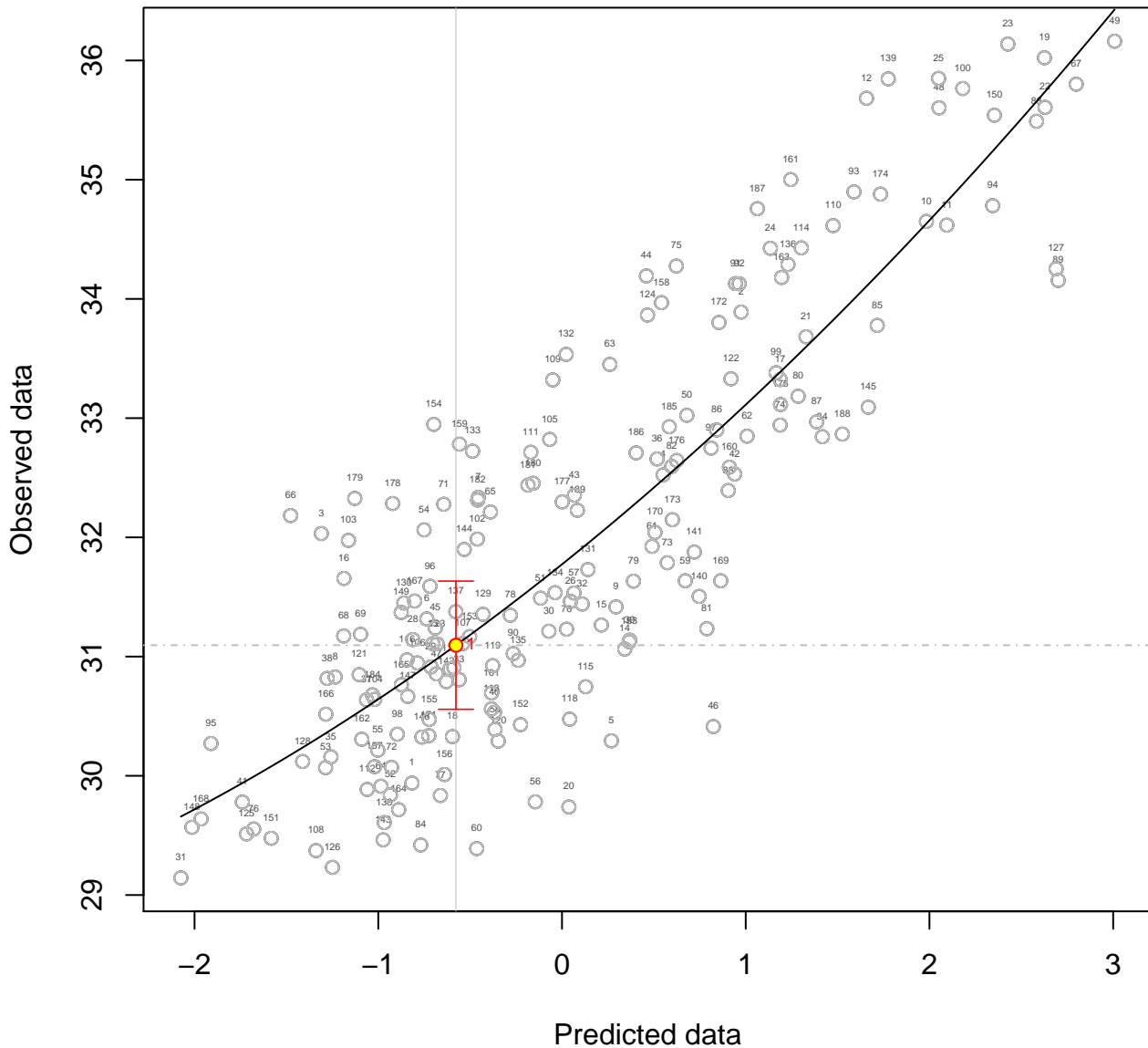

Supplement: Supplementary Material [file rsos192067supp1.zip › Supplementary Material S1-S5/S5_CLAMP_Vegora/CLAMP analysis/Run/PhysgAsia1_HiResGridMetAsia1/PhysgAsia1_HiResGridMetAsia1.pdf]

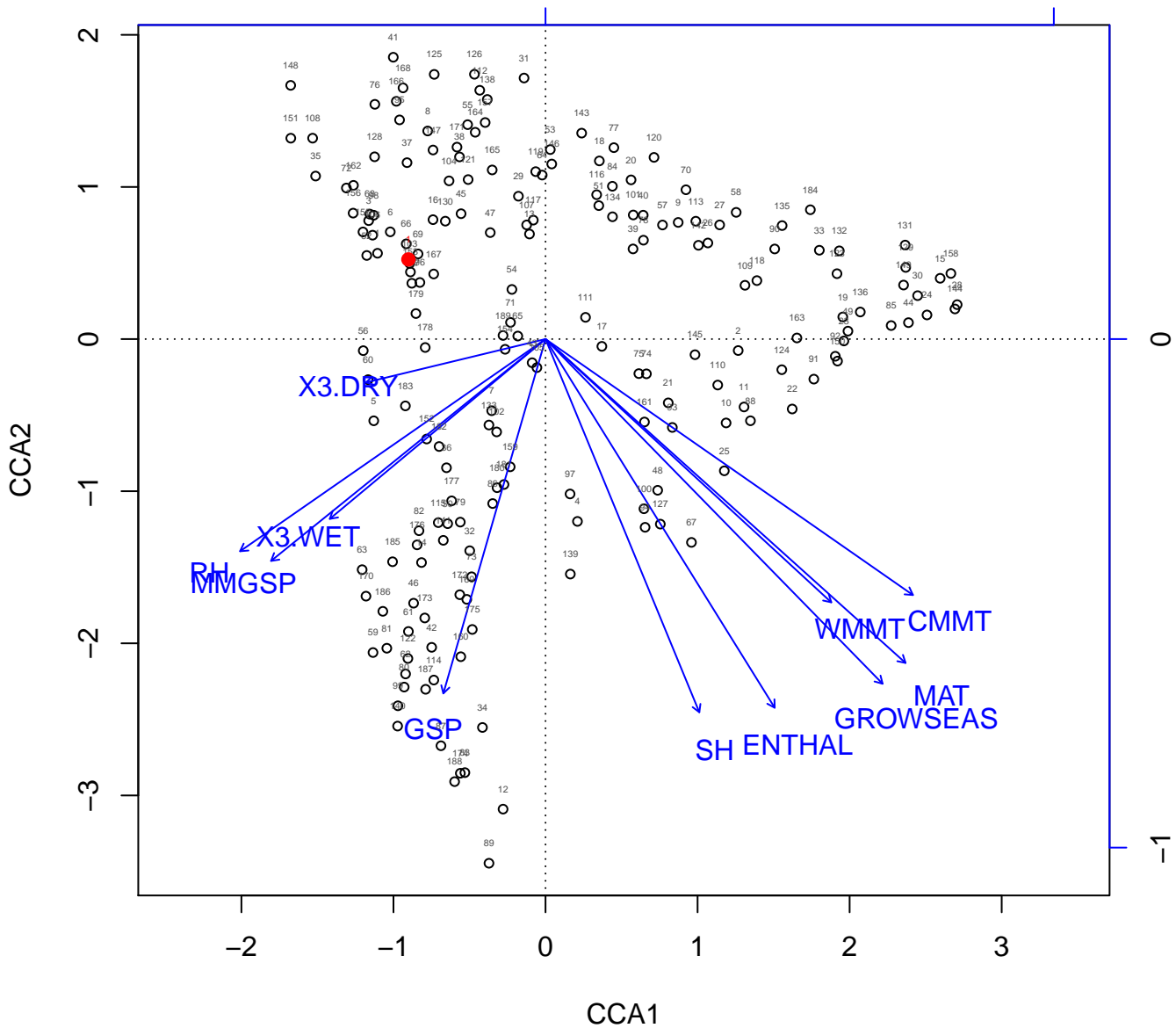

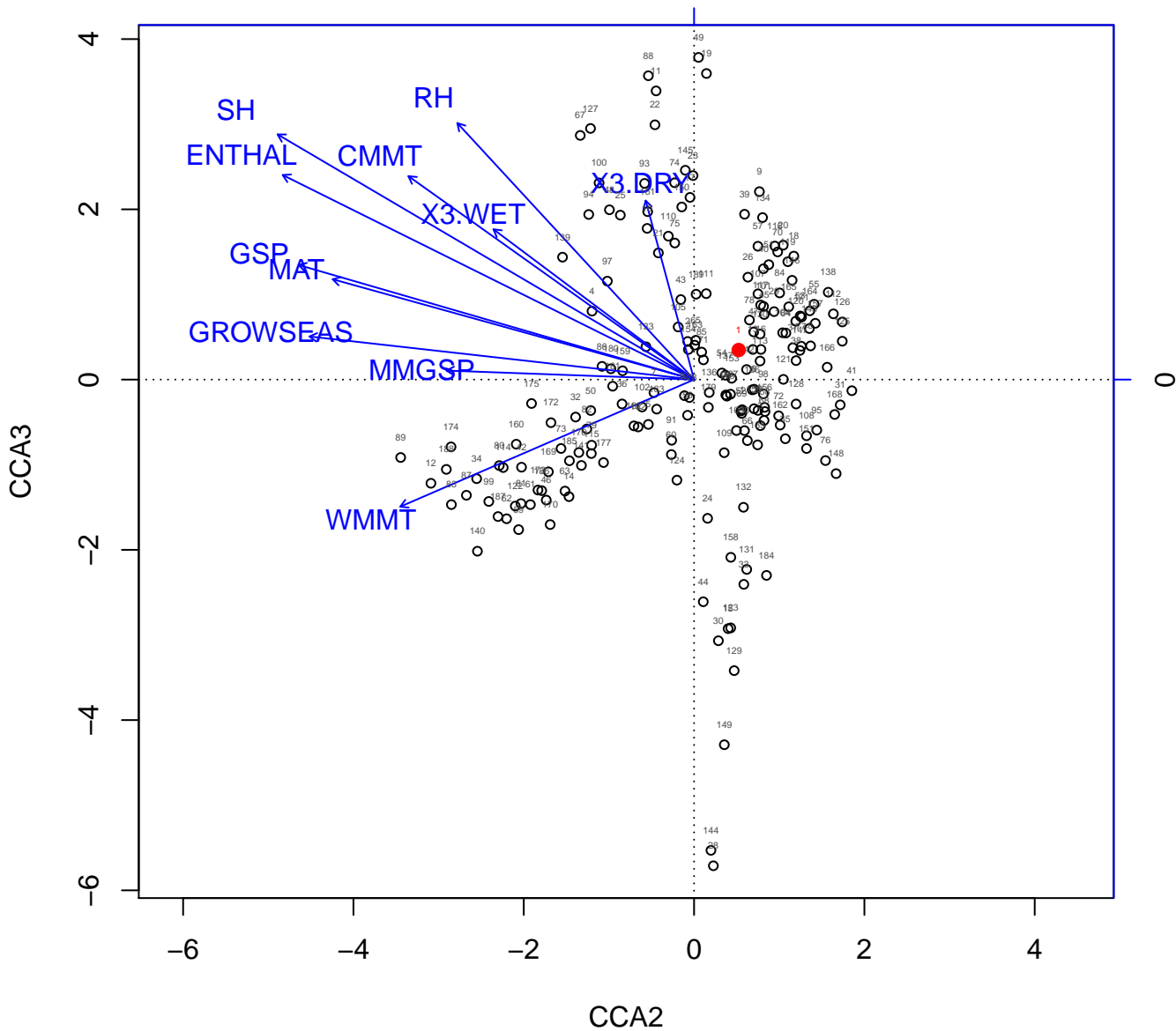

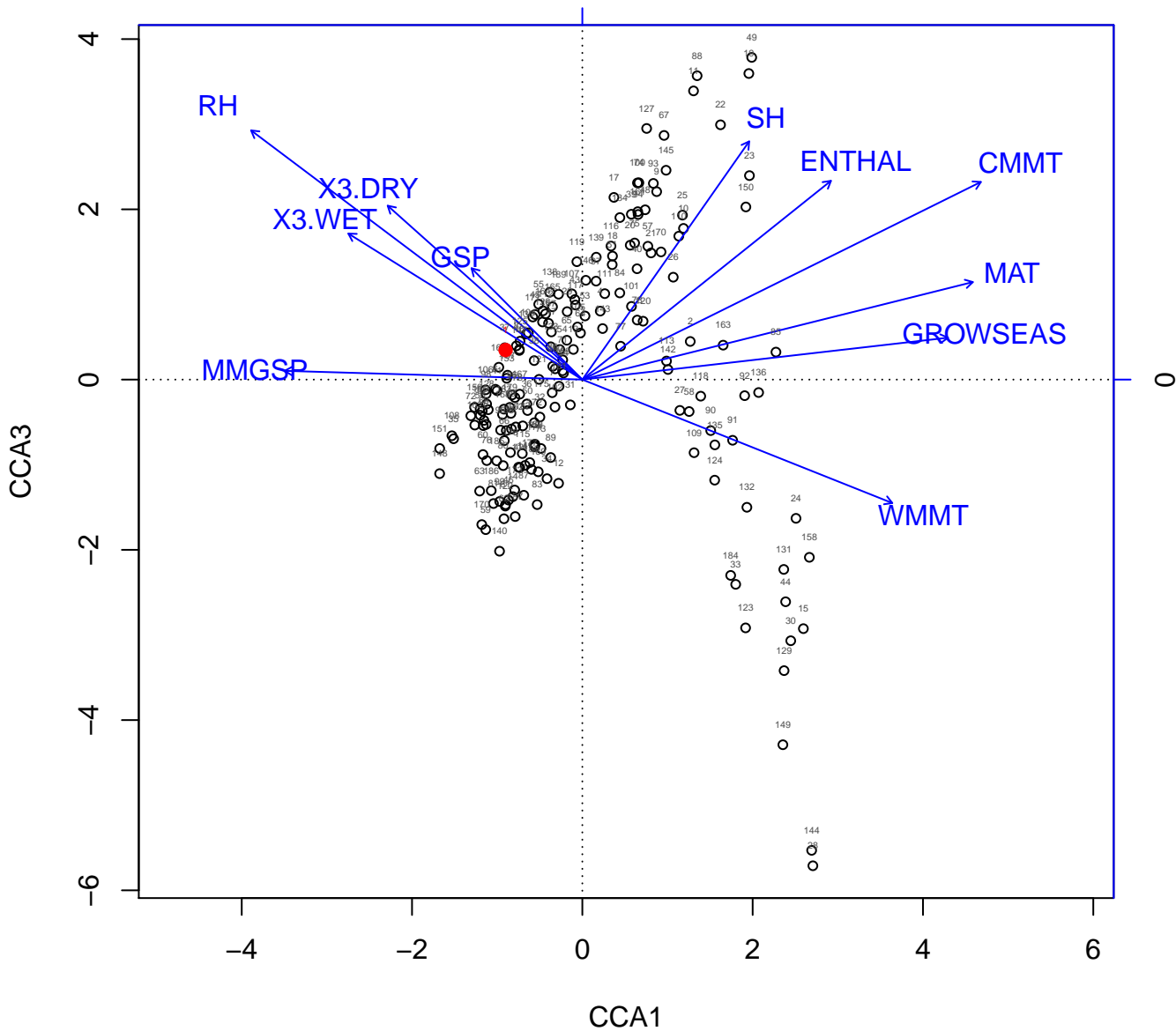

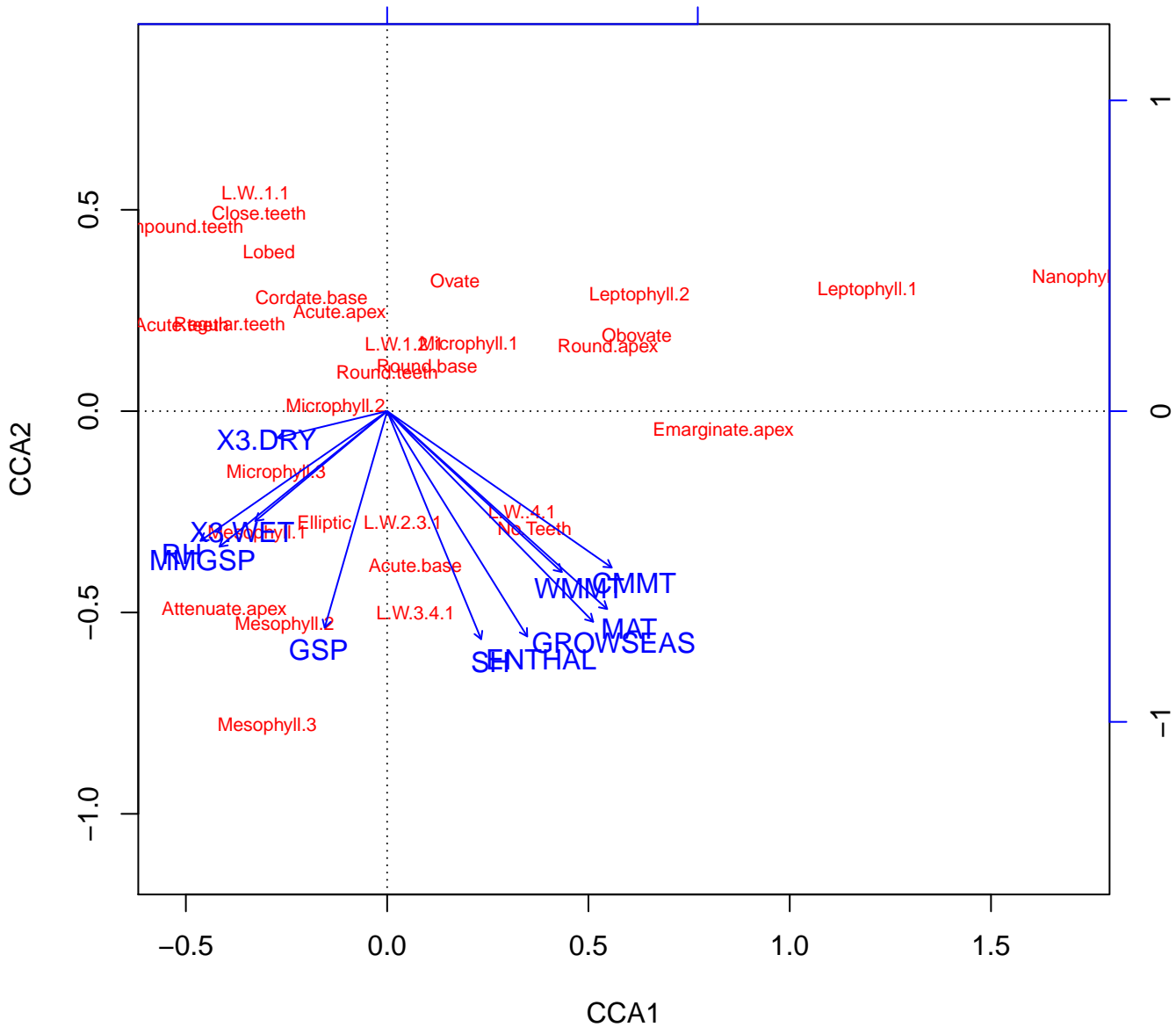

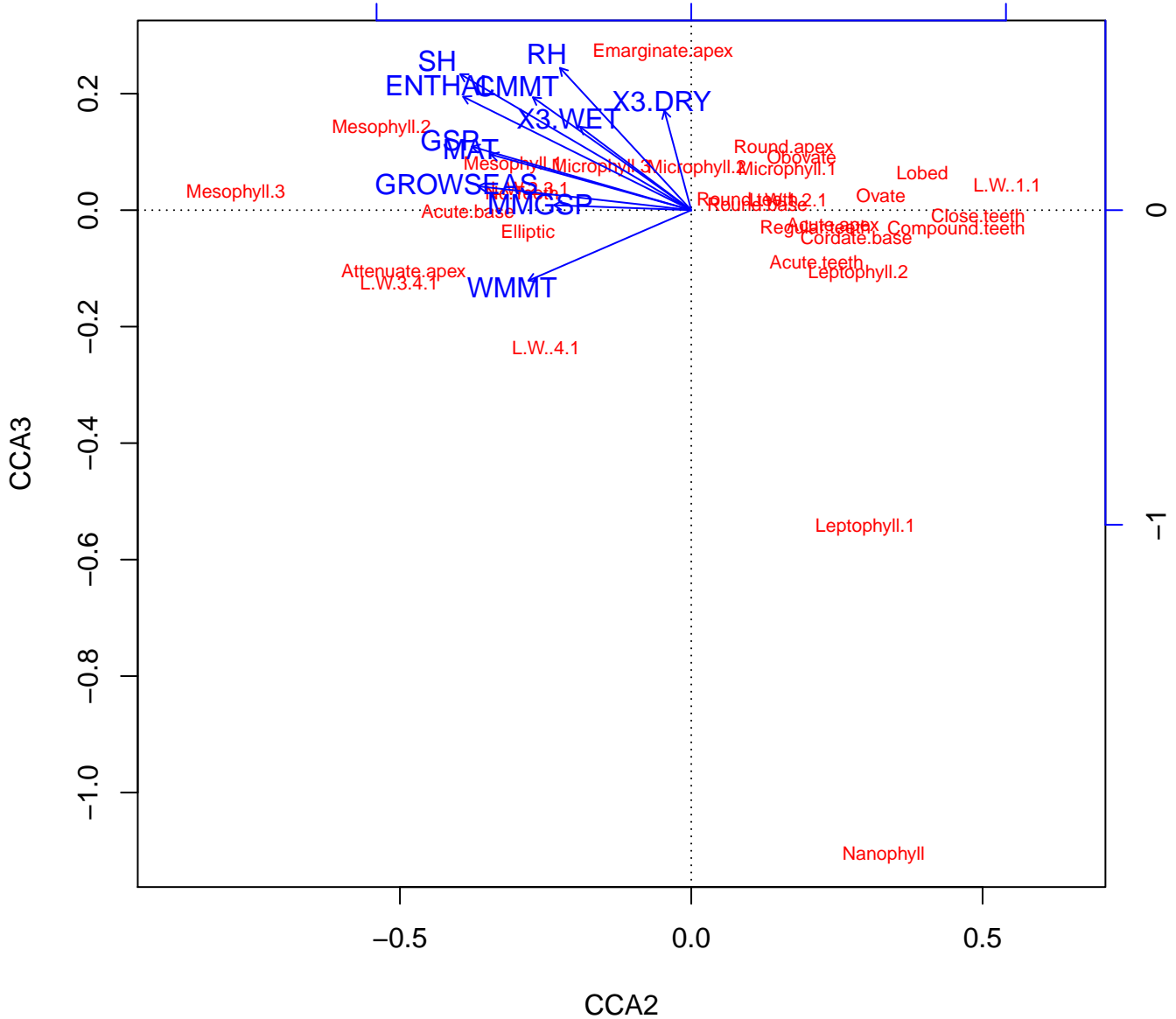

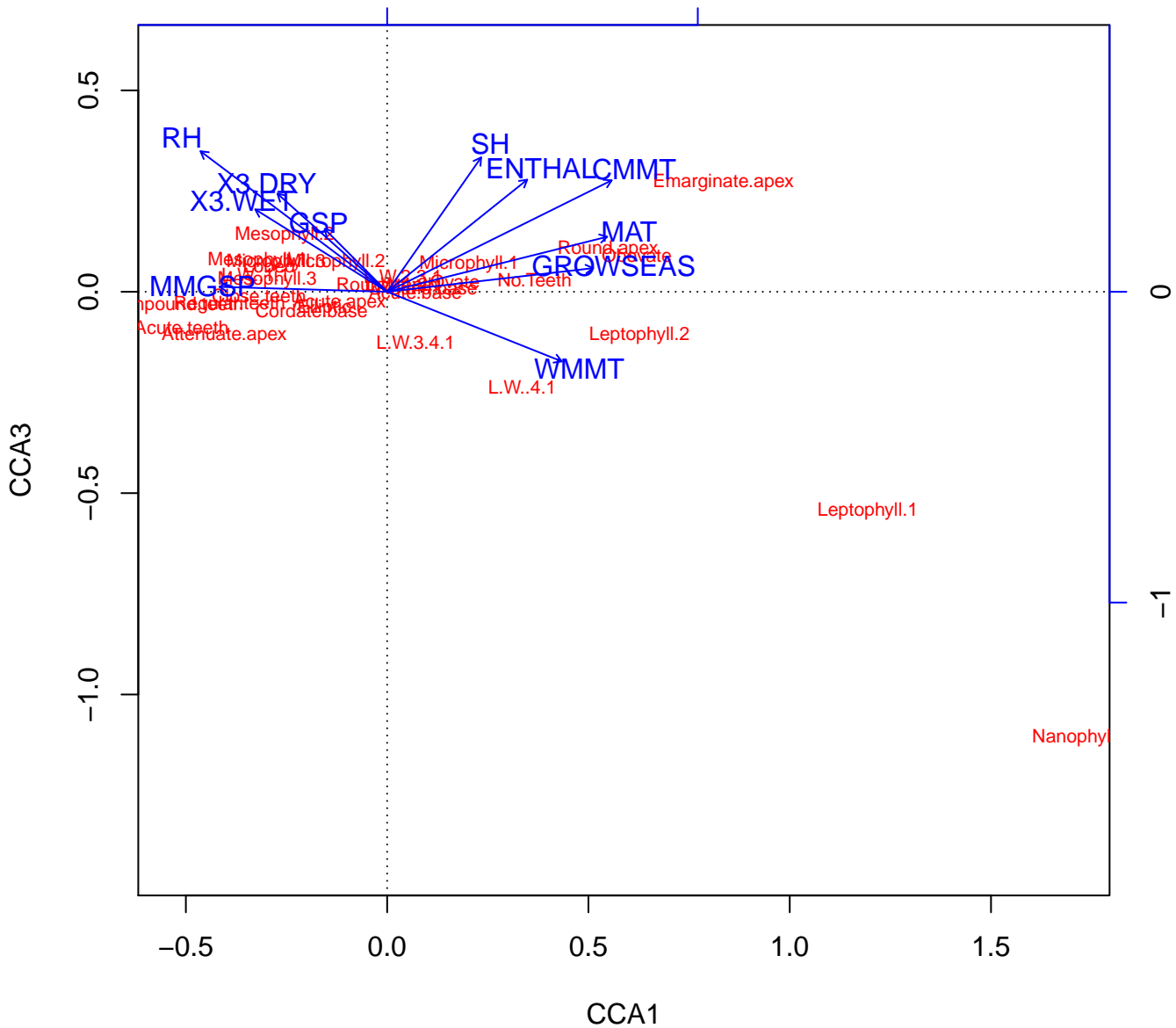

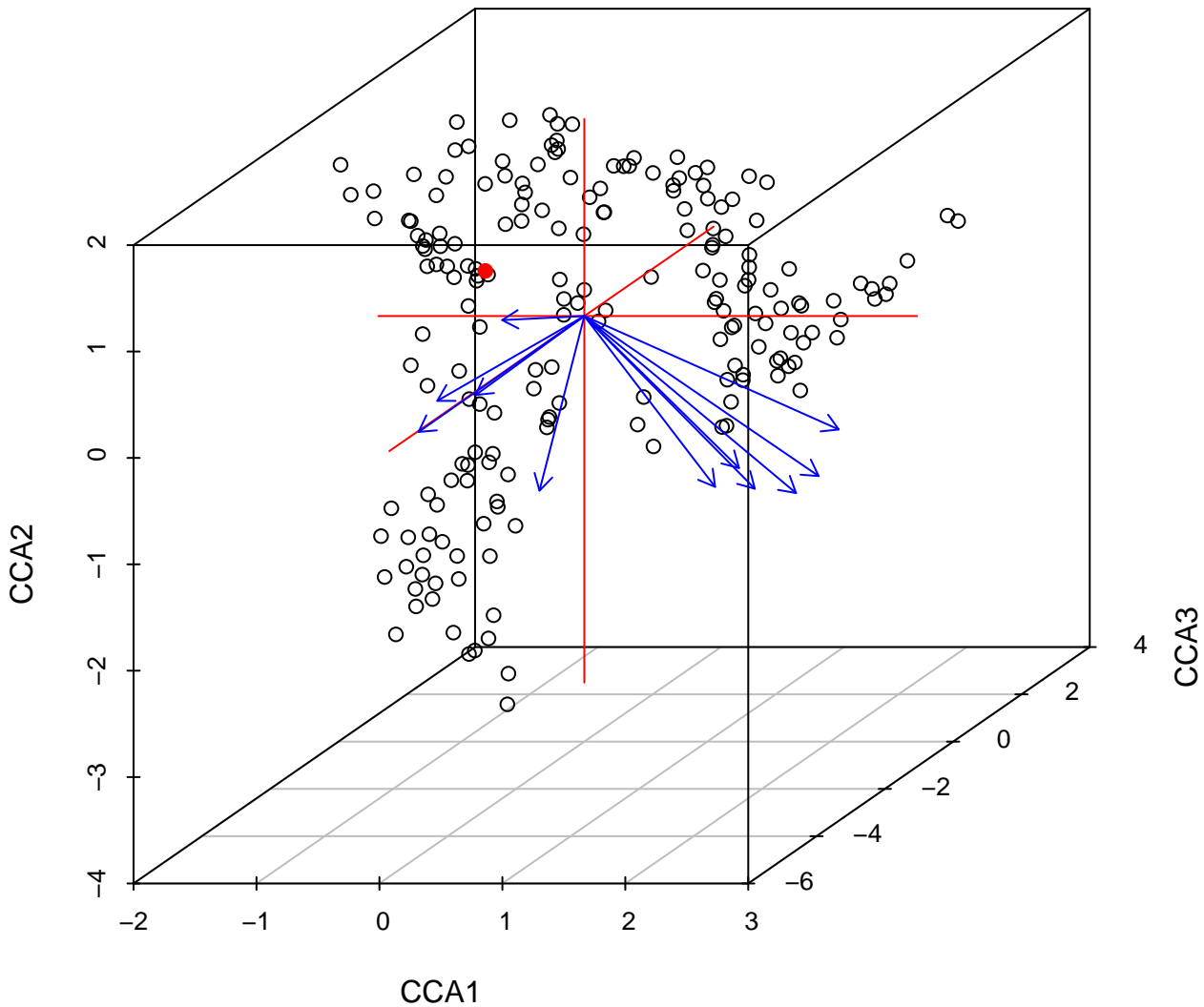

Supplement: Supplementary Material [file rsos192067supp1.zip › Supplementary Material S1-S5/S5_CLAMP_Vegora/CLAMP analysis/Run/PhysgAsia1_HiResGridMetAsia1/CCAGraph.pdf]

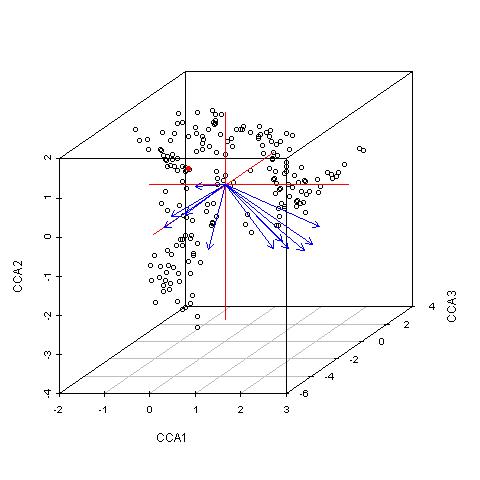

Supplement: Supplementary Material [file rsos192067supp1.zip › Supplementary Material S1-S5/S5_CLAMP_Vegora/CLAMP analysis/Run/PhysgAsia1_HiResGridMetAsia1/CCA3d.jpg]

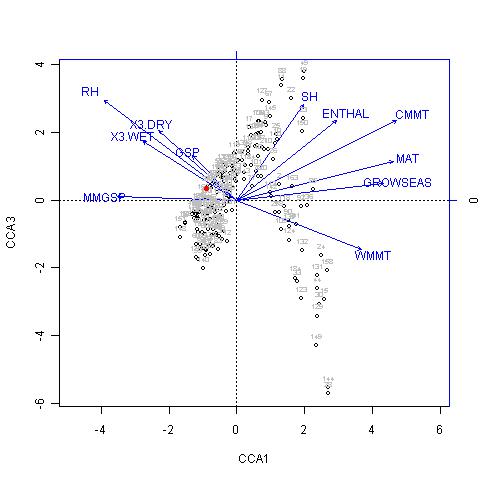

Supplement: Supplementary Material [file rsos192067supp1.zip › Supplementary Material S1-S5/S5_CLAMP_Vegora/CLAMP analysis/Run/PhysgAsia1_HiResGridMetAsia1/CCA1vsCCA3.jpg]

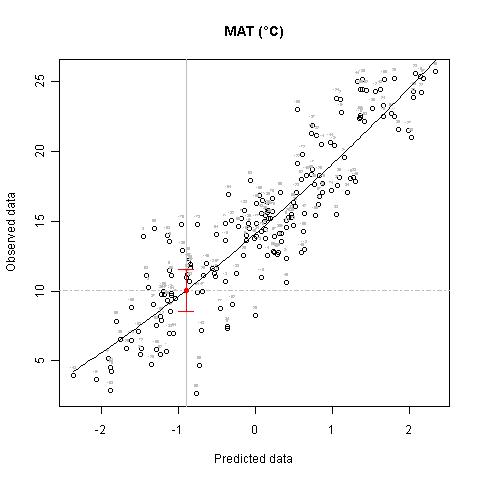

Supplement: Supplementary Material [file rsos192067supp1.zip › Supplementary Material S1-S5/S5_CLAMP_Vegora/CLAMP analysis/Run/PhysgAsia1_HiResGridMetAsia1/MAT.jpg]

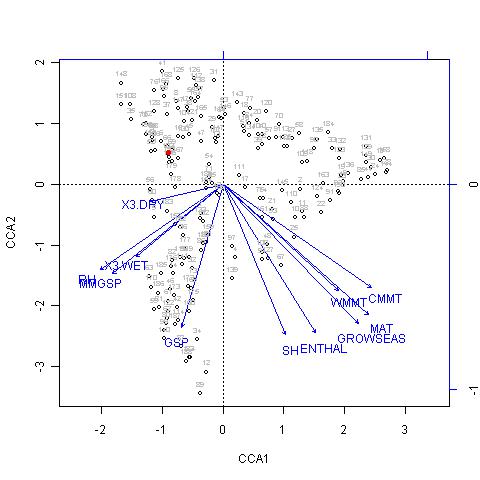

Supplement: Supplementary Material [file rsos192067supp1.zip › Supplementary Material S1-S5/S5_CLAMP_Vegora/CLAMP analysis/Run/PhysgAsia1_HiResGridMetAsia1/CCA1vsCCA2.jpg]

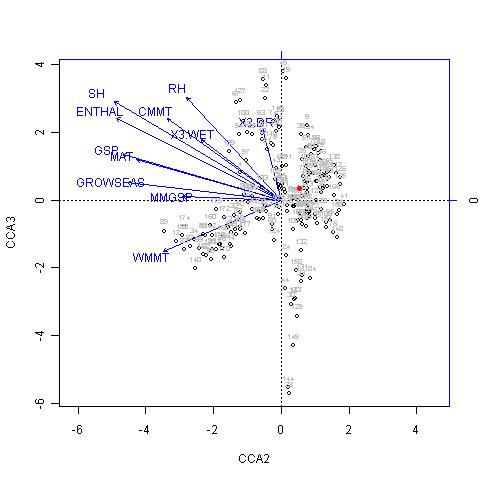

Supplement: Supplementary Material [file rsos192067supp1.zip › Supplementary Material S1-S5/S5_CLAMP_Vegora/CLAMP analysis/Run/PhysgAsia1_HiResGridMetAsia1/CCA2vsCCA3.jpg]

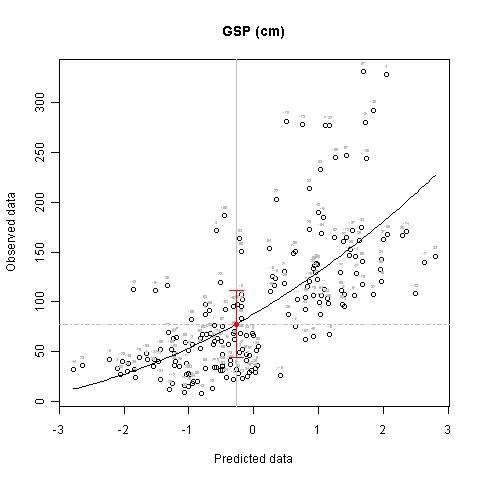

Supplement: Supplementary Material [file rsos192067supp1.zip › Supplementary Material S1-S5/S5_CLAMP_Vegora/CLAMP analysis/Run/PhysgAsia1_HiResGridMetAsia1/GSP.jpg]

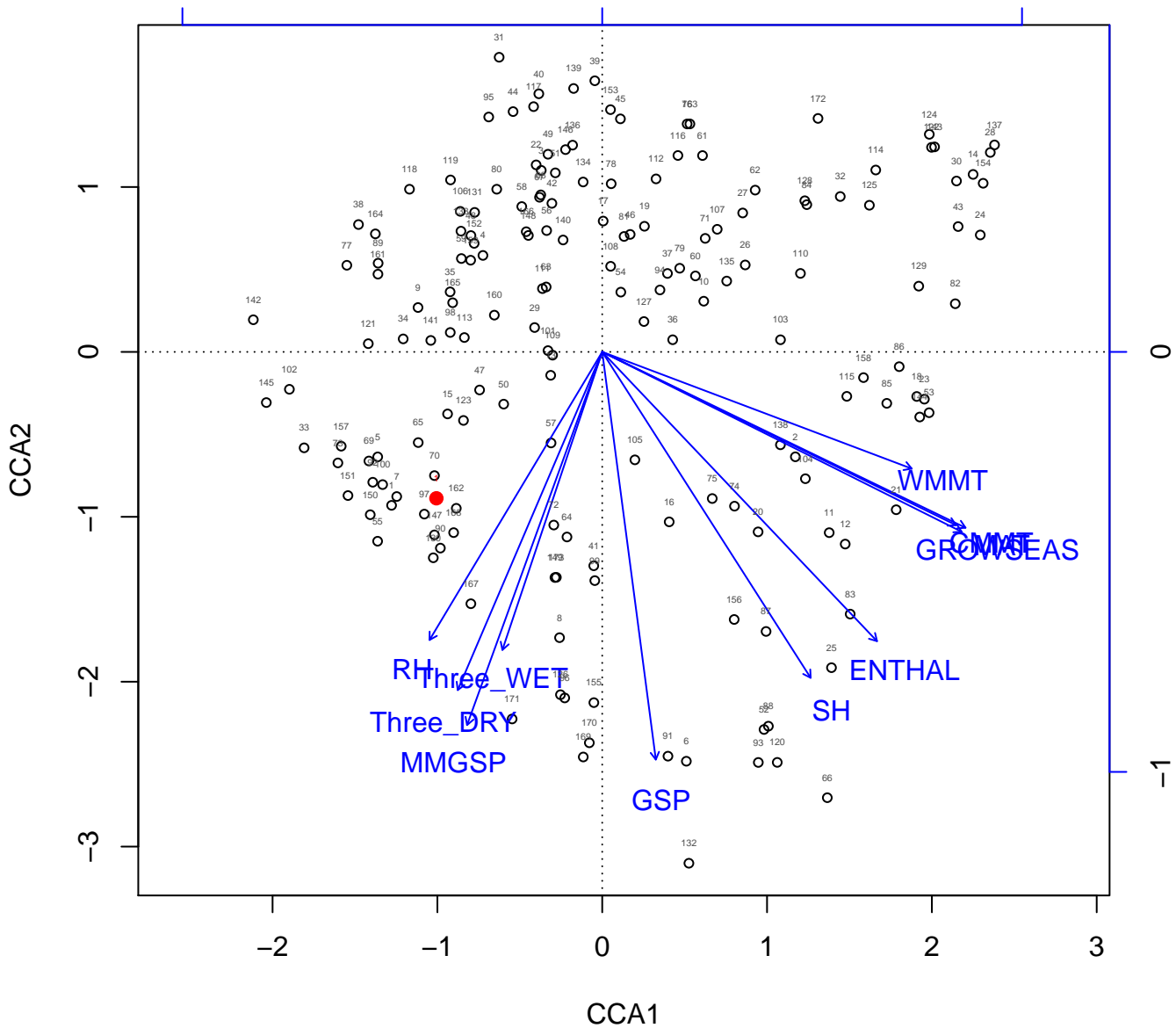

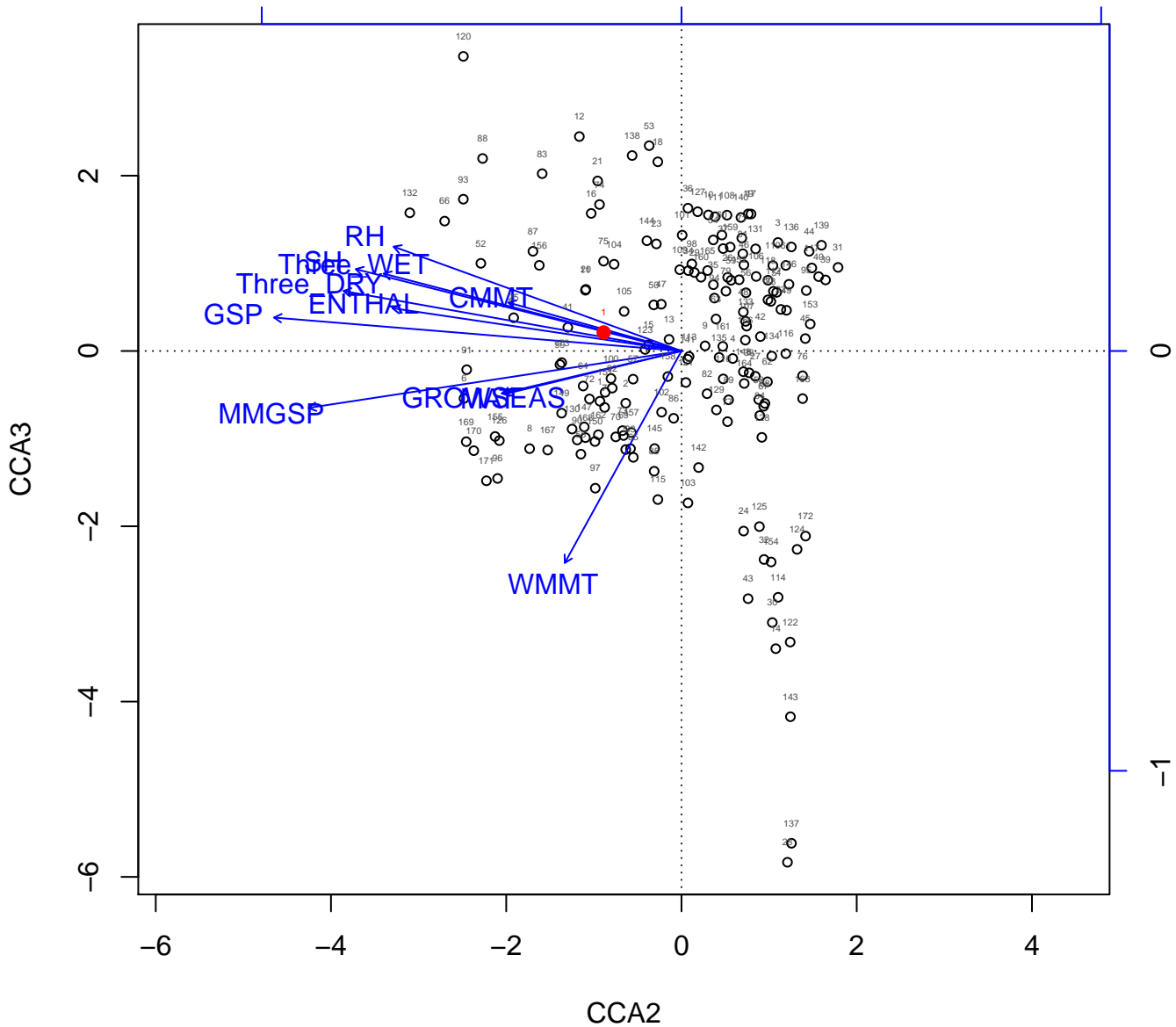



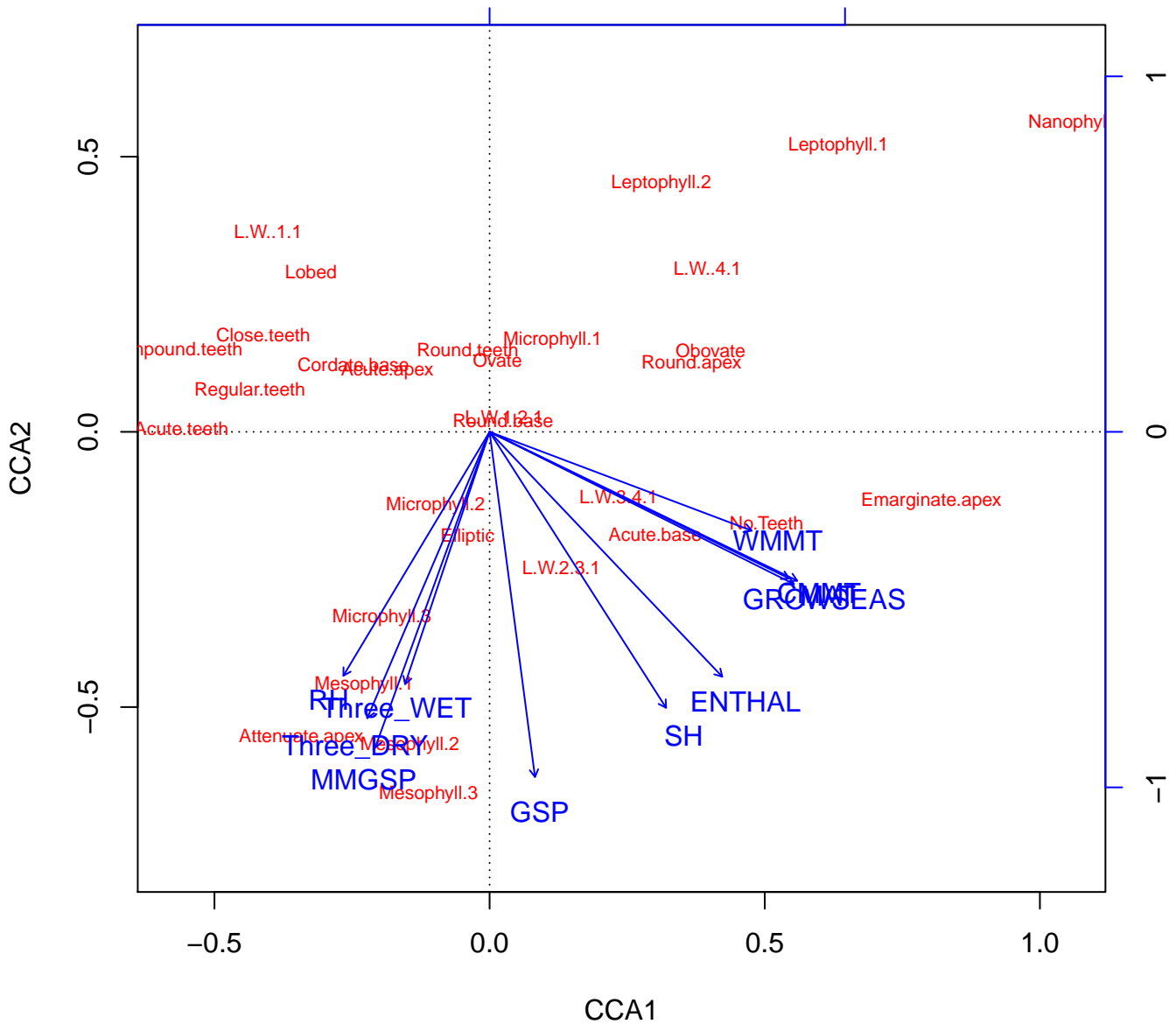



CCA3

0.0

-0.5

-1.0

-0.5

0.0

0.5

1.0

CCA1

0

-1

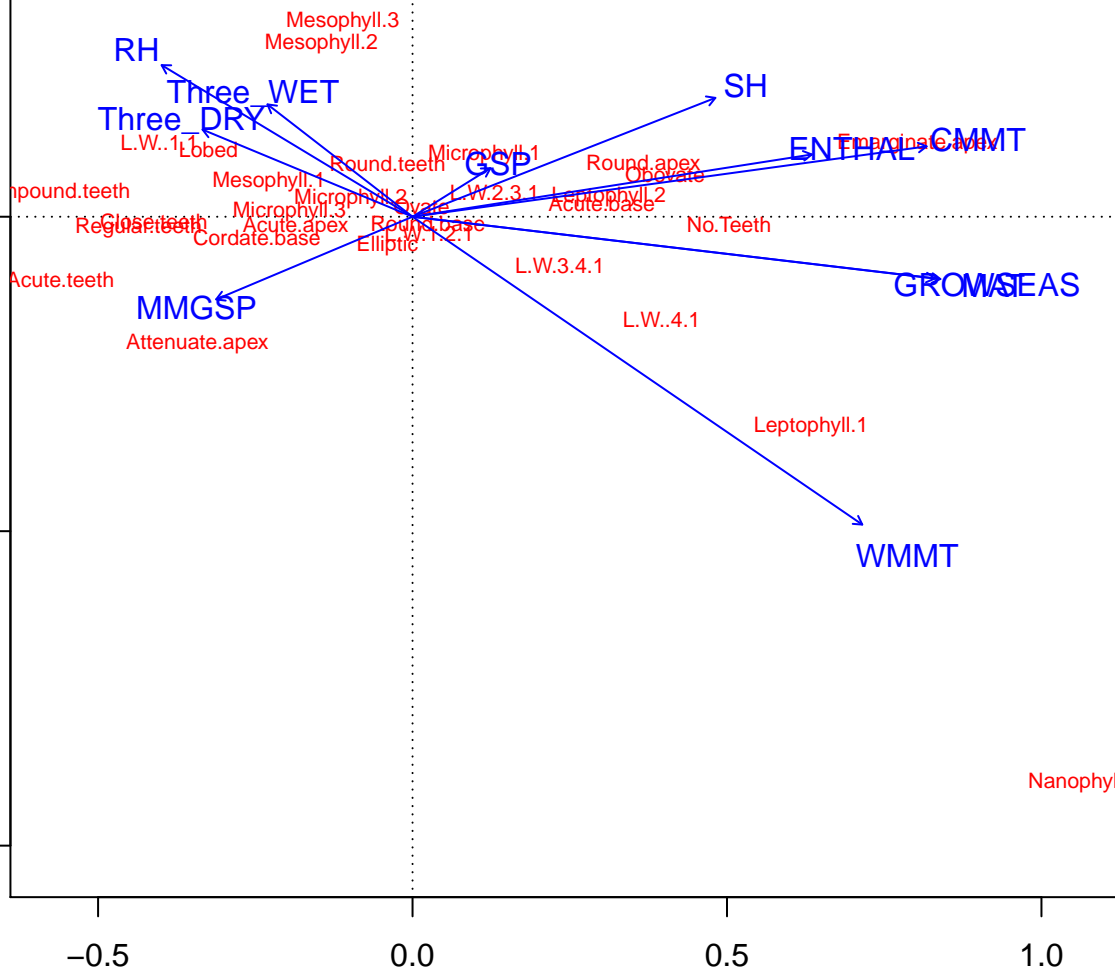

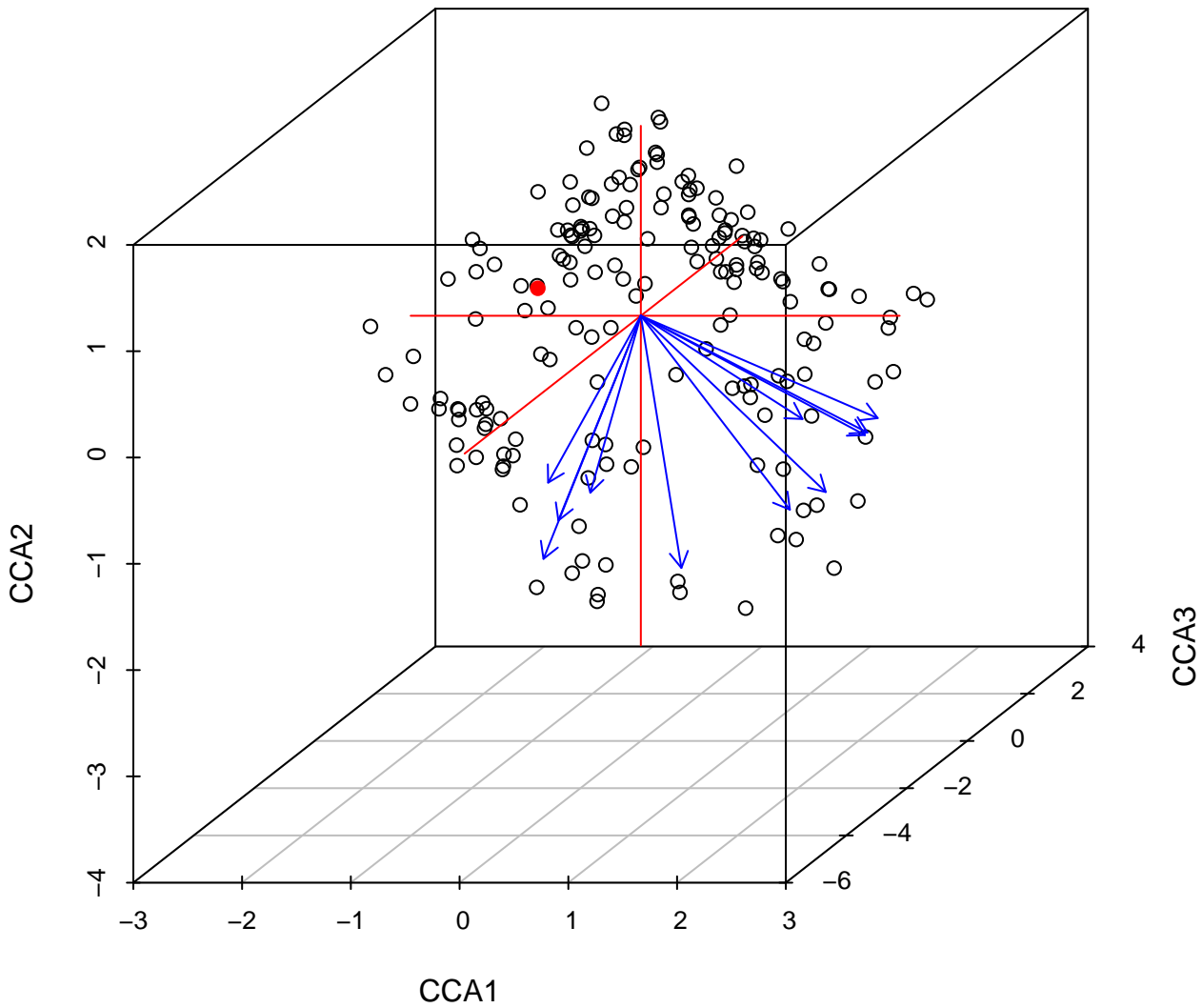

Supplement: Supplementary Material [file rsos192067supp1.zip › Supplementary Material S1-S5/S5_CLAMP_Vegora/CLAMP analysis/Run/Physg3arcAZ_GRIDMet3arAZ/CCAGraph.pdf]

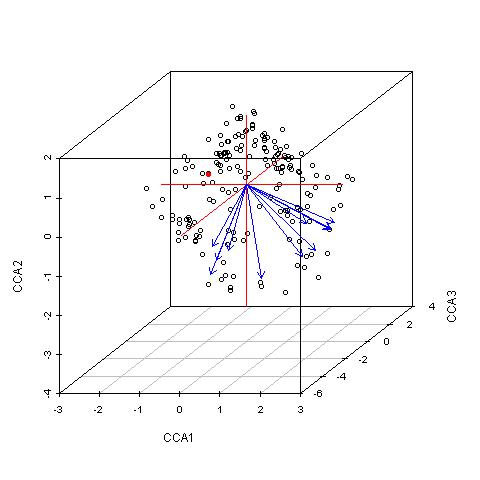

Supplement: Supplementary Material [file rsos192067supp1.zip › Supplementary Material S1-S5/S5_CLAMP_Vegora/CLAMP analysis/Run/Physg3arcAZ_GRIDMet3arAZ/CCA3d.jpg]

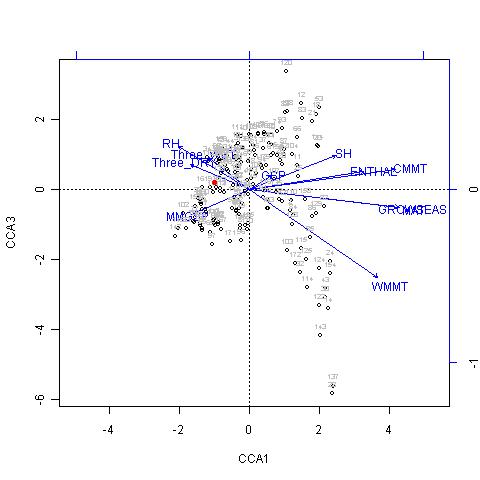

Supplement: Supplementary Material [file rsos192067supp1.zip › Supplementary Material S1-S5/S5_CLAMP_Vegora/CLAMP analysis/Run/Physg3arcAZ_GRIDMet3arAZ/CCA1vsCCA3.jpg]

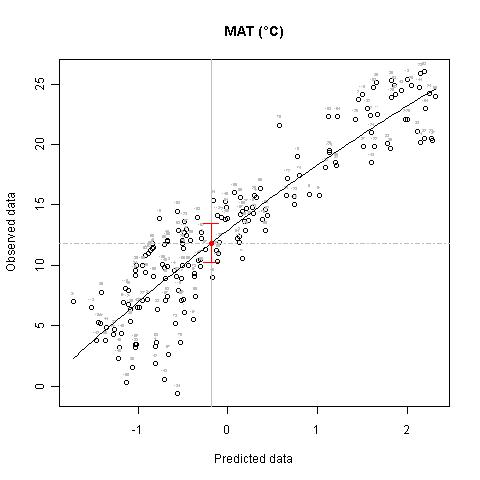

Supplement: Supplementary Material [file rsos192067supp1.zip › Supplementary Material S1-S5/S5_CLAMP_Vegora/CLAMP analysis/Run/Physg3arcAZ_GRIDMet3arAZ/MAT.jpg]

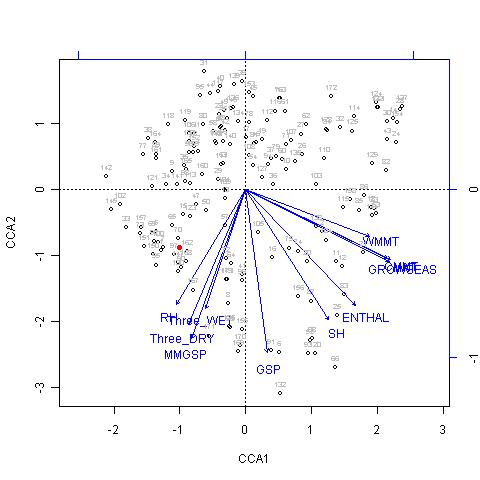

Supplement: Supplementary Material [file rsos192067supp1.zip › Supplementary Material S1-S5/S5_CLAMP_Vegora/CLAMP analysis/Run/Physg3arcAZ_GRIDMet3arAZ/CCA1vsCCA2.jpg]

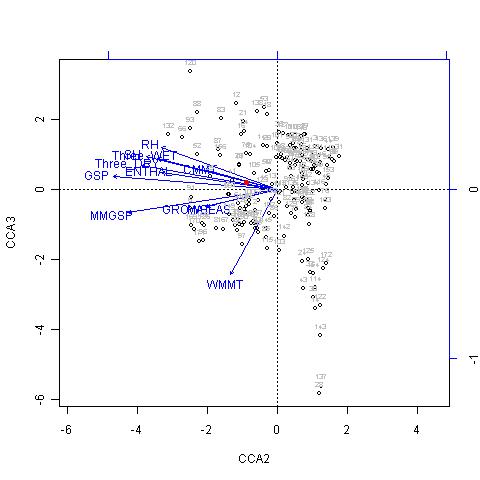

Supplement: Supplementary Material [file rsos192067supp1.zip › Supplementary Material S1-S5/S5_CLAMP_Vegora/CLAMP analysis/Run/Physg3arcAZ_GRIDMet3arAZ/CCA2vsCCA3.jpg]

MAT (°C)

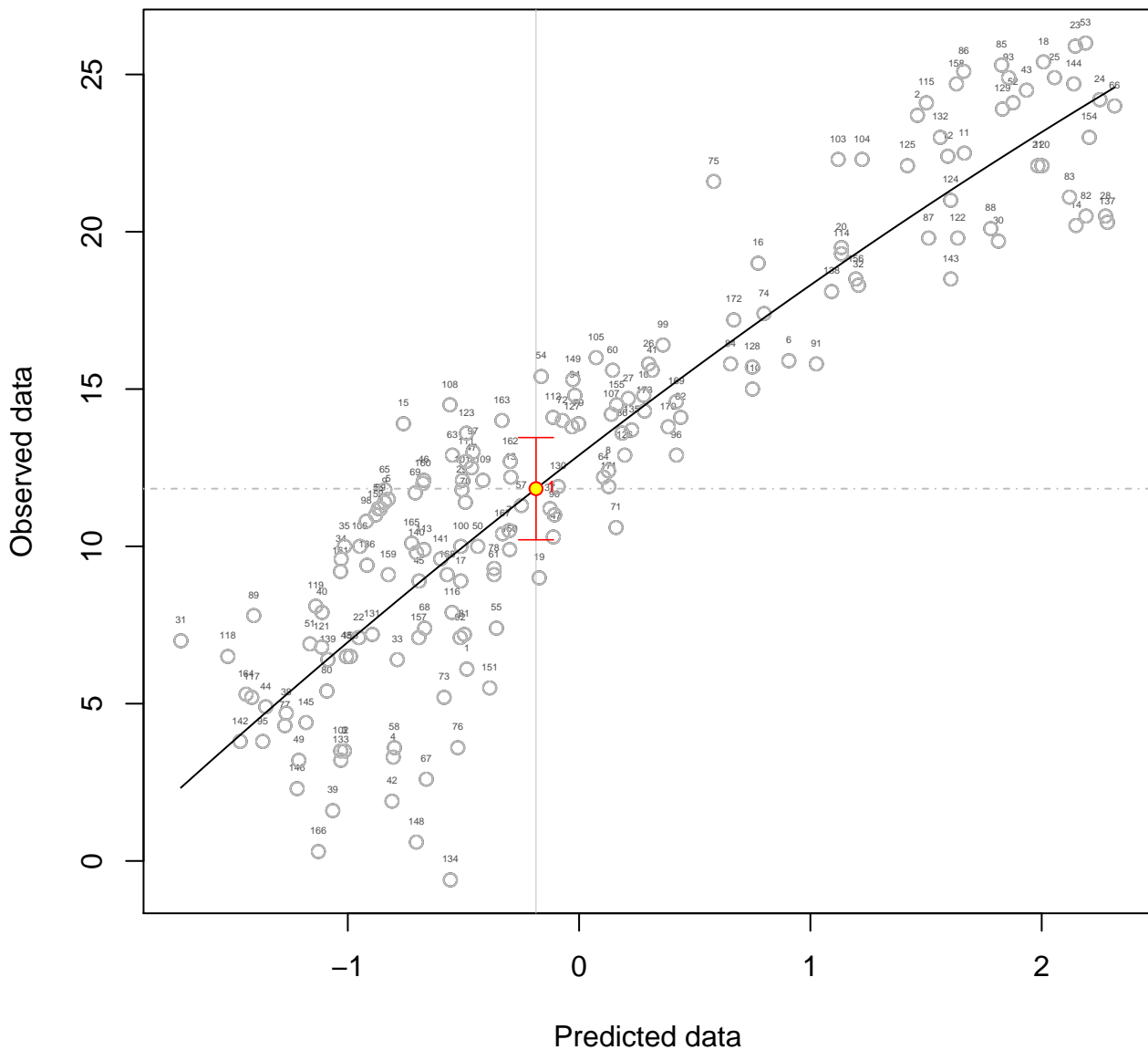

WMMT (°C)

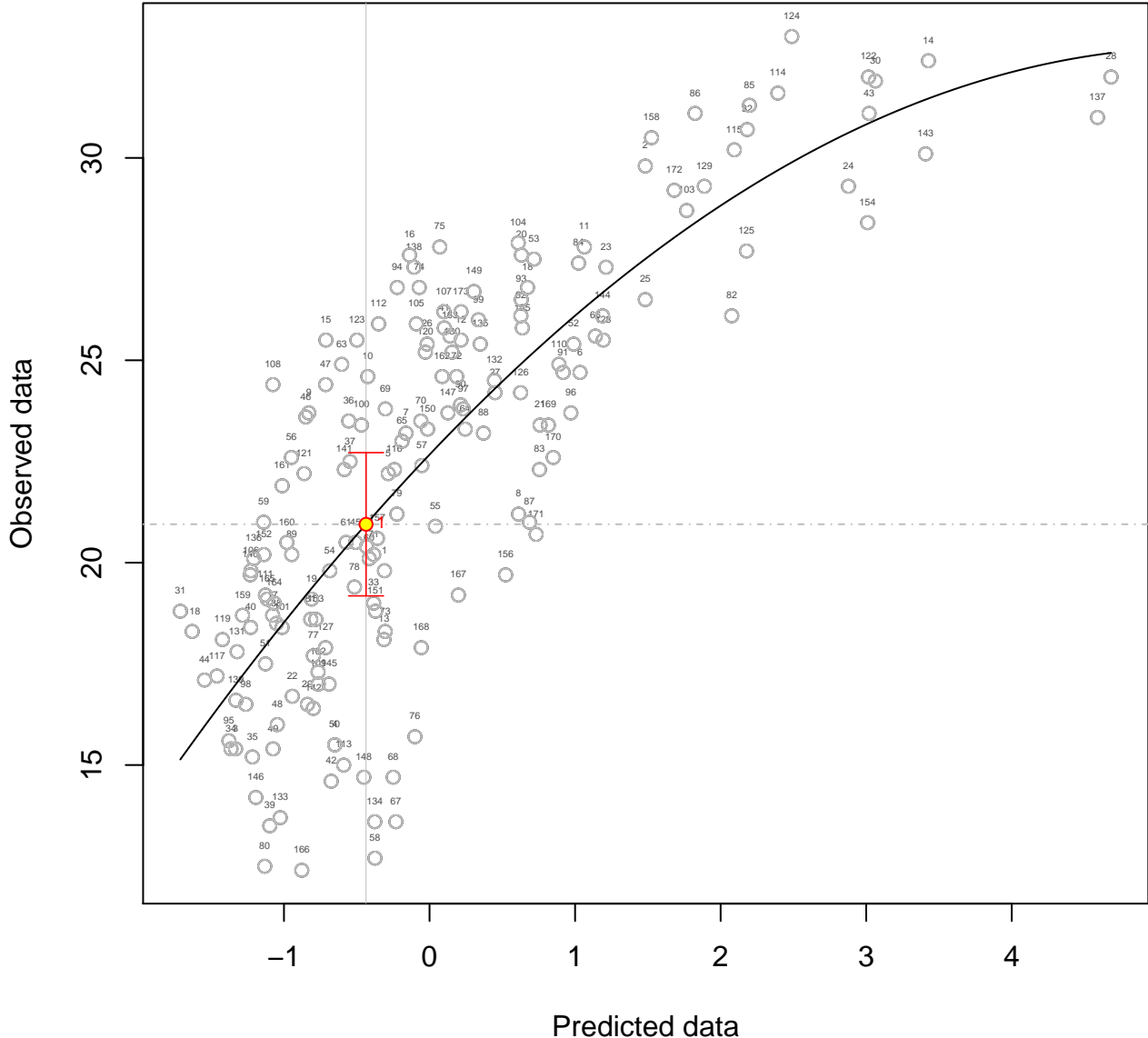

CMMT (°C)

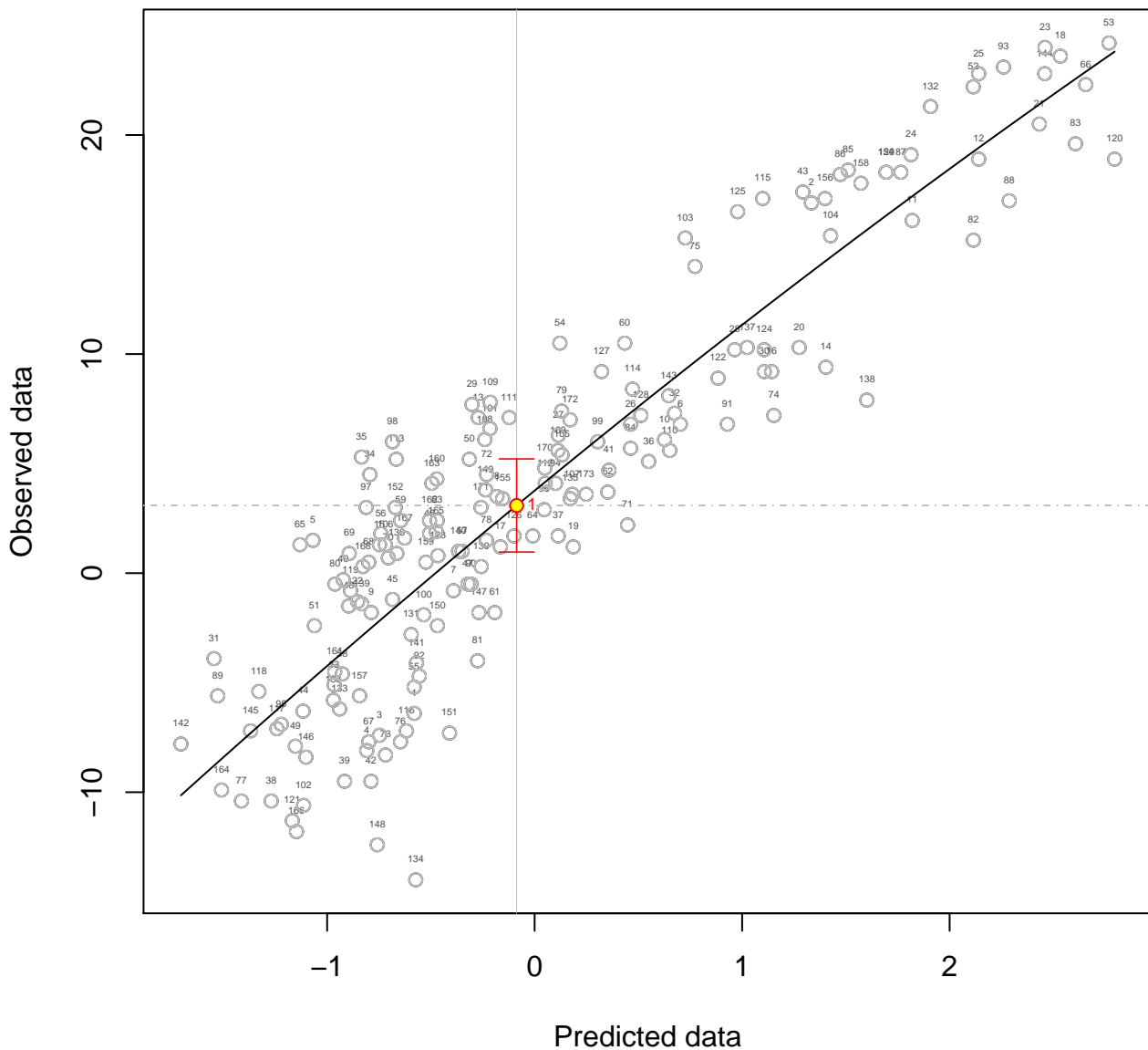

GROWSEAS (months)

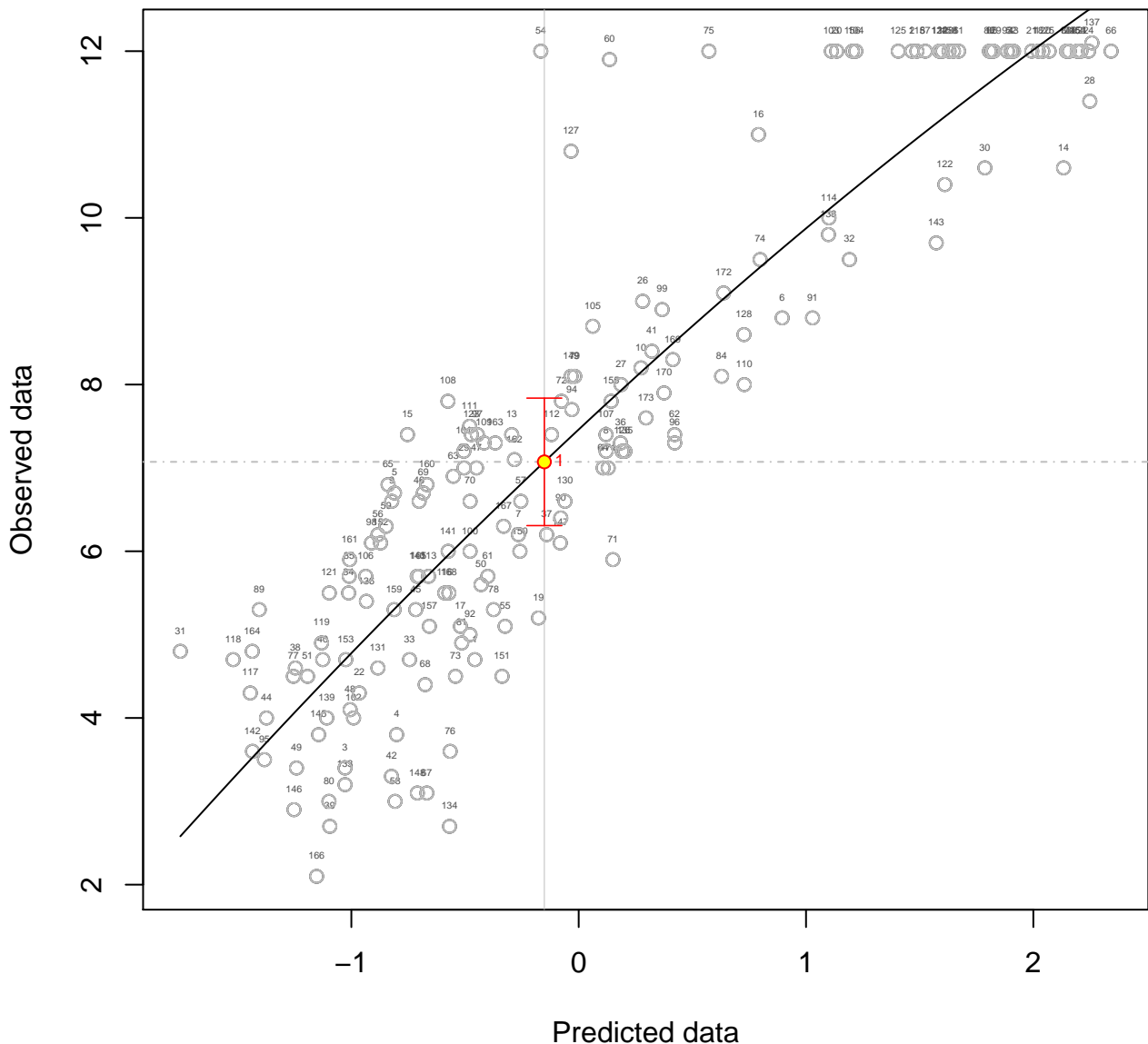

GSP (cm)

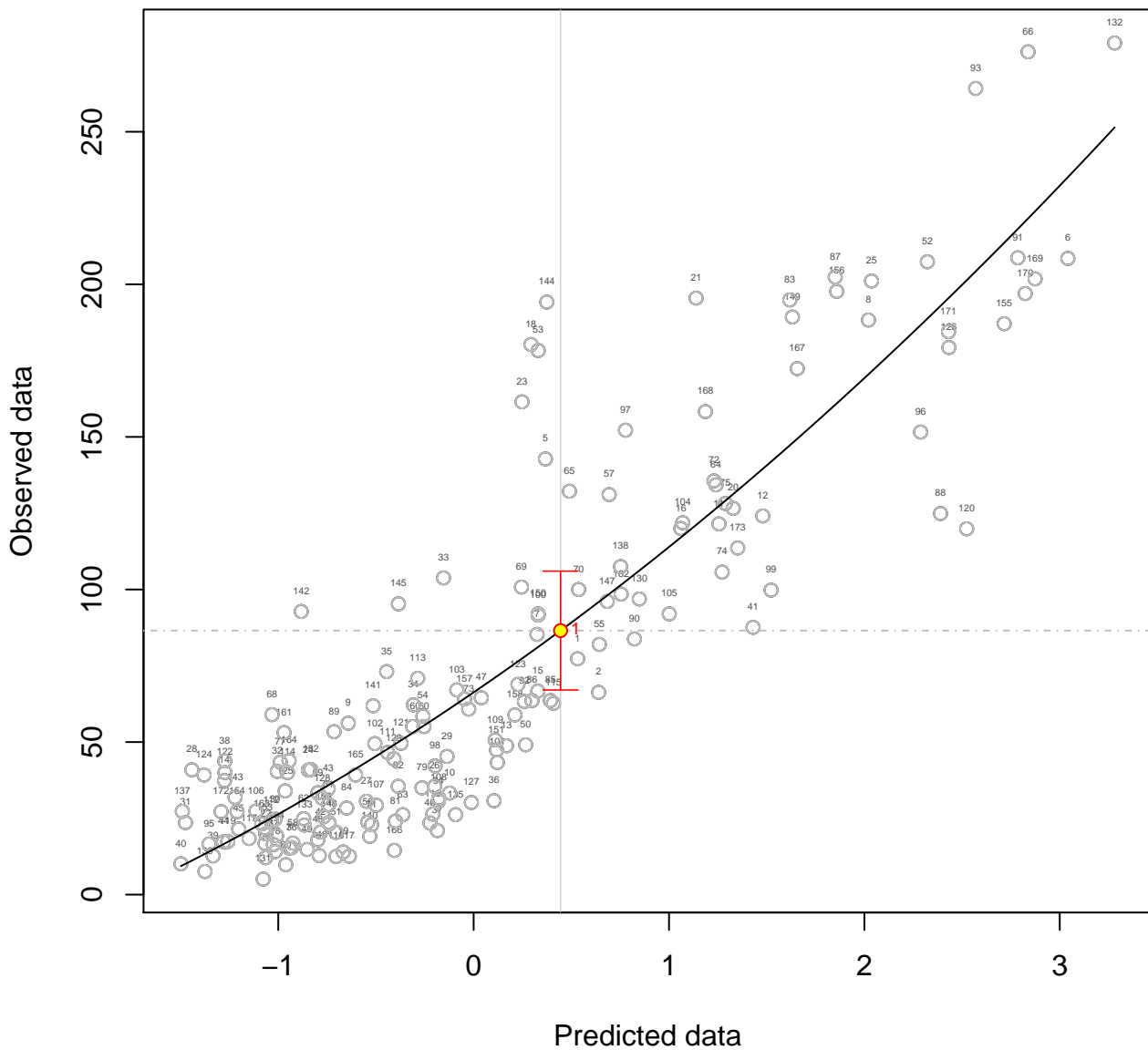

MMGSP (cm)

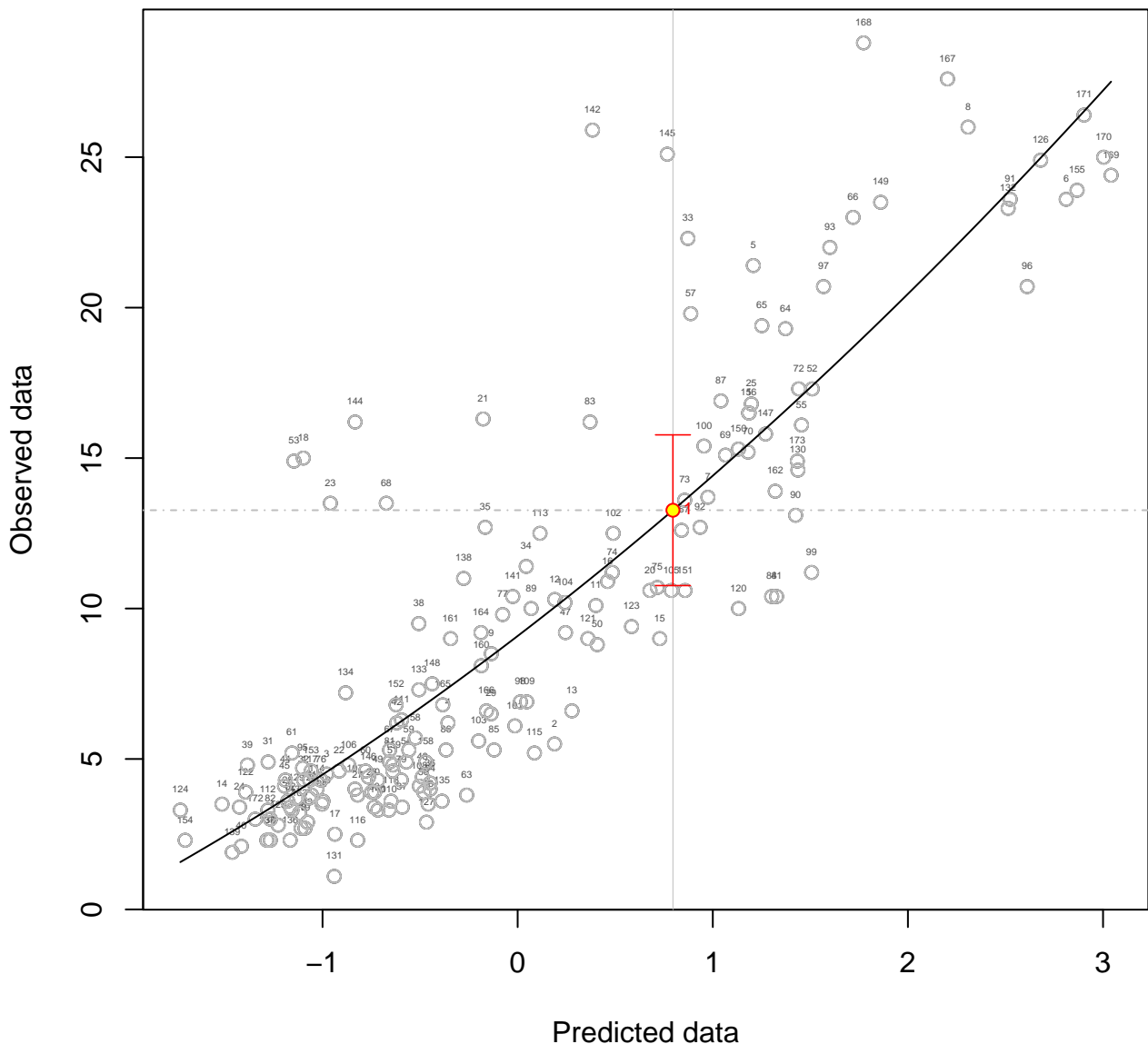

# Three\_WET (cm)

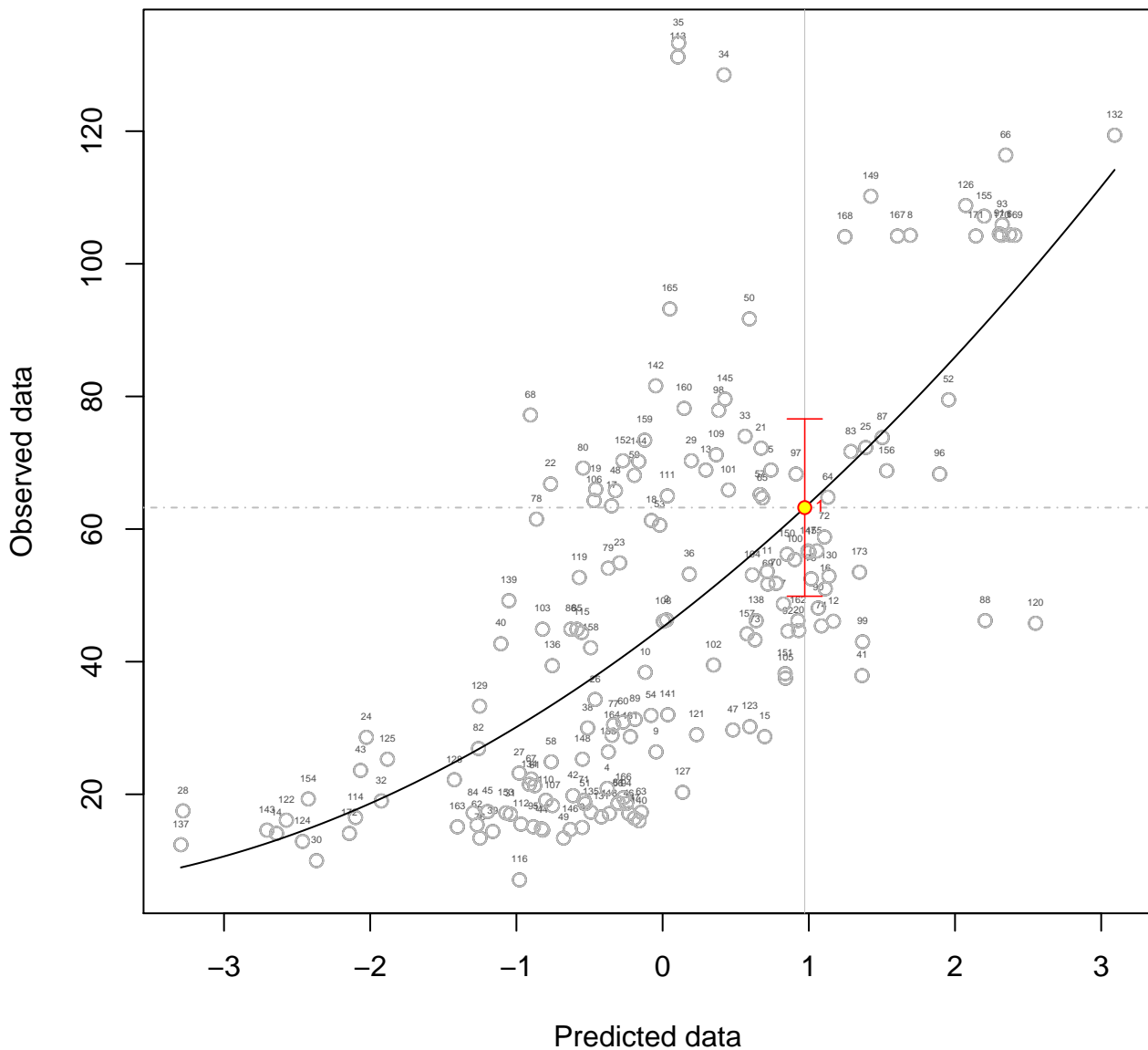

# Three\_DRY (cm)

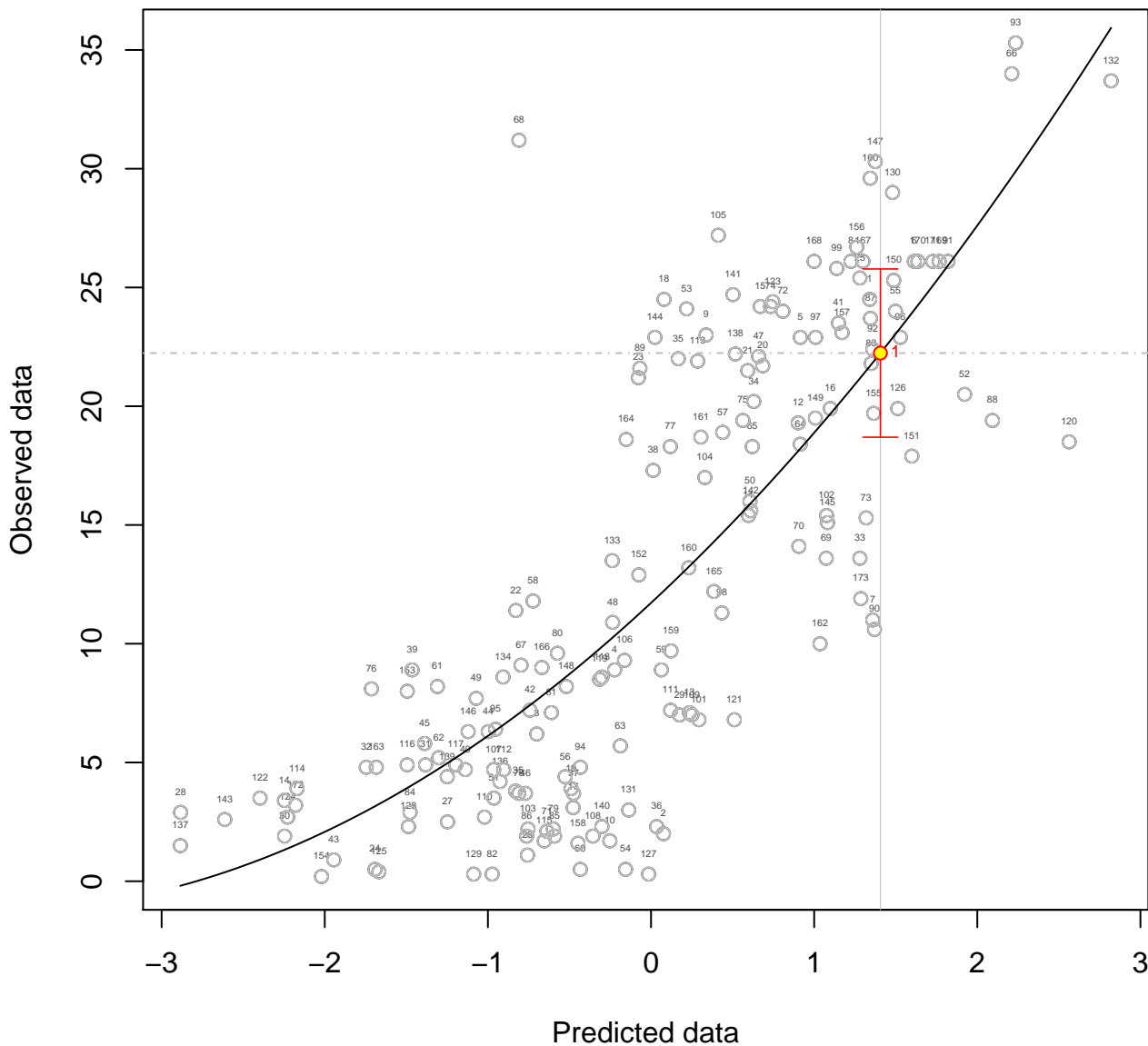

RH (%)

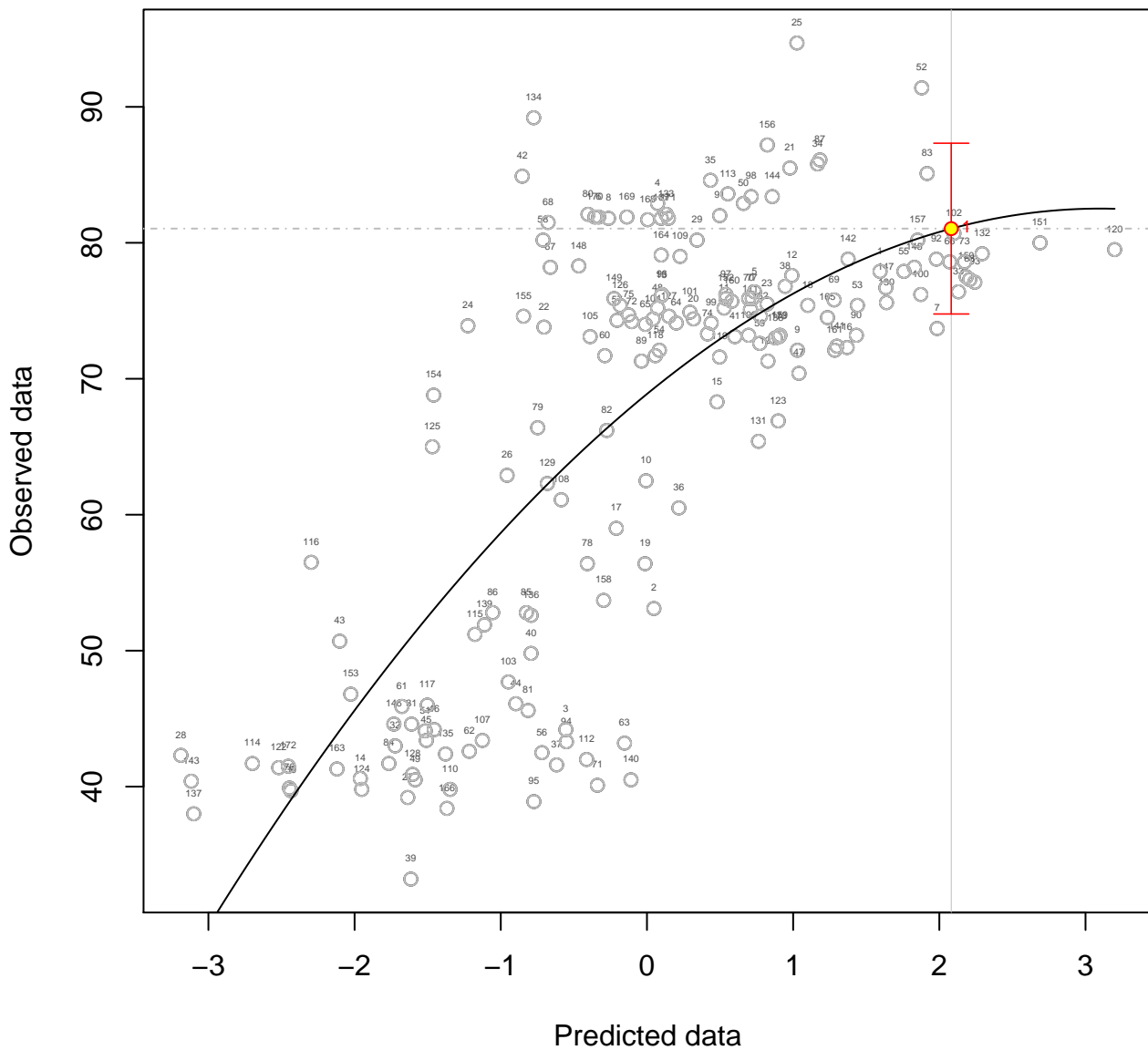

SH (g/Kg)

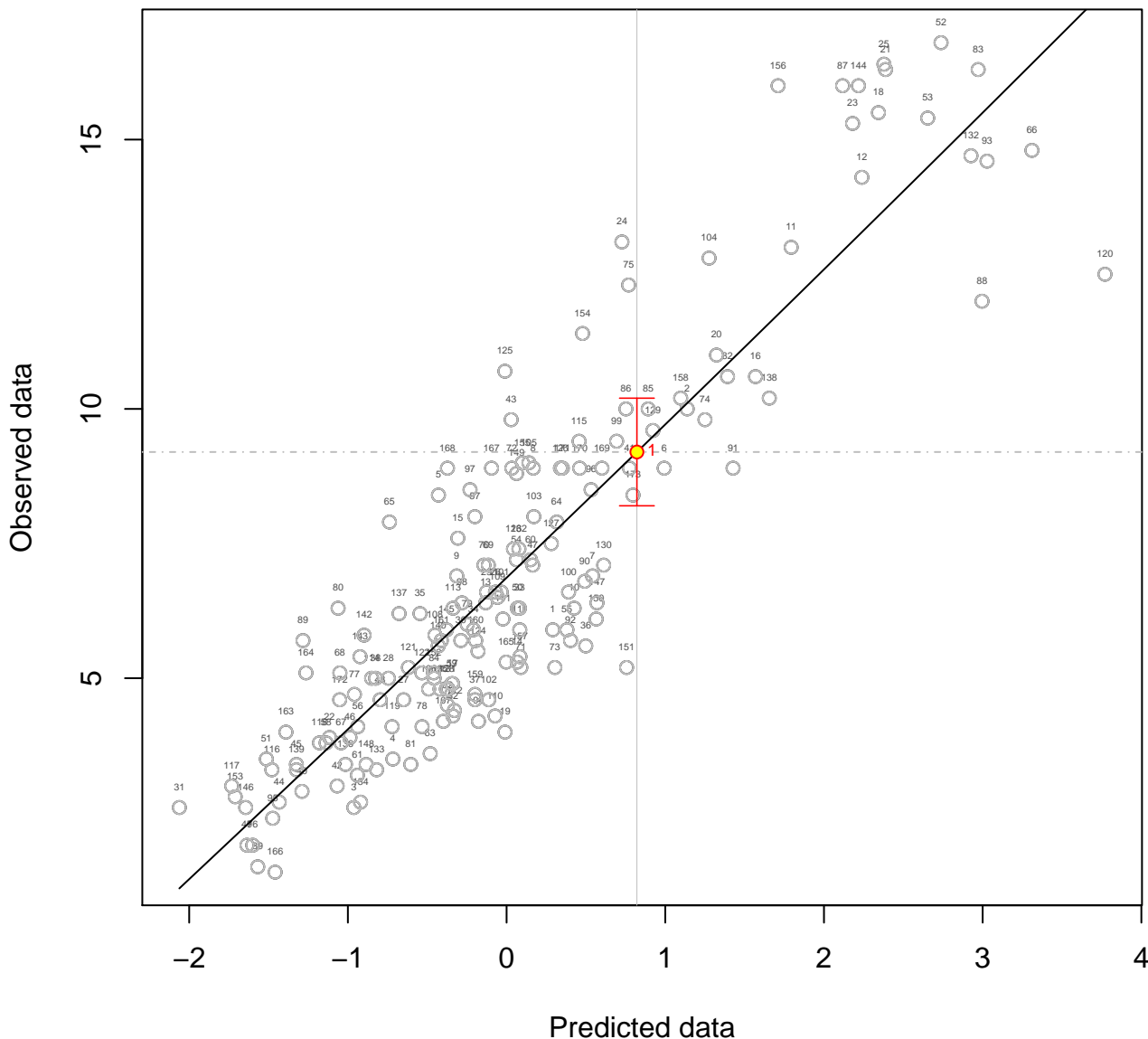

# ENTHAL (kJ/Kg)

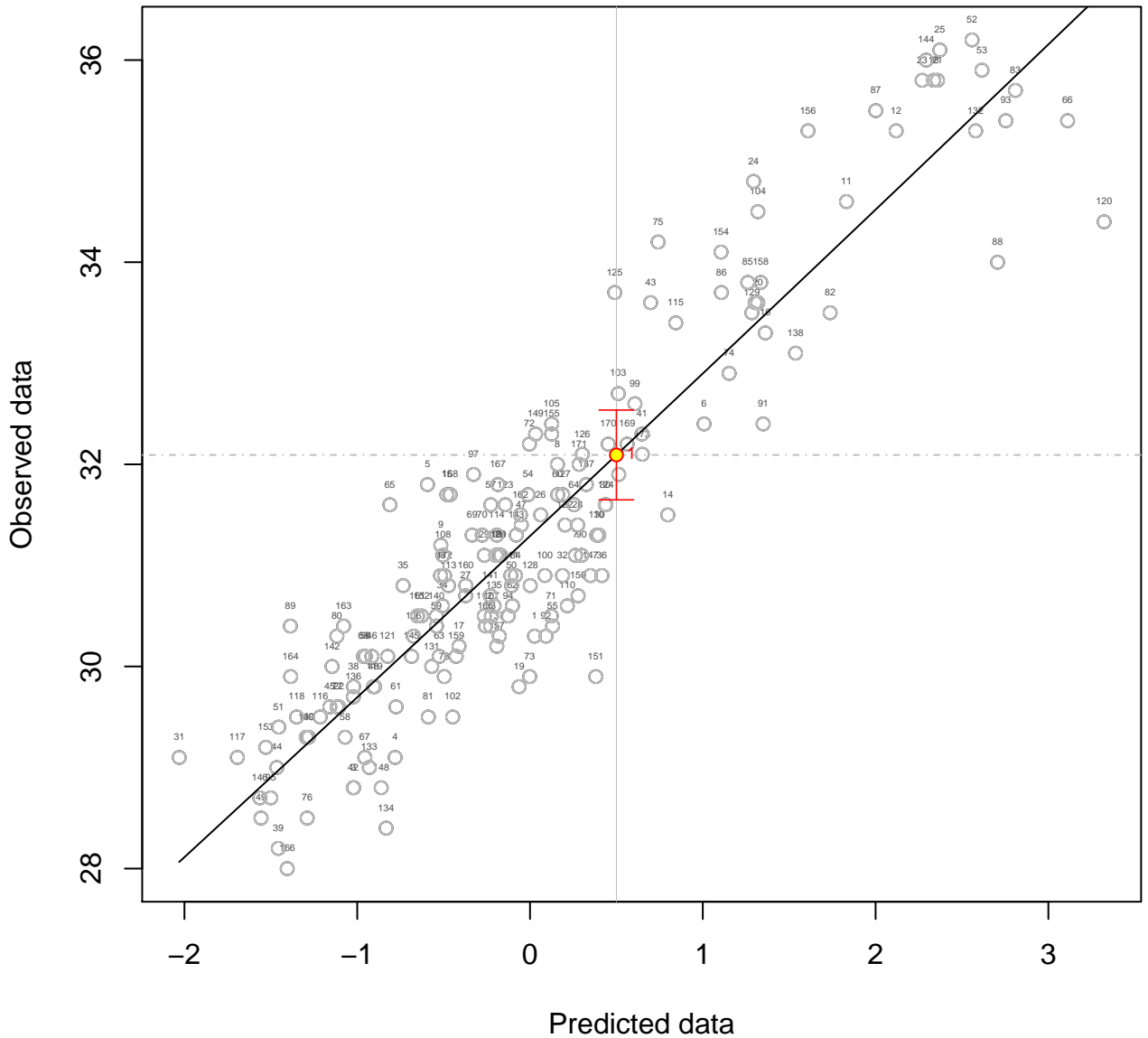

Supplement: Supplementary Material [file rsos192067supp1.zip › Supplementary Material S1-S5/S5_CLAMP_Vegora/CLAMP analysis/Run/Physg3arcAZ_GRIDMet3arAZ/Physg3arcAZ_GRIDMet3arAZ.pdf]

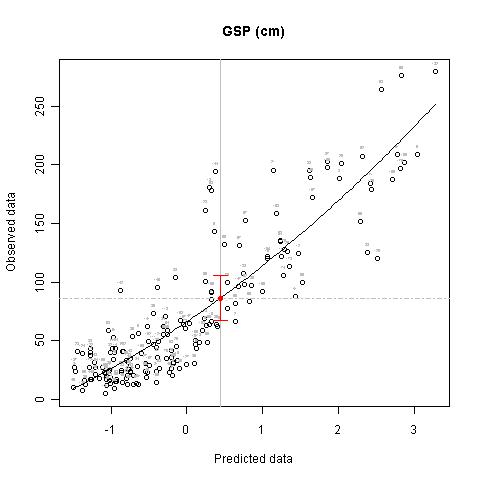

Supplement: Supplementary Material [file rsos192067supp1.zip › Supplementary Material S1-S5/S5_CLAMP_Vegora/CLAMP analysis/Run/Physg3arcAZ_GRIDMet3arAZ/GSP.jpg]
